# A novel thermostable alkaline histamine oxidase from *Glutamicibacter* sp. N1A3101, induced by histamine and its analogue betahistine

**DOI:** 10.1186/s13568-020-01115-2

**Published:** 2020-10-02

**Authors:** Hossein Sadeghi, Sareh Arjmand, Seyed Omid Ranaei Siadat, Jamshid Fooladi, Gholamhossein Ebrahimipour

**Affiliations:** 1 Department of Microbiology & Microbial Biotechnology, Faculty of Life Sciences and Biotechnology, Shahid Beheshti University, Tehran, Iran; 2 Protein Research Center, Shahid Beheshti University, Tehran, Iran; 3 Department of Biotechnology, Faculty of Biological Sciences, Alzahra University, Tehran, Iran

**Keywords:** Histamine oxidase, Histamine, Betahistine, *Glutamicibacter* sp. N1A3101

## Abstract

Biogenic amines (BAs) are low molecular weight organic bases formed by natural amino acids decarboxylation and trigger an array of toxicological effects in humans and animals. Bacterial amine oxidases enzymes are determined as practical tools to implement the rapid quantification of BAs in foods. Our study set out to obtain a new efficient, amine oxidase enzyme for developing new enzyme-based quantification of histamine. The soils from different sources were screened using histamine as sole carbon and nitrogen sources, and histamine oxidase producing bacteria were selected and identified using specific primers for histamine oxidase (HOD) gene. The HOD gene of six strains, out of 26 isolated histamine-utilizing bacteria, were amplified using our designed primers. The HOD enzyme from *Glutamicibacter sp*. N1A3101, isolated from nettle soil, was found to be thermostable and showed the highest substrate specificity toward the histamine and with no detected activity in the presence of putrescine, cadaverine, spermine, and spermidine. Its oxidation activity toward tyramine was lower than other HOD reported so far. The isolated enzyme was stable at 60 °C for 30 min and showed pH stability ranging from 6 to 9. Furthermore, we indicated the induction of identified HOD activity in the presence of betahistine as well, with nearly equal efficiency and without the consumption of the substrate. 
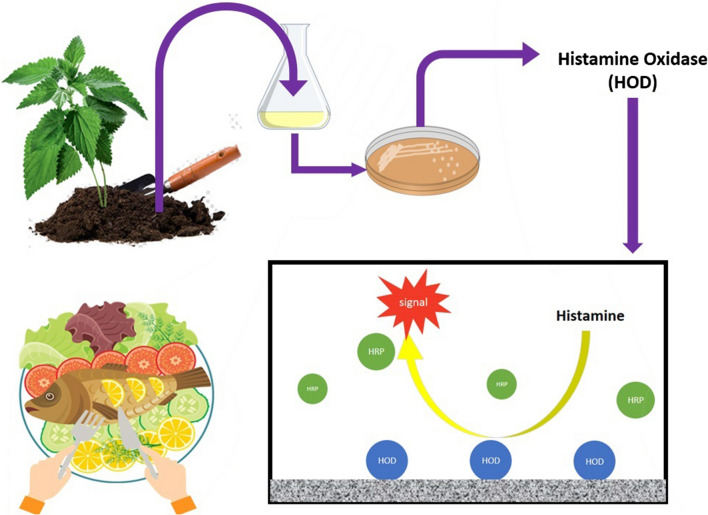

## Introduction

BAs are non-volatile, low molecular weight nitrogenous organic bases which are formed through decarboxylation of amino acids by amino acid-specific decarboxylases. These amines are naturally produced and degraded in humans, animals, plants, and microorganisms and result in numerous biological functions (Lee and Kim 2013; Alvarez and Moreno-arribas 2014; Velut et al. 2019). Amino acid decarboxylases are usually found in spoilage and other food microorganisms (Alvarez and Moreno-arribas 2014). Therefore, the BAs compounds are present to varying degrees in many of fermented and non-fermented foods, such as fishes, meats, cheeses, sausages, and wines. The major BAs related to spoilage in meat and fish are putrescine (1,4-diaminobutane), cadaverine (1,5-diaminopentane), tyramine (2-(p-hydroxyphenyl) ethylamine), and histamine (2-(4imidazolyl) ethylamine. The consumption of foods containing a high concentration of BAs may cause food intoxication and eventually leads to the organoleptic decay of the food products (Alvarez and Moreno-arribas 2014). Therefore, these compounds potentially are considered substantial indicators of food quality.

Among BAs, histamine is the most biochemically active amines and has been widely addressed as one of the fundamental primary amines in food analysis, especially in fishery industries (Ito et al. 2009). Scombroid and non-scombroid fish have been shown to have a high concentration of free l-histidine (histamine precursor) in their skeletal muscles (Biji et al. 2016). Inappropriate manipulation, freezing or conservation of fish can result in histidine decarboxylase enzyme activity and decarboxylation of histidine to histamine. Due to the adverse effects of histamine in human health, measuring its concentration is highly essential in evaluating food quality. Histamine assay could be an effective safety program in seafood processors to control histamine health hazards (Köse et al. 2011). Measurement of histamine in fish is traditionally performed by chromatographic methods, which is a time consuming, complicated, and expensive technique. Therefore developing rapid and cost-effective methods such as immuno-enzymatic assay, immuno-polymerase chain reaction method, chromogenic sensing, amperometric biosensing, and enzymatic assay have received major attention in recent years (Yang et al. 2019).

Among them, enzymatic determination of histamine using HOD enzymes is considered as an easy, effective strategy. The enzyme oxidatively deaminates the primary amine to imidazole acetaldehyde, hydrogen peroxide (H_2_O_2_), and ammonia (NH_3_). The generated H_2_O_2_ can simply be measured by spectrophotometry or fluorometry methods (Sekiguchi et al. 2001). Several copper-containing amine oxidases with high affinity to histamine have been found in *Aspergillus niger*AKU3302 (Suzuki et al. 1971), *Arthrobacter globiformis* (Shimizu et al. 1994), *Arthrobacter crystallopoietes* KAIT-B-007 (Sekiguchi et al. 2004), *Pisum sativum* (McGowan and Muir 1971) and mammalian sources (Pionetti 1974; Isobe et al. 1980). Some of them have been developed for the rapid detection of histamine in food processes. Although these enzymes exhibited a high affinity to many BAs such as tyramine, the enzyme with significant specificity to histamine is highly demanded.

We conducted this study under the assumption that in soil histamine co-exists with histamine-producing plants such as spinach and nettle. The soil near these plants can be considered as histamine oxidizing microorganism’s resources. Therefore, we have focused on the nettle grows soils to find a histamine oxidase with high specificity toward histamine. HOD need histamine induction to be expressed. Histamine is an expensive substrate for commercial production; therefore, we were looking for a cheaper alternative for enzyme production. Betahistine, a histamine analogue, showed promising results for the induction of HOD in its natural producing microorganism.

## Materials and methods

### Chemicals

Histamine dihydrochloride, putrescine dihydrochloride, cadaverine dihydrochloride, peroxidase, and N-Ethyl-N-(2-hydroxy-3-sulfopropyl)-3,5-dimethoxyaniline (DAOS) were obtained from Wako Pure Chemical (Japan). Spermidine trihydrochloride, and spermine tetrahydrochloride were purchased from Sigma Chemicals (USA). 4-aminoantipyrine was purchased from Biobasic Chemical Industries (Canada). Betahistine supplied from Shahredaru pharmaceutical co. (Iran). All reagents were analytical grade.

### Isolation of histamine metabolizing bacteria

To isolate the histamine-degrading bacteria, different soil samples around the roots of stinging nettle (*Urtica dioica*) were collected from different regions of Iran. A spoonful (approximately 5 g) of each soil sample was suspended in 100 ml of a screening medium consisting of 0.1% (w/v) histamine dihydrochloride, 0.05% (w/v) K_2_HPO_4_, 0.02% (w/v) MgSO_4_·7H_2_O, 0.001%(w/v) FeSO_4_·7H_2_O in tap water at pH 7.0. After incubation at 30 °C for 7 days, one mL of culture was inoculated in 100 mL of the same fresh medium and incubated for the next 2 days in the same condition. This step was repeated two times. After enrichment, 0.1 ml of the culture medium was plated out onto agar-solidified screening medium and incubated at 30 °C. The colonies appeared on the plates after 24–48 h, were transferred to 20 ml of Luria-Bertani (LB) medium (tryptone 10 g/L, yeast extract 5 g/L, NaCl 10 g/L), and cultivated at 30 °C, 115 rpm for 12 h.

### Amplification of putative HOD gene and extraction of HOD enzyme

The DNA sequence of HOD from the different bacterial genus (Additional file 1: Table S1) was obtained from NCBI GenBank and aligned by ClustalW online software (http://www.ebi.ac.uk/Tools/msa/clustalo). The identified conserved sequences were used to design the PCR primers for the amplification of newly isolated HOD genes. PCR amplifications were performed by applying the initial denaturation at 95 °C for 5 min, followed by 30 cycles of amplification (94 °C for 40 s, 52 °C for 40 s, and 72 °C for 60 s), and with 7 min of final extension at 72 °C. Genomic DNA of isolated histamine utilizing bacteria was used as template DNA and amplified using the designed primers, (forward: 5ʹ- AACTACGAYTACGGSTTCTACTGG-3ʹ and reverse: 5ʹ- GCATGATSGGCCAGTCCTC-3ʹ). After electrophoresis of PCR results, the bands representing the putative HOD gene were purified from agarose gel using QIAquick purification kit (Qiagen, Italy) and sent for sequencing at Bioneer Co. (Korea).

The resulting sequences were blast-analyzed to detect the sequence similarities. Besides, the PCR positive strains were cultivated in 10 ml of the screening medium for 12 h. The grown cells were harvested by centrifugation at 4,000*g* for 10 min at 4 °C and washed twice with 0.1 M phosphate buffer pH 7.0. The resulting cells were resuspended in the wash buffer and sonicated for a total of 5 min (2 s sonications followed by a 10 s intervals) at a power setting of 20 kHz under ice-cooling and using a bench-top sonicator. The cells and debris were removed by centrifugation at 12,000*g* for 10 min at 4 °C, and supernatants were collected and referred to as the crude enzyme extract.

### Determination of HOD activity

The HOD assay was performed according to Sekiguchi et al. (2004) with minor modifications. The assay mixture (170 μL) consisting of 1.2 mM histamine dihydrochloride, 1.47 mM DAOS, 2.2 mM 4–AA and one unit/mL horseradish peroxidase in 20 mM potassium phosphate buffer (pH 7.0) was preincubated at 37 °C for 5 min. The obtained cell lysate was diluted with 20 mM phosphate buffer (pH 7.0), and 30 μL of the sample was mixed in the assay mixture. The mixtures were incubated for 5 min at 37 °C, and the OD_600_ was measured using an Infinite M200 microtiter plate reader (Switzerland). The mixture without substrate was considered as negative control. One unit of activity was defined as the amount of enzyme which liberated 1 µmol of hydrogen peroxide per min under the specified conditions. The amount of H_2_O_2_ was calculated using the standard curve.

### Taxonomic identification of HOD producing bacterium

The isolated strain with the highest HOD activity was identified by 16S rRNA sequencing. The genomic DNA was amplified by PCR using the following primers of 16S rRNA; 27 F (5′-AGAGTTTGATCMTGGCTCAG-3′), and 1492 R (5′-GGYTACCTTGTTACGACTT-3′). The PCR program comprised initial denaturation at 95 °C for 5 min, followed by 30 cycles of amplification (94 °C for 40 s, 52 °C for 40 s, 72 °C for 60 s), and 7 min of final extension at 72 °C. PCR product was purified by the QIAquick purification kit (Quiagen, Italy) and sequenced by Bioneer Co. (Korea). The obtained sequence was compared with reference 16S rRNA gene sequences available in EzBioCloud database (https://www.ezbiocloud.net/). All the representative sequences were aligned using the Clustalx software package, and the phylogenetic tree was constructed using neighbor-joining method available in MEGA version 10 software. Bootstrap analysis based on 1000 replications was conducted for evaluating the confidence level of the branch nodes.

### Growth profile of selected bacterium and HOD production

The isolated bacterium was cultivated in LB medium at 30 °C and 115 rpm for 12 h. The grown cells were harvested by centrifugation at 10,000*g* for two min, washed twice with saline and diluted to match the 0.5 McFarland turbidity standard (OD_600_ = 0.13, approximately 10^8^ CFU/ml). Ten ml of the prepared dilution was used for inoculation of one L of basal salt medium (BSM) (0.05% K_2_HPO_4_, 0.02% MgSO_4_.6H_2_O, 0.001% FeSO_4_.7H_2_O, 0.001% CaCl_2_, and 0.1% (v/v) trace elements (70 mg ZnCl_2_, 100 mg MnCl_2_.4H_2_O, 200 mg CoCl_2_.6H_2_O, 100 mg NiCl_2_.6H_2_O, 20 mg CuCl_2_.2H2O, 50 mg, 50 mg NaMoO_4_.2H_2_O, 26 mg Na_2_SeO_3_.5H_2_O and one ml of 25% HCl in 1000 ml distilled water, pH 8.0)) containing 0.1% histamine dihydrochloride and 1% sodium acetate, and incubated for 18 h at 30 °C, and 115 rpm. Ten mL of cultures were aliquot at regular intervals for monitoring the cell growth by measuring the optical density at 600 nm. Cell lysates were used for the measurement of HOD activity.

### Substrate specificity analysis

Substrate specificity of the crude enzyme was checked by using different BAs, including histamine, tyramine, putrescine, cadaverine, spermine, and spermidine, with the final concentration of 1.2 mM. Enzymatic activity on each substrate was determined based on the method described earlier.

### Effect of temperature and pH on enzyme activity

The effect of temperature on isolated HOD was examined by incubating 30 µL of the diluted enzyme with 170 µL substrate solution (1.2 mM histamine dihydrochloride in 20 mM potassium phosphate buffer pH 7.0). After incubation for 10 min at the temperatures from 40 to 70 °C, the reaction mixture was boiled for 5 min to terminate the reaction. Then, 30 µL of mixtures were mixed with 170 µL chromogen solution, consisting of 1.47 mM DAOS, 2.2 mM 4–AA, and one unit/mL horseradish peroxidase in 20 mM potassium phosphate buffer (pH 7.0) and incubated for 5 min at 37 °C. Thermostability was investigated by incubating of the diluted enzyme at the temperatures from 40 to 70 °C for 10, 20, and 30 min.

The heat-treated enzyme solutions were immediately cooled in ice-bath, and the residual activity was assayed at 37 °C. The relative activity of the enzyme was calculated in comparison with non- heated samples at room temperature. The experiments were performed in triplicate.

The effect of pH on the HOD activity was studied using Britton Robinson buffer (buffering range 5.0 to 9.0) with a concentration of 40 mM. For pH stability analysis, the enzyme solution was diluted with Britton–Robinson buffer (buffering range 4.0–11.0). After incubation at 37 °C for 30 min, the enzyme solution was assayed in the standard reaction mixture for residual HOD activity.

### Study the effect of betahistine on the induction of HOD production

Histamine is relatively expensive, and for running the experiments and induction of HOD gene a cheaper but efficient source was highly desired. Therefore, we chose betahistine, a structural histamine analogue which is relatively cheaper and more affordable, to be assessed as the inducer. For this purpose, three experiments were designed and conducted as follow;

#### Experiment 1: Betahistine as sole carbon and nitrogen source

1% (v/v) of seed culture (OD_600_ = 0.13) inoculated to 20 ml of BSM containing 0.1% Betahistine as sole carbon and nitrogen source. The media were incubated for 24 h, at 30 °C and 115 rpm. Grown cells were harvested from the culture by centrifugation at 4,000*g* for 10 min at 4 °C, washed twice with a large volume of 0.1 M phosphate buffer (pH 7.0), and resuspended in the same buffer for performing HOD assay.

#### Experiment 2: Betahistine as the sole nitrogen source

1% (v/v) of seed culture (OD_600nm_ = 0.13) inoculated to 20 mL of BSM containing 1% acetate as sole carbon and 0.1% betahistine as sole nitrogen source. The cells were grown and prepared for HOD assay analysis, as explained above.

#### Experiment 3: Betahistine as inducer agent

1% (v/v) of seed culture (OD_600nm_ = 0.13) inoculated to 20 mL of BSM containing 1% acetate as sole carbon and ammonium chloride as the nitrogen source in the presence of 0.1% Betahistine, and the cells were prepared for HOD assay as explained.

For negative control, the BSM medium containing 1% acetate as carbon and 0.1% ammonium chloride as the nitrogen sources, was used. The BSM medium containing 1% acetate as carbon and 0.1% histamine dihydrochloride as the nitrogen source was used for positive control.

## Results

### PCR amplification of putative HOD gene, and molecular phylogeny of isolated microorganism

**Fig. 1:**
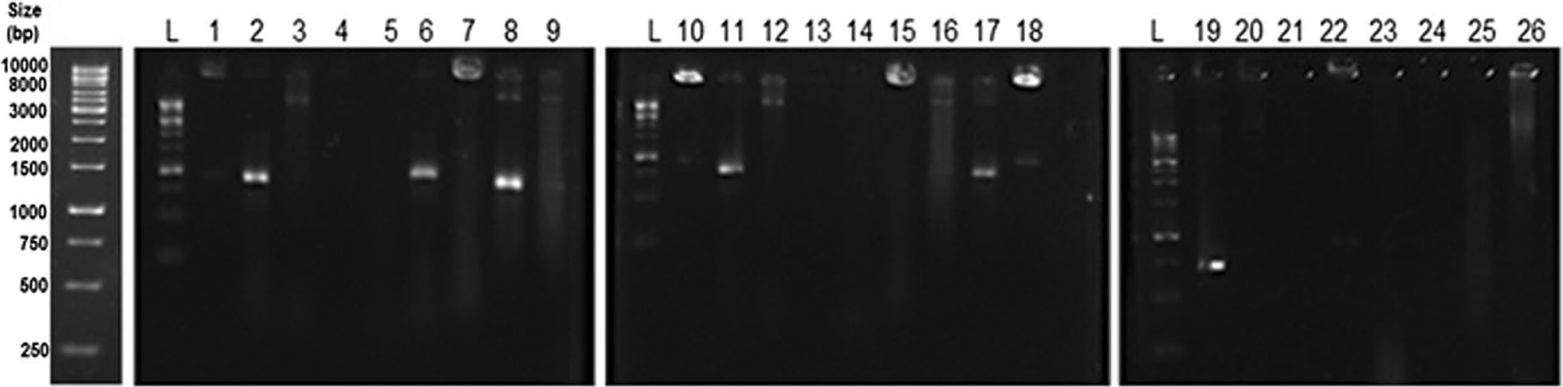
PCR amplification of genomic DNA from isolated histamine utilizing strains, using HOD primers. The amplified primers in lanes 2, 6, 8, 11, 17, and 19 were considered as putative sequences related to HOD. L, one-kb DNA marker

**Fig. 2:**
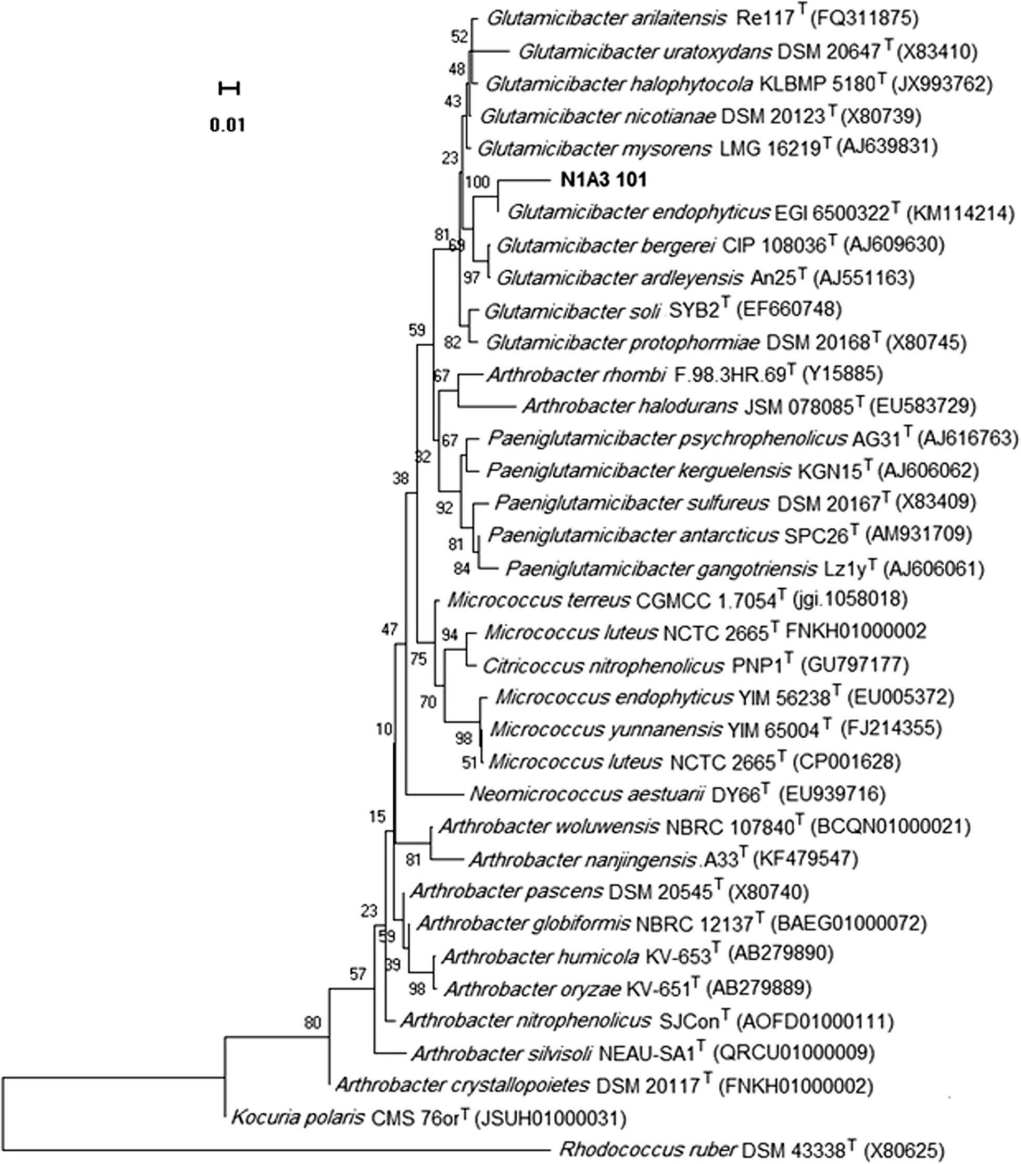
Phylogenetic tree showing the relationships between detected strain N1A3101 and other members of the genus *Glutamicibacter* and other related genera. The phylogenetic tree was generated using the Neighbour-joining method based on the Tamura-Nei model. Numbers at nodes are bootstrap values based on 1000 replicates. The sequence of 16S rRNA from *Rhodococcus ruber* DSM 43338^T^ was used as the outgroup. Type strains are indicated by a superscript T. The scale bar indicates a genetic distance of 0.01 substitutions per nucleotide position

The PCR results of the conserved area in six strains from the 26 isolated histamine-utilizing bacteria indicated a sharp band (~ 750–1000 bp) that were considered as a putative HOD gene and sent for sequencing (Fig. 1). The strain with the highest measured HOD activity was selected for the 16S rRNA gene analysis and identified as the *Glutamicibacter* sp. N1A3101. The phylogenetic position of the identified strain based on 16S rRNA gene sequence analysis has been depicted in Fig. 2. The physicochemical properties of *Glutamicibacter* sp. N1A3101 have been compared with its phylogenetically closet relative (*Glutamicibacter endophyticus*) in Table 1.

**Table 1:** Comparison of physicochemical characteristics of strain N1A3101 and its closest phylogenetic strain

Characteristic	*Glutamicibacter.sp* N1A3101	*G. endophyticu s*(Busse and Schumann 2019)
Gram staining	Positive	Positive
Catalase	+	+
Oxidase	–	–
Spore forming	–	–
Anaerobic growth	–	–
Motility	–	–
Temperature range for growth (°C)	5–40	5–35
NaCl range for growth (%)	0–10	0–13
Hydrolysis of:		
Starch	+	+
Casein	+	+
Urease	+	+
Gelatin	+	+
Nitrate reduction to nitrite	–	–
Utilization of:		
l-Arabinose	+	+
l-Lactose	–	–
Glycerol	+	+
d-Glucose	+	+
d-Fructose	+	+
Inulin	+	ND
Ascorbate	–	ND
Citrate utilization	+	+

The detected sequenced HOD gene of the strain N1A3101 showed the highest similarity with HOD form the *Arthrobacter globiformis* IFO12137 (99%), and *Arthrobacter crystallopoietes* KAIT B 007 (80%) strains, respectively.

### Time courses of cell growth and HOD production

**Fig. 3:**
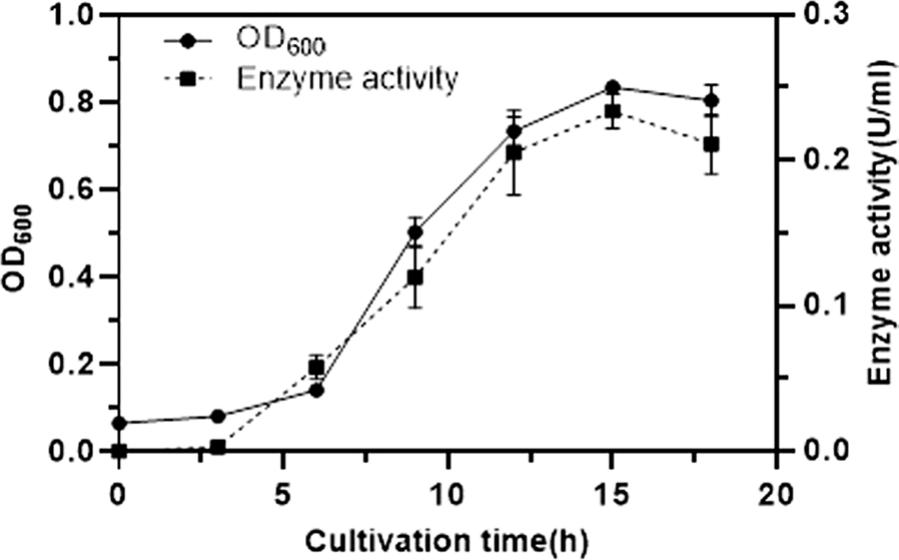
Time course of *Glutamicibacter* sp. N1A3101cell growth and HOD activity in BSM medium containing acetate and histamine dihydrochloride as the carbon and nitrogen sources, respectively. The error bars represent standard error (n = 3)

Time course of *Glutamicibacter* sp. N1A3101growth in BSM medium containing 1% acetate as carbon and 0.1% histamine dihydrochloride as nitrogen source has been shown in Fig. 3. After a six h of lag phase, bacterium started to grow exponentially, and at the same time, enzyme production increased parallel to the log phase. Maximum growth and enzyme production were observed after 12–15 h incubation.

### *Glutamicibacter* sp. *N1A3101* HOD exhibited substrate specificity for histamine

**Fig. 4:**
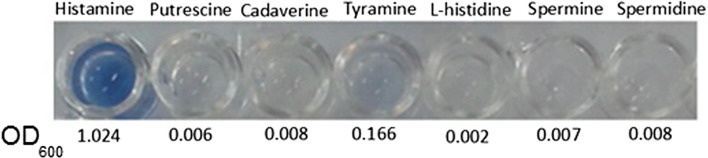
Substrate specificity of *Glutamicibacter* sp. N1A3101 HOD against several BAs (absorbance at 600 nm) indicated its high specificity for histamine

The crude enzyme showed a high oxidase activity toward histamine (0.2 U/mL), and a low degree of activity (~ 20% of histamine) against tyramine. No enzyme activity was measured for other tested substrates, including putrescine, cadaverine, spermine, spermidine, and l-histidine (Fig. 4).

### The effect of temperature and pH on HOD enzyme activity and stability

**Fig. 5:**
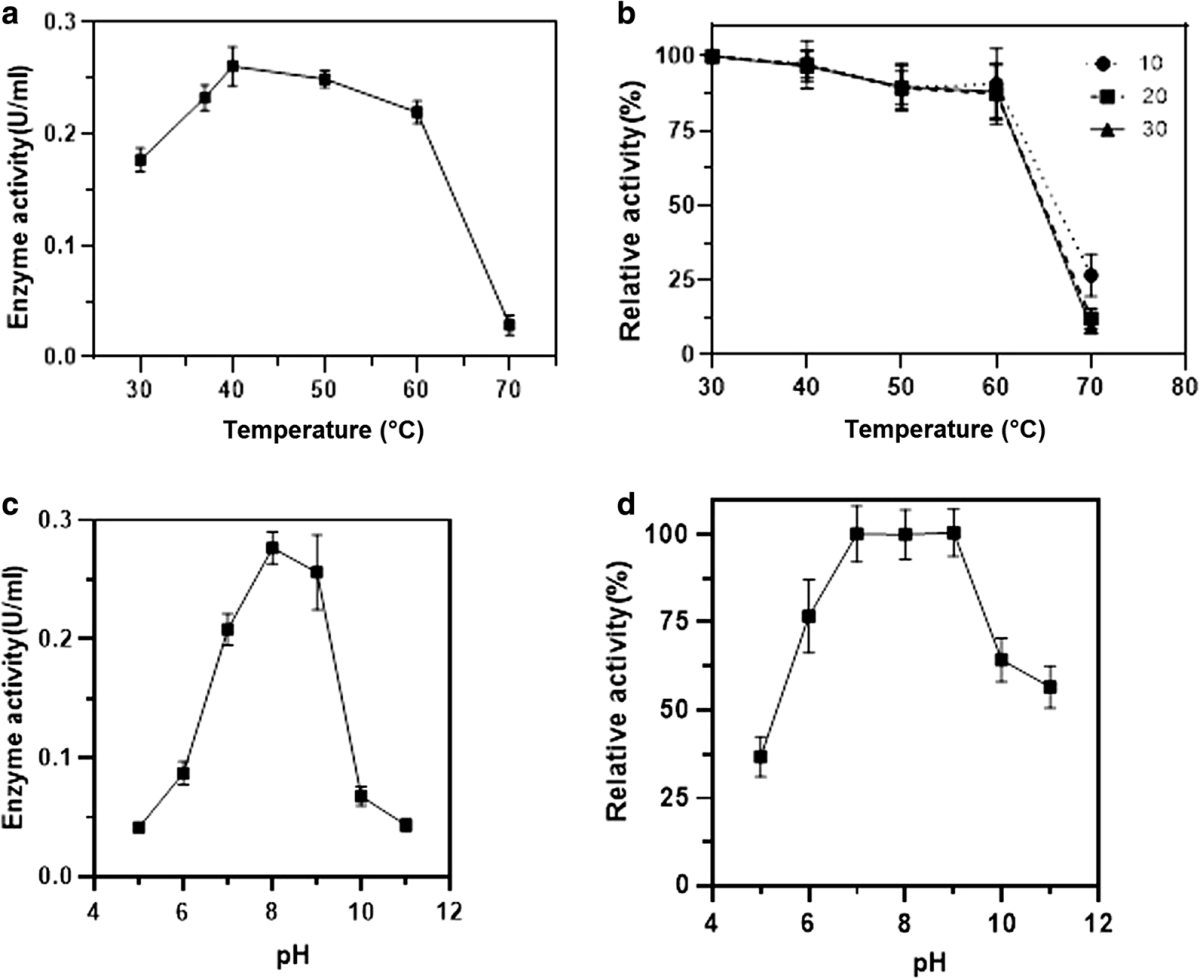
**a** Temperature profile, **b** thermal stability, **c** pH profile, and **d** pH stability of HOD from *Glutamibacter* sp. N1A3101. Thermal stability was measured after incubation of HOD for 10, 20, and 30 min at temperatures 30–70 °C, and pH stability measured after 10 min incubation at 40 °C. Error bars represent standard error (n = 3)

The maximum HOD activity was obtained at 40 °C (Fig. 5a). The thermal stability of the enzyme was shown in Fig. 5b. More than 90% of HOD activity remained after 30 min of heat treatment at 40–60 °C. However, a sharp decline was observed after 10 min of incubation at 70 °C.

As shown in Fig. 5c, d, the optimum pH for the HOD activity was found to be 8.0, and the enzyme showed high stability over the pH range of 7–9 at 40 °C for 10 min incubation.

### Betahistine induces HOD production

The results related to the effect of betahistine, as the carbon, nitrogen, or both sources, on the induction of HOD expression in *Glutamicibacter* sp. N1A3101 is summarized in Table 2. According to the obtained results, the bacterium did not consume betahistine as carbon or nitrogen source; therefore, no growth or HOD production was observed. However, in the medium containing acetate and NH_4_Cl as carbon and nitrogen sources (experiment 3); the addition of betahistine led to significant HOD enzyme production (0.1 U/ml), which was 50% of enzyme activity measured in positive control. No HOD activity was observed in the negative control.

**Table 2:** The effect of betahistine on HOD production in *Glutamicibacter* sp. N1A3101

Experiment #	Histamine	Betahistine	NH4Cl	Acetate	HOD activity (U/ml)
1	–	+	–	–	0
2	–	+	–	+	0
3	–	+	+	+	0.1
Control –	–	–	+	+	0
Control +	+	–	–	+	0.2

## Discussion

Histamine is a biologically active amine compound, produced in the different food products due to histidine decarboxylation, and may lead to allergies and food poisoning (Alvarez and Moreno-arribas 2014; Biji et al. 2016). Histamine quantification by enzymatic methods have been emerged as simple, fast and applicable strategies in food industries (Rosini et al. 2014). In the present study, a novel thermostable HOD enzyme whit high specificity toward histamine has been introduced and partially characterized.

We isolated several histamines metabolizing bacteria from nettle soils, using the enrichment culture method. For detection of HOD producing bacteria from other isolates, the PCR primers were designed based on the conserved sequences in other HOD genes.

Among the isolated HOD producing bacteria, the one with the highest Histamine oxidase activity was identified by 16 s rRNA sequencing and confirmed as the *Glutamicibacter* sp. N1A3101 (GenBank: MN047236.1). The detected HOD showed good specificity toward histamine; no oxidase activity was identified for other tested BAs, including putrescine, Cadaverine, Spermine, Spermidine and amino acid l-histidine. The only slight activity was seen for Tyramine substrate (20% of histamine), which was lower than other reported HODs (Shimizu et al. 1994; Sekiguchi et al. 2004).

According to the obtained results, this newly detected HOD is a thermostable alkaline enzyme with the maximum catalytic activity at 40 °C and pH 8. Furthermore, the enzyme well tolerates the pH values of 7 to 9. In terms of pH and temperature, this HOD enzyme showed similarity to the previously isolated HOD from *A. crystallopoietes* KAIT-B-007. However, it is notably considerable when compared to the other reported HODs. For instance, the HOD enzyme isolated from *A. niger* AKU3302 shows the maximum activity below 35 °C at pH 7, with low thermostability. The pH and temperature characteristics, besides its specificity, make the isolated HOD a useful candidate for biosensor application.

HOD is an inducible enzyme, and the presence of histamine substrate is necessary for enzyme production. Histamine is a relatively expensive material, and not cost-effective for large-scale production of HOD in the native microorganism. There are various strategies to reduce the production cost. For instance, optimizing the culture media, environmental conditions, cloning of the desired gene in another host, etc. (Chaikaew et al. 2015).

To address this issue, we investigated to find an alternative cheaper inducing agent. We hypothesized that compounds with histamine-like structures or those that are obtained from plants that produce histamine might induce gene expression through the effect on the gene regulatory sequences. Several candidate compounds such as spinach extract and nettle plant extract (data not represented), and a histamine chemical analogue (betahistine) were assayed, and betahistine was found that triggers the HOD production. Notably, betahistine was not consumed by *Glutamicibacter* sp. N1A3101 as nitrogen or carbon sources for growth, and functions explicitly as an inducer for HOD gene expression. The obtained HOD production with the betahistine needs to be optimized to be introduced as a suitable substrate for use in the industry.

To the best of our knowledge, no study has reported the betahistine as the inducer of HOD production. And further studies are necessary to describe the mechanism underlying the interaction of betahistine with the regulatory elements of HOD.

## Supplementary information

**Additional file 1: Table S1.** Alighnment of HOD DNA sequences from different bacterial genuses. The sequences used for primer design were highlighted in yellow.

## Data Availability

The datasets supporting the conclusions of this article are included within the article and its additional files. The GenBank accession number for 16S rRNA and newly isolated HOD gene reported in this manuscript are MN047236.1, and MT993978, respectively.

## Abbreviations

BAs: Biogenic amines
HOD: Histamine oxidase
LB: Luria–Bertani
BSM: Basal salt medium
DAOS BDOAS: N-Ethyl-N-(2-hydroxy-3-sulfopropyl)-3,5-dimethoxyaniline
