# Supplementary material for: A novel thermostable alkaline histamine oxidase from Glutamicibacter sp. N1A3101, induced by histamine and its analogue betahistine
Source: AMB Express. 2020 Oct 2;10:176. doi: 10.1186/s13568-020-01115-2 (PMC7532241; doi:10.1186/s13568-020-01115-2)
Supplement: Supplementary file 1 — Additional file 1: Table S1. Alighnment of HOD DNA sequences from different bacterial genuses. The sequences used for primer design were highlighted in yellow. [file 13568_2020_1115_MOESM1_ESM.pdf]

AMB Express

A novel thermostable alkaline histamine oxidase from *Glutamicibacter* sp. N1A3101, induced by histamine and its analogue betahistine

Hossein Sadeghi[1], Sareh Arjmand[2], Seyed Omid Ranaei Siadat[2,*], Jamshid Fooladi[3], Gholamhossein Ebrahimipour[1,*]

[1]Department of Microbiology & Microbial Biotechnology, Faculty of Life Sciences and Biotechnology, Shahid Beheshti University, G.C., Tehran, Iran

[2]Protein Research Center, Shahid Beheshti University, G. C., Tehran, Iran.

[3]Department of Biotechnology, Faculty of Biological Sciences, Alzahra University, Tehran, Iran

*Co-corresponding authors:

Seyed Omid Ranaei Siadat, Protein Research Center, Shahid Beheshti University, G. C., Tehran, Iran., Email o_ranaei@sbu.ac.ir, Tel +98 (21) 29905003. Gholamhossein Ebrahimipour, Department of Microbiology & Microbial Biotechnology, Faculty of Life Sciences and Biotechnology, Shahid Beheshti University, G.C., Tehran, Iran, Email g-ebrahimi@sbu.ac.ir. Tel +98 (21) 29902706.

Table S1 alighnment of HOD DNA sequences from different bacterial genuses. The sequences used for primer design were highlighted in yellow.

Bacterial genus	HOD gene sequence
Amycolatopsis thermoflava **N1165** Accessionn no: NZ_KI421511.1	GGGCAGCTGCCCGTGCTGGACGAGGAGTTCGCCCTGGTCGAGGAGGTGCT
Amycolatopsis japonica strain **MG417-CF17** Accession no: CP008953.1	GGGCAGCTGCCCGTGCTGGACGAGGAGTTCGCCCTGGTCGAGGAGGTGCT
Arthrobacter alpinus **ERGS4** Accession no: CP013200.1	GGCGAGTTGCCCGTGCTAGAAGAGGAATTTGAGGTTGTAGAGGCACTTCT
Arthrobacter sp. 31Y **K253DRAFT** Accession no: JAFW01000002.1	GGCGAGTTGCCCGTGCTAGAAGAGGAATTTGAGGTTGTAGAGGCACTTCT
Arthrobacter sp. **M2012083** Accession no: NZ_AKKK01000062.1	GGCGAGTTGCCCGTGCTAGAAGAGGAATTTGAGGTTGTAGAGGCACTTCT
Arthrobacter sp. **EpRS71** Accession no: LNUV01000006.1	GGCGAGTTGCCCGTGCTAGAAGAGGAATTTGAGGTTGTAGAGGCACTTCT
Arthrobacter crystallopoietes Accession no: AB240436.1	GGCGAGTTGCCCGTGCTAGAAGAGGAATTTGAGGTTGTAGAGGCACTTCT
Brevibacterium linens Accession no: CP014869.1	GGCGAGTTTCCCGTCATGGAAGAGGAGTTCGAAGTCGTCGAAGAGGTCCT
Brevibacterium sp. **YB235** Accession no: CP050153.1	GGCGAGTTTCCCGTCATGGAAGAGGAGTTCGAAGTCGTCGAAGAGGTCCT
Cryobacterium flavum **strain Hh8** Accession no: SOFD01000024.1	GGTGAACTGCCCGTGCTCGAGGAAGAATTCGGCGCCGTCGAAGACCTCCT
Cryobacterium sp. **MLB32 c47** Accession no: JPRS01000047.1	GGTGAACTGCCCGTGCTCGAGGAAGAATTCGGCGCCGTCGAAGACCTCCT
Cryobacterium luteum **strain Hh15** Accession no: SOFF01000030.1	GGTGAACTGCCCGTGCTCGAGGAAGAATTCGGCGCCGTCGAAGACCTCCT
Cryobacterium roopkundense Accession no: JPXF01000060.1	GGTGAACTGCCCGTGCTCGAGGAAGAATTCGGCGCCGTCGAAGACCTCCT
Kocuria indica Accession no: CP035504.1	GGGGAGCTGCCGGTGCTCGAGGAGGAGTTCGAGATGGTGGAGCAGATCCT
Kocuria flava Accession no: CP013254.1	GGGGAGCTGCCGGTGCTCGAGGAGGAGTTCGAGATGGTGGAGCAGATCCT
Microbacterium azadirachtae Accession no: JYIX 01000018.1	GGTCAGCCGCAGTACCTGTTCGAGGAGTACGAGCGCGCCGAGGCCATCGC
Microbacterium oxydans Accession no:CP031422.1	GGTCAGCCGCAGTACCTGTTCGAGGAGTACGAGCGCGCCGAGGCCATCGC
Microbacterium trichothecenolyticum Accession no: JYJA01000039.1	GGTCAGCCGCAGTACCTGTTCGAGGAGTACGAGCGCGCCGAGGCCATCGC
Microbacterium foliorum Accession no: JYIU01000019.1	GGTCAGCCGCAGTACCTGTTCGAGGAGTACGAGCGCGCCGAGGCCATCGC
Microbacterium mangrove Accession no: JTDK01000001.1	GGTCAGCCGCAGTACCTGTTCGAGGAGTACGAGCGCGCCGAGGCCATCGC
Nocardiopsis sp. **NRRL B-16309** Accession no: LGEC01000081.1	GGCCAACTTCCCGTCCTCGACGAGGAGTTCGCCCTGGTGGAGGAGGTCCT
Paenarthrobacter aurescens Accession no: CP000474.1	GGCGAATTGCCCGTGTTGGAAGAGGAATTTGAGGTTGTGGAGACGCTCCT
PseudArthrobacter sp. Accession no: CP041198.1	GGAGAGCTGCCGGTCCTCGAGGAGGAGTTCGAGGTAGTGGAATCCCTCCT
Pseudarthrobacter chlorophenolicus **A6** Accession no: CP001341.1	GGAGAGCTGCCGGTCCTCGAGGAGGAGTTCGAGGTAGTGGAATCCCTCCT
Rhodococcus jostii **RHA1** Accession no: CP000431.1	GGTCAGATGCCCGTCCTGGACGAGGAGTTCGAACTCGTCGAGCAGGTACT
Rhodococcus opacus **1CP** Accession no: CP009111.1	GGTCAGATGCCCGTCCTGGACGAGGAGTTCGAACTCGTCGAGCAGGTACT
Rhodococcus ruber Accession no: CCSD01000085.1	GGTCAGATGCCCGTCCTGGACGAGGAGTTCGAACTCGTCGAGCAGGTACT
Rhodococcus wratislaviensis Accession no:BHYM01000005.1	GGTCAGATGCCCGTCCTGGACGAGGAGTTCGAACTCGTCGAGCAGGTACT
Rhodococcus sp.**SC4 SC4** Accession no: LSBM01000283.1	GGTCAGATGCCCGTCCTGGACGAGGAGTTCGAACTCGTCGAGCAGGTACT
Saccharothrix espanaensis Accession no: He804045.1	GGCCAGCCCGCGATCCTGTTCGAGGAGTACGACCGCAGCGCCGAGCTGGT
Sinomonas atrocyanea Accession no: CP014518.1	GGCGAGCTGCCCGTGCTCGAGGAGGAGTTCGAGGCGGTCGAGGAGATCCT
Streptomyces ossamyceticus Accession no: NZ_RJKY01000001.1	GGGCAACTCCCCATCCTGGAGGAGGAGTTCGATCTGATCGAGGAGATCGT
Streptomyces fulvoviolaceus Accession no: NZ_JOEY01000080.1	GGGCAACTCCCCATCCTGGAGGAGGAGTTCGATCTGATCGAGGAGATCGT
Streptomyces torulosus Accession no: NZ_LIRK01000041.1	GGGCAACTCCCCATCCTGGAGGAGGAGTTCGATCTGATCGAGGAGATCGT
Streptomyces xylophagus Accession no: NZ_JNW001000006.1	GGGCAACTCCCCATCCTGGAGGAGGAGTTCGATCTGATCGAGGAGATCGT
	** * * ** ** * * * *
Amycolatopsis thermoflava **N1165** Accessionn no: NZ_KI421511.1	CGCCAAGGACGAGCGGTGGCTGGCGGCGCTGGCCGCCCGCGACCTGGACG
Amycolatopsis japonica strain MG417-CF17 Accession no: CP008953.1	CGCCAAGGACGAGCGGTGGCTGGCGGCGCTGGCCGCCCGCGACCTGGACG
Arthrobacter alpinus **ERGS4** Accession no: CP013200.1	AGCAACTCACCCCGAATGGCTAGAGGCTCTGGCCGCCCGGAACCTGGCGG
Arthrobacter sp. 31Y K253DRAFT Accession no: JAFW01000002.1	AGCAACTCACCCCGAATGGCTAGAGGCTCTGGCCGCCCGGAACCTGGCGG
Arthrobacter sp. M2012083 Accession no: NZ_AKKK01000062.1	AGCAACTCACCCCGAATGGCTAGAGGCTCTGGCCGCCCGGAACCTGGCGG
Arthrobacter sp. **EpRS71** Accession no: LNUV01000006.1	AGCAACTCACCCCGAATGGCTAGAGGCTCTGGCCGCCCGGAACCTGGCGG
Arthrobacter crystallopoietes Accession no: AB240436.1	AGCAACTCACCCCGAATGGCTAGAGGCTCTGGCCGCCCGGAACCTGGCGG
Brevibacterium linens Accession no: CP014869.1	CGCCACGGATCCGACGTGGCTGGCCAGCCTGGAGAAGCGCGGACTGTCCG
Brevibacterium sp. **YB235** Accession no: CP050153.1	CGCCACGGATCCGACGTGGCTGGCCAGCCTGGAGAAGCGCGGACTGTCCG
Cryobacterium flavum **strain Hh8** Accession no: SOFD01000024.1	CACAACGGATGCCCGCTGGCGGGCCGCACTCGCAGCCCGCGGCCTCGACG
Cryobacterium sp. **MLB32 c47** Accession no: JPRS01000047.1	CACAACGGATGCCCGCTGGCGGGCCGCACTCGCAGCCCGCGGCCTCGACG
Cryobacterium luteum **strain Hh15** Accession no: SOFF01000030.1	CACAACGGATGCCCGCTGGCGGGCCGCACTCGCAGCCCGCGGCCTCGACG
Cryobacterium roopkundense Accession no: JPXF01000060.1	CACAACGGATGCCCGCTGGCGGGCCGCACTCGCAGCCCGCGGCCTCGACG
Kocuria indica Accession no: CP035504.1	CTCCACGGACGAGCGCTGGCTGGCCGCGCTGCAGAAGCGCAACCTGCCCG

Kocuria flava Accession no: CP013254.1	CTCCACGGACGAGCGCTGGCTGGCCGCGCTGCAGAAGCGCAACCTGCCCG
Microbacterium azadirachtae Accession no: JYIX 01000018.1	CAAGGCCTCCCCCGAATGGCGTGCCGCGATGGAGCGCCGCGGACTCGCCG
Microbacterium oxydans Accession no:CP031422.1	CAAGGCCTCCCCCGAATGGCGTGCCGCGATGGAGCGCCGCGGACTCGCCG
Microbacterium trichothecenolyticum Accession no: JYJA01000039.1	CAAGGCCTCCCCCGAATGGCGTGCCGCGATGGAGCGCCGCGGACTCGCCG
Microbacterium foliorum Accession no: JYIU01000019.1	CAAGGCCTCCCCCGAATGGCGTGCCGCGATGGAGCGCCGCGGACTCGCCG
Microbacterium mangrove Accession no: JTDK01000001.1	CAAGGCCTCCCCCGAATGGCGTGCCGCGATGGAGCGCCGCGGACTCGCCG
Nocardiopsis sp. **NRRL B-16309** Accession no: LGEC01000081.1	GTCCACCGACGAGGACTGGCTGGCCGCCCTGCGCGCCCGCGACCTGGACC
Paenarthrobacter aurescens Accession no: CP000474.1	TGCAACCGACGAGCGGTGGCTCGCCGCACTCGCAGCCCGGGACCTGGACG
PseudArthrobacter sp. Accession no: CP041198.1	GGCCACCGACGAACGGTGGCTGAAGGCACTCGCGGACCGGGGACTCGACG
Pseudarthrobacter chlorophenolicus A6 Accession no: CP001341.1	GGCCACCGACGAACGGTGGCTGAAGGCACTCGCGGACCGGGGACTCGACG
Rhodococcus jostii **RHA1** Accession no: CP000431.1	GGCCGCCGACGAACGCTGGCTCGCCGCGCTCGCCGCCCGCGGACTCGACG
Rhodococcus opacus **1CP** Accession no: CP009111.1	GGCCGCCGACGAACGCTGGCTCGCCGCGCTCGCCGCCCGCGGACTCGACG
Rhodococcus ruber Accession no: CCSD01000085.1	GGCCGCCGACGAACGCTGGCTCGCCGCGCTCGCCGCCCGCGGACTCGACG
Rhodococcus wratislaviensis Accession no:BHYM01000005.1	GGCCGCCGACGAACGCTGGCTCGCCGCGCTCGCCGCCCGCGGACTCGACG
Rhodococcus sp.SC4 **SC4** Accession no: LSBM01000283.1	GGCCGCCGACGAACGCTGGCTCGCCGCGCTCGCCGCCCGCGGACTCGACG
Saccharothrix espanaensis Accession no: He804045.1	CAAGGCCGACCCGCGCTGGCAGGCGGCGATGCGCCGACGTGGCGTCGAGG
Sinomonas atrocyanea Accession no: CP014518.1	GGGCGGCGATGCCCGCTGGCTCGAGGCCCTCGCAGCACGCGGCCTCGACC
Streptomyces ossamyceticus Accession no: NZ_RJKY01000001.1	GGCCGCCGACGAGGGATGGCGGGCGGCGCTCGCCCGGCGCGGCCTCGACG
Streptomyces fulvoviolaceus Accession no: NZ_JOEY01000080.1	GGCCGCCGACGAGGGATGGCGGGCGGCGCTCGCCCGGCGCGGCCTCGACG
Streptomyces torulosus Accession no: NZ_LIRK01000041.1	GGCCGCCGACGAGGGATGGCGGGCGGCGCTCGCCCGGCGCGGCCTCGACG
Streptomyces xylophagus Accession no: NZ_JNW001000006.1	GGCCGCCGACGAGGGATGGCGGGCGGCGCTCGCCCGGCGCGGCCTCGACG
	**** * ** *
Amycolatopsis thermoflava **N1165** Accessionn no: NZ_KI421511.1	TGGCGAAGGTGCGGGTC------GCGCCGCTGTCGGCCGGGGTGTTCGAC
Amycolatopsis japonica **strain MG417-CF17** Accession no: CP008953.1	TGGCGAAGGTGCGGGTC------GCGCCGCTGTCGGCCGGGGTGTTCGAC
Arthrobacter alpinus **ERGS4** Accession no: CP013200.1	TGGAAAAGGTGCGGGTG------GCGCCGCTGTCCGCAGGCGTCTTTGAA
Arthrobacter sp. 31Y K253DRAFT Accession no: JAFW01000002.1	TGGAAAAGGTGCGGGTG------GCGCCGCTGTCCGCAGGCGTCTTTGAA
Arthrobacter sp. M2012083 Accession no: NZ_AKKK01000062.1	TGGAAAAGGTGCGGGTG------GCGCCGCTGTCCGCAGGCGTCTTTGAA
Arthrobacter sp. **EpRS71** Accession no: LNUV01000006.1	TGGAAAAGGTGCGGGTG------GCGCCGCTGTCCGCAGGCGTCTTTGAA
Arthrobacter crystallopoietes Accession no: AB240436.1	TGGAAAAGGTGCGGGTG------GCGCCGCTGTCCGCAGGCGTCTTTGAA
Brevibacterium linens Accession no: CP014869.1	TCGAGGAGGTGCGGGTG------GCACCGCTGTCGGCCGGTGTCTTCGAG
Brevibacterium sp. **YB235** Accession no: CP050153.1	TCGAGGAGGTGCGGGTG------GCACCGCTGTCGGCCGGTGTCTTCGAG
Cryobacterium flavum **strain Hh8** Accession no: SOFD01000024.1	TGGCCACCGTGCGCGTC------GCTCCCCTCTCCGCGGGGGTATTCGAA
Cryobacterium sp. **MLB32 c47** Accession no: JPRS01000047.1	TGGCCACCGTGCGCGTC------GCTCCCCTCTCCGCGGGGGTATTCGAA
Cryobacterium luteum **strain Hh15** Accession no: SOFF01000030.1	TGGCCACCGTGCGCGTC------GCTCCCCTCTCCGCGGGGGTATTCGAA
Cryobacterium roopkundense Accession no: JPXF01000060.1	TGGCCACCGTGCGCGTC------GCTCCCCTCTCCGCGGGGGTATTCGAA
Kocuria indica Accession no: CP035504.1	TGGAGAAGGTGCGGGTG------GCGCCGCTGTCCGCCGGGGTGTTCGAG
Kocuria flava Accession no: CP013254.1	TGGAGAAGGTGCGGGTG------GCGCCGCTGTCCGCCGGGGTGTTCGAG
Microbacterium azadirachtae Accession no: JYIX 01000018.1	AGCACATCGATCTCGCGTTCTGCGGGCCACTCGCCCCCGGATACACCGGC
Microbacterium oxydans Accession no:CP031422.1	AGCACATCGATCTCGCGTTCTGCGGGCCACTCGCCCCCGGATACACCGGC
Microbacterium trichothecenolyticum Accession no: JYJA01000039.1	AGCACATCGATCTCGCGTTCTGCGGGCCACTCGCCCCCGGATACACCGGC
Microbacterium foliorum Accession no: JYIU01000019.1	AGCACATCGATCTCGCGTTCTGCGGGCCACTCGCCCCCGGATACACCGGC
Microbacterium mangrove Accession no: JTDK01000001.1	AGCACATCGATCTCGCGTTCTGCGGGCCACTCGCCCCCGGATACACCGGC
Nocardiopsis sp. **NRRL B-16309** Accession no: LGEC01000081.1	CCTCCACCGTCCGGGTG------GCCCCGCTGTCGGCCGGGGTCTACGAC
Paenarthrobacter aurescens Accession no: CP000474.1	TGAAAAATGTTCGCGTT------GCTCCGTTGTCCGCGGGAGTTTTTGAG
PseudArthrobacter sp. Accession no: CP041198.1	TCAAGAAAGTCCGCGTC------GCGCCGCTCTCGGCCGGCGTGTTCGAA
Pseudarthrobacter chlorophenolicus A6 Accession no: CP001341.1	TCAAGAAAGTCCGCGTC------GCGCCGCTCTCGGCCGGCGTGTTCGAA
Rhodococcus jostii **RHA1** Accession no: CP000431.1	TCGCCGACGTCCGGGTC------GCTCCCCTCTCCGCGGGTGTCTTCGAC
Rhodococcus opacus **1CP** Accession no: CP009111.1	TCGCCGACGTCCGGGTC------GCTCCCCTCTCCGCGGGTGTCTTCGAC
Rhodococcus ruber Accession no: CCSD01000085.1	TCGCCGACGTCCGGGTC------GCTCCCCTCTCCGCGGGTGTCTTCGAC
Rhodococcus wratislaviensis Accession no:BHYM01000005.1	TCGCCGACGTCCGGGTC------GCTCCCCTCTCCGCGGGTGTCTTCGAC
Rhodococcus sp.SC4 **SC4** Accession no: LSBM01000283.1	TCGCCGACGTCCGGGTC------GCTCCCCTCTCCGCGGGTGTCTTCGAC
Saccharothrix espanaensis Accession no: He804045.1	ACTGGTC---GCTGACCTTCATCGGCCCGTTGTCCCCCGGTTTCTTCGA-
Sinomonas atrocyanea Accession no: CP014518.1	CGGCGACGGTCCGCGTC------GCCCCGCTCTCCGCGGGCGTCTTCGAG
Streptomyces ossamyceticus Accession no: NZ_RJKY01000001.1	GGGCGCTGGTGCGGGTC------GCCCCGCTCTCCGCGGGGGTGTACGCG
Streptomyces fulvoviolaceus Accession no: NZ_JOEY01000080.1	GGGCGCTGGTGCGGGTC------GCCCCGCTCTCCGCGGGGGTGTACGCG

Streptomyces torulosus Accession no: NZ_LIRK01000041.1	GGGCGCTGGTGCGGGTC------GCCCCGCTCTCCGCGGGGTGTACGCG
Streptomyces xylophagus Accession no: NZ_JNW001000006.1	GGGCGCTGGTGCGGGTC------GCCCCGCTCTCCGCGGGGTGTACGCG
	* * ** * * * ** *
***Amycolatopsis thermoflava* N1165** Accessionn no: NZ_KI421511.1	------TACCCGGAGG--AGACCGGGCGGCGCATCCTCCGCGGCCTGGCG
***Amycolatopsis japonica* strain MG417-CF17** Accession no: CP008953.1	------TACCCGGAGG--AGACCGGGCGGCGCATCCTCCGCGGCCTGGCG
***Arthrobacter alpinus* ERGS4** Accession no: CP013200.1	------TACCCTGAGG--AAAAAGGCCGCCGTATTCTCCGTGGGCTGGCG
Arthrobacter sp. 31Y K253DRAFT Accession no: JAFW01000002.1	------TACCCTGAGG--AAAAAGGCCGCCGTATTCTCCGTGGGCTGGCG
Arthrobacter sp. M2012083 Accession no: NZ_AKKK01000062.1	------TACCCTGAGG--AAAAAGGCCGCCGTATTCTCCGTGGGCTGGCG
***Arthrobacter sp.* EpRS71** Accession no: LNUV01000006.1	------TACCCTGAGG--AAAAAGGCCGCCGTATTCTCCGTGGGCTGGCG
Arthrobacter crystallopoietes Accession no: AB240436.1	------TACCCTGAGG--AAAAAGGCCGCCGTATTCTCCGTGGGCTGGCG
Brevibacterium linens Accession no: CP014869.1	------TACCCGGAGG--AGAAGGGACGGCGGATCCTGCGCGGGCTGGCG
***Brevibacterium sp.* YB235** Accession no: CP050153.1	------TACCCGGAGG--AGAAGGGACGGCGGATCCTGCGCGGGCTGGCG
***Cryobacterium flavum* strain Hh8** Accession no: SOFD01000024.1	------TACCCGAACG--AGAAGGGCCGCCGCATTCTGCGCGGCCTCGCC
***Cryobacterium sp.* MLB32 c47** Accession no: JPRS01000047.1	------TACCCGAACG--AGAAGGGCCGCCGCATTCTGCGCGGCCTCGCC
***Cryobacterium luteum* strain Hh15** Accession no: SOFF01000030.1	------TACCCGAACG--AGAAGGGCCGCCGCATTCTGCGCGGCCTCGCC
Cryobacterium roopkundense Accession no: JPXF01000060.1	------TACCCGAACG--AGAAGGGCCGCCGCATTCTGCGCGGCCTCGCC
Kocuria indica Accession no: CP035504.1	------TACCCGGAGG--AGAAGGGCCGGCGCATGCTGCGTGGGCTCGCG
Kocuria flava Accession no: CP013254.1	------TACCCGGAGG--AGAAGGGCCGGCGCATGCTGCGTGGGCTCGCG
Microbacterium azadirachtae Accession no: JYIX 01000018.1	------CGCGCCGACG--AGGTGGGGCGACGCGTGATCCGCTCGCTCACG
Microbacterium oxydans Accession no:CP031422.1	------CGCGCCGACG--AGGTGGGGCGACGCGTGATCCGCTCGCTCACG
Microbacterium trichothecenolyticum Accession no: JYJA01000039.1	------CGCGCCGACG--AGGTGGGGCGACGCGTGATCCGCTCGCTCACG
Microbacterium foliorum Accession no: JYIU01000019.1	------CGCGCCGACG--AGGTGGGGCGACGCGTGATCCGCTCGCTCACG
Microbacterium mangrove Accession no: JTDK01000001.1	------CGCGCCGACG--AGGTGGGGCGACGCGTGATCCGCTCGCTCACG
***Nocardiopsis sp.* NRRL B-16309** Accession no: LGEC01000081.1	GACGAGTACCCCGAGG--AACGCGGCCGCCGCATCCTGCGCGGACTCGCC
Paenarthrobacter aurescens Accession no: CP000474.1	------TACGCCGAAG--AGAAAGGCCGCCGCATCCTCCGTGGGCTCGCG
PseudArthrobacter sp. Accession no: CP041198.1	------TACCCGGAGG--AAAAGGGGCGCCGGATCCTCCGCGGGCTGGCG
Pseudarthrobacter chlorophenolicus A6 Accession no: CP001341.1	------TACCCGGAGG--AAAAGGGGCGCCGGATCCTCCGCGGGCTGGCG
***Rhodococcus jostii* RHA1** Accession no: CP000431.1	------TACCCCGGCG--AGACGGGCACGCGGATCCTCCGGGGGCTCGCG
***Rhodococcus opacus* 1CP** Accession no: CP009111.1	------TACCCCGGCG--AGACGGGCACGCGGATCCTCCGGGGGCTCGCG
Rhodococcus ruber Accession no: CCSD01000085.1	------TACCCCGGCG--AGACGGGCACGCGGATCCTCCGGGGGCTCGCG
Rhodococcus wratislaviensis Accession no:BHYM01000005.1	------TACCCCGGCG--AGACGGGCACGCGGATCCTCCGGGGGCTCGCG
***Rhodococcus sp.SC4* SC4** Accession no: LSBM01000283.1	------TACCCCGGCG--AGACGGGCACGCGGATCCTCCGGGGGCTCGCG
Saccharothrix espanaensis Accession no: He804045.1	------CGAGCCGCTGCTGCGCGAGCGGC-GCGTGCTGCGGGCGTTGACG
Sinomonas atrocyanea Accession no: CP014518.1	------TACCCCGAGG--AAAAGGGCCGCAGGATCCTGCGCGGCCTGGCC
Streptomyces ossamyceticus Accession no: NZ_RJKY01000001.1	GACGAGTACCCCGACG--AGGCCGGACGCCGTGTCCTGCGCGGCCTCGCC
Streptomyces fulvoviolaceus Accession no: NZ_JOEY01000080.1	GACGAGTACCCCGACG--AGGCCGGACGCCGTGTCCTGCGCGGCCTCGCC
Streptomyces torulosus Accession no: NZ_LIRK01000041.1	GACGAGTACCCCGACG--AGGCCGGACGCCGTGTCCTGCGCGGCCTCGCC
Streptomyces xylophagus Accession no: NZ_JNW001000006.1	GACGAGTACCCCGACG--AGGCCGGACGCCGTGTCCTGCGCGGCCTCGCC
	* * * * * * ** * *
***Amycolatopsis thermoflava* N1165** Accessionn no: NZ_KI421511.1	TTCGTGCAGGAACACCCTGGGGACCACGCGTGGGCGCACCCGGTCGACGG
***Amycolatopsis japonica* strain MG417-CF17** Accession no: CP008953.1	TTCGTGCAGGAACACCCTGGGGACCACGCGTGGGCGCACCCGGTCGACGG
***Arthrobacter alpinus* ERGS4** Accession no: CP013200.1	TTCTACCAAGACTTCCCGGAGGACAGCGCATGGGCGCATCCTGTGGATGG
Arthrobacter sp. 31Y K253DRAFT Accession no: JAFW01000002.1	TTCTACCAAGACTTCCCGGAGGACAGCGCATGGGCGCATCCTGTGGATGG
Arthrobacter sp. M2012083 Accession no: NZ_AKKK01000062.1	TTCTACCAAGACTTCCCGGAGGACAGCGCATGGGCGCATCCTGTGGATGG
***Arthrobacter sp.* EpRS71** Accession no: LNUV01000006.1	TTCTACCAAGACTTCCCGGAGGACAGCGCATGGGCGCATCCTGTGGATGG
Arthrobacter crystallopoietes Accession no: AB240436.1	TTCTACCAAGACTTCCCGGAGGACAGCGCATGGGCGCATCCTGTGGATGG
Brevibacterium linens Accession no: CP014869.1	TTCCACCAGCAGCATGAGTCGGACTCGGCGTGGGCACACCCGATCGACGG
***Brevibacterium sp.* YB235** Accession no: CP050153.1	TTCCACCAGCAGCATGAGTCGGACTCGGCGTGGGCACACCCGATCGACGG
***Cryobacterium flavum* strain Hh8** Accession no: SOFD01000024.1	TTCGTGCAGAACTTTCCGGAGGACAGCGCCTGGGCCCACCCGATCGACGG
***Cryobacterium sp.* MLB32 c47** Accession no: JPRS01000047.1	TTCGTGCAGAACTTTCCGGAGGACAGCGCCTGGGCCCACCCGATCGACGG
***Cryobacterium luteum* strain Hh15** Accession no: SOFF01000030.1	TTCGTGCAGAACTTTCCGGAGGACAGCGCCTGGGCCCACCCGATCGACGG
Cryobacterium roopkundense Accession no: JPXF01000060.1	TTCGTGCAGAACTTTCCGGAGGACAGCGCCTGGGCCCACCCGATCGACGG
Kocuria indica Accession no: CP035504.1	TTCCTGCAGGAGCACGAGGAGGACTCGGCGTGGGCGCACCCGATCGACGG
Kocuria flava Accession no: CP013254.1	TTCCTGCAGGAGCACGAGGAGGACTCGGCGTGGGCGCACCCGATCGACGG

Microbacterium azadirachtae Accession no: JYIX 01000018.1	TTCCTCCGCTACGACGAGCTCGACTCGCCCTGGGCGCACCCGGTCGAGGG
Microbacterium oxydans Accession no:CP031422.1	TTCCTCCGCTACGACGAGCTCGACTCGCCCTGGGCGCACCCGGTCGAGGG
Microbacterium trichothecenolyticum Accession no: JYJA01000039.1	TTCCTCCGCTACGACGAGCTCGACTCGCCCTGGGCGCACCCGGTCGAGGG
Microbacterium foliorum Accession no: JYIU01000019.1	TTCCTCCGCTACGACGAGCTCGACTCGCCCTGGGCGCACCCGGTCGAGGG
Microbacterium mangrove Accession no: JTDK01000001.1	TTCCTCCGCTACGACGAGCTCGACTCGCCCTGGGCGCACCCGGTCGAGGG
Nocardiopsis sp. **NRRL B-16309** Accession no: LGEC01000081.1	TTCGTCCAGGAGCACGAGACCGACCACGCCTGGGCGCACCCGGTGGACGG
Paenarthrobacter aurescens Accession no: CP000474.1	TTTGTGCAGGACTTCCCGGAGGACAGCGCTTGGGCCCATCCCGTCGATGG
PseudArthrobacter sp. Accession no: CP041198.1	TTCGTCCAGGAGTTCCCGGAGGACAGTGCGTGGGCGCACCCGGTGGACGG
Pseudarthrobacter chlorophenolicus A6 Accession no: CP001341.1	TTCGTCCAGGAGTTCCCGGAGGACAGTGCGTGGGCGCACCCGGTGGACGG
Rhodococcus jostii **RHA1** Accession no: CP000431.1	TTCCGCCAGGACCACCCGAAGGACCATGCCTGGGCCCACCCGATCGACGG
Rhodococcus opacus **1CP** Accession no: CP009111.1	TTCCGCCAGGACCACCCGAAGGACCATGCCTGGGCCCACCCGATCGACGG
Rhodococcus ruber Accession no: CCSD01000085.1	TTCCGCCAGGACCACCCGAAGGACCATGCCTGGGCCCACCCGATCGACGG
Rhodococcus wratislaviensis Accession no:BHYM01000005.1	TTCCGCCAGGACCACCCGAAGGACCATGCCTGGGCCCACCCGATCGACGG
Rhodococcus sp.SC4 **SC4** Accession no: LSBM01000283.1	TTCCGCCAGGACCACCCGAAGGACCATGCCTGGGCCCACCCGATCGACGG
Saccharothrix espanaensis Accession no: He804045.1	TTCCTGCGCGACCACGTGGACGACAGCCCGTGGGCACACCCGGTCGAGGG
Sinomonas atrocyanea Accession no: CP014518.1	TTCGTCCAGGAGCACGCATCCGACAGCCCGTGGGCGCACCCCGTGGACGG
Streptomyces ossamyceticus Accession no: NZ_RJKY01000001.1	TTCGTCCAGAAGAGCCCGGAGGACTACGCATGGGGATGTCCCGTCGACGG
Streptomyces fulvoviolaceus Accession no: NZ_JOEY01000080.1	TTCGTCCAGAAGAGCCCGGAGGACTACGCATGGGGATGTCCCGTCGACGG
Streptomyces torulosus Accession no: NZ_LIRK01000041.1	TTCGTCCAGAAGAGCCCGGAGGACTACGCATGGGGATGTCCCGTCGACGG
Streptomyces xylophagus Accession no: NZ_JNW001000006.1	TTCGTCCAGAAGAGCCCGGAGGACTACGCATGGGGATGTCCCGTCGACGG
	** * * *** * **** ** * ** **
Amycolatopsis thermoflava **N1165** Accessionn no: NZ_KI421511.1	GCTCGTCGGGTTCGTCGACGTCCTGGCCCGCGAGGTCACCCGGGTGATCG
Amycolatopsis japonica **strain MG417-CF17** Accession no: CP008953.1	GCTCGTCGGGTTCGTCGACGTCCTGGCCCGCGAGGTCACCCGGGTGATCG
Arthrobacter alpinus **ERGS4** Accession no: CP013200.1	GCTGGTTGCCTATGTGGATGTCACCAACAAGAGCGTCGATGCGGTTCTGG
Arthrobacter sp. 31Y **K253DRAFT** Accession no: JAFW01000002.1	GCTGGTTGCCTATGTGGATGTCACCAACAAGAGCGTCGATGCGGTTCTGG
Arthrobacter sp. M2012083 Accession no: NZ_AKKK01000062.1	GCTGGTTGCCTATGTGGATGTCACCAACAAGAGCGTCGATGCGGTTCTGG
Arthrobacter sp. **EpRS71** Accession no: LNUV01000006.1	GCTGGTTGCCTATGTGGATGTCACCAACAAGAGCGTCGATGCGGTTCTGG
Arthrobacter crystallopoietes Accession no: AB240436.1	GCTGGTTGCCTATGTGGATGTCACCAACAAGAGCGTCGATGCGGTTCTGG
Brevibacterium linens Accession no: CP014869.1	ACTCGTCGCCTACGTTGACGTCACGAACGAGGCCGTCGATCAGATTCTCG
Brevibacterium sp. **YB235** Accession no: CP050153.1	ACTCGTCGCCTACGTTGACGTCACGAACGAGGCCGTCGATCAGATTCTCG
Cryobacterium flavum **strain Hh8** Accession no: SOFD01000024.1	CCTCGTCGCCTACGTCGACATCGTCGCGAAGACCGTGGACCAGGTGCTCG
Cryobacterium sp. **MLB32 c47** Accession no: JPRS01000047.1	CCTCGTCGCCTACGTCGACATCGTCGCGAAGACCGTGGACCAGGTGCTCG
Cryobacterium luteum **strain Hh15** Accession no: SOFF01000030.1	CCTCGTCGCCTACGTCGACATCGTCGCGAAGACCGTGGACCAGGTGCTCG
Cryobacterium roopkundense Accession no: JPXF01000060.1	CCTCGTCGCCTACGTCGACATCGTCGCGAAGACCGTGGACCAGGTGCTCG
Kocuria indica Accession no: CP035504.1	GCTCGTGGCCTACGTGGACGTGACCAACCAGGCCGTGGACCAGGTGCTGG
Kocuria flava Accession no: CP013254.1	GCTCGTGGCCTACGTGGACGTGACCAACCAGGCCGTGGACCAGGTGCTGG
Microbacterium azadirachtae Accession no: JYIX 01000018.1	ACTCATCGTGCACATCGACCTCACGGCGGACAGCGTGATCCGCGTCGAGG
Microbacterium oxydans Accession no:CP031422.1	ACTCATCGTGCACATCGACCTCACGGCGGACAGCGTGATCCGCGTCGAGG
Microbacterium trichothecenolyticum Accession no: JYJA01000039.1	ACTCATCGTGCACATCGACCTCACGGCGGACAGCGTGATCCGCGTCGAGG
Microbacterium foliorum Accession no: JYIU01000019.1	ACTCATCGTGCACATCGACCTCACGGCGGACAGCGTGATCCGCGTCGAGG
Microbacterium mangrove Accession no: JTDK01000001.1	ACTCATCGTGCACATCGACCTCACGGCGGACAGCGTGATCCGCGTCGAGG
Nocardiopsis sp. **NRRL B-16309** Accession no: LGEC01000081.1	ACTCGTGGCCTACGTCGACGTCGTCGGCAGGACCGTCGACCGGGTCATCG
Paenarthrobacter aurescens Accession no: CP000474.1	GCTGGTGGCGTACGTGGACGTGGTCAACAAGGAAGTCACCCAGGTCATCG
PseudArthrobacter sp. Accession no: CP041198.1	CCTGGTGGCCTACGTGGACGTGGTTCGCCAGGAGGTCACCCAGGTGATCG
Pseudarthrobacter chlorophenolicus A6 Accession no: CP001341.1	CCTGGTGGCCTACGTGGACGTGGTTCGCCAGGAGGTCACCCAGGTGATCG
Rhodococcus jostii **RHA1** Accession no: CP000431.1	GCTCGTCGGATTCGTCGACGTCATGAACCGGACCGTCACCGACGTTCTCG
Rhodococcus opacus **1CP** Accession no: CP009111.1	GCTCGTCGGATTCGTCGACGTCATGAACCGGACCGTCACCGACGTTCTCG
Rhodococcus ruber Accession no: CCSD01000085.1	GCTCGTCGGATTCGTCGACGTCATGAACCGGACCGTCACCGACGTTCTCG
Rhodococcus wratislaviensis Accession no:BHYM01000005.1	GCTCGTCGGATTCGTCGACGTCATGAACCGGACCGTCACCGACGTTCTCG
Rhodococcus sp.SC4 **SC4** Accession no: LSBM01000283.1	GCTCGTCGGATTCGTCGACGTCATGAACCGGACCGTCACCGACGTTCTCG
Saccharothrix espanaensis Accession no: He804045.1	TCTGCTCTGCGAGGTGGACCTGATCGCCAACGAGGTGCTGGCGGTGACCG
Sinomonas atrocyanea Accession no: CP014518.1	GCTCGTGGCCTACGTCGACGTCGTGAACCGCACCGTGGACCAGGTCCTCG
Streptomyces ossamyceticus Accession no: NZ_RJKY01000001.1	TCTCGTCGTCTACGTCGACGTGCTGAACAGGACCGTCGACCGGATCATCG
Streptomyces fulvoviolaceus Accession no: NZ_JOEY01000080.1	TCTCGTCGTCTACGTCGACGTGCTGAACAGGACCGTCGACCGGATCATCG
Streptomyces torulosus Accession no: NZ_LIRK01000041.1	TCTCGTCGTCTACGTCGACGTGCTGAACAGGACCGTCGACCGGATCATCG

Streptomyces xylophagus Accession no: NZ_JNW001000006.1	TCTCGTCGTCTACGTCGACGTGCTGAACAGGACCGTCGACCGGATCATCG
	** * * ** * ** * *
***Amycolatopsis thermoflava* N1165** Accessionn no: NZ_KI421511.1	ACCTCGGCCCGGTGCCGATCCCGGCCGAACCGGGCAACTTCGACGACCCC
***Amycolatopsis japonica* strain MG417-CF17** Accession no: CP008953.1	ACCTCGGCCCGGTGCCGATCCCGGCCGAACCGGGCAACTTCGACGACCCC
***Arthrobacter alpinus* ERGS4** Accession no: CP013200.1	ATTTTGGTGTGGTCCCGGTTCCGGCCGAGCATGGCAATTACACGGATCCT
Arthrobacter sp. 31Y K253DRAFT Accession no: JAFW01000002.1	ATTTTGGTGTGGTCCCGGTTCCGGCCGAGCATGGCAATTACACGGATCCT
Arthrobacter sp. M2012083 Accession no: NZ_AKKK01000062.1	ATTTTGGTGTGGTCCCGGTTCCGGCCGAGCATGGCAATTACACGGATCCT
***Arthrobacter sp.* EpRS71** Accession no: LNUV01000006.1	ATTTTGGTGTGGTCCCGGTTCCGGCCGAGCATGGCAATTACACGGATCCT
Arthrobacter crystallopoietes Accession no: AB240436.1	ATTTTGGTGTGGTCCCGGTTCCGGCCGAGCATGGCAATTACACGGATCCT
Brevibacterium linens Accession no: CP014869.1	ACCTCGAAAACGTTCCGGTGCCGTCCGAGCACGGGAACTACACGGATCCG
***Brevibacterium sp.* YB235** Accession no: CP050153.1	ACCTCGAAAACGTTCCGGTGCCGTCCGAGCACGGGAACTACACGGATCCG
***Cryobacterium flavum* strain Hh8** Accession no: SOFD01000024.1	ACTTCGGCGTCGTTCCCATCCCCGCCGAACACGGCAACTTCACCGATCCC
***Cryobacterium sp.* MLB32 c47** Accession no: JPRS01000047.1	ACTTCGGCGTCGTTCCCATCCCCGCCGAACACGGCAACTTCACCGATCCC
***Cryobacterium luteum* strain Hh15** Accession no: SOFF01000030.1	ACTTCGGCGTCGTTCCCATCCCCGCCGAACACGGCAACTTCACCGATCCC
Cryobacterium roopkundense Accession no: JPXF01000060.1	ACTTCGGCGTCGTTCCCATCCCCGCCGAACACGGCAACTTCACCGATCCC
Kocuria indica Accession no: CP035504.1	ACCTGGAGGTGGTCCCGGTGCCGCAGGAGCACGGCAACTACACGGATCCC
Kocuria flava Accession no: CP013254.1	ACCTGGAGGTGGTCCCGGTGCCGCAGGAGCACGGCAACTACACGGATCCC
Microbacterium azadirachtae Accession no: JYIX 01000018.1	ACATCGGCGACGTCCCGGTACCCGCCGGCCATGGCAACTACTATCCCGAG
Microbacterium oxydans Accession no:CP031422.1	ACATCGGCGACGTCCCGGTACCCGCCGGCCATGGCAACTACTATCCCGAG
Microbacterium trichothecenolyticum Accession no: JYJA01000039.1	ACATCGGCGACGTCCCGGTACCCGCCGGCCATGGCAACTACTATCCCGAG
Microbacterium foliorum Accession no: JYIU01000019.1	ACATCGGCGACGTCCCGGTACCCGCCGGCCATGGCAACTACTATCCCGAG
Microbacterium mangrove Accession no: JTDK01000001.1	ACATCGGCGACGTCCCGGTACCCGCCGGCCATGGCAACTACTATCCCGAG
***Nocardiopsis sp.* NRRL B-16309** Accession no: LGEC01000081.1	ACTTCGGGCCCGTGCCGATCCCGACCGAGTCCGGCAACTACCCCGATCCC
Paenarthrobacter aurescens Accession no: CP000474.1	ATCTGGGCGTCATGCCAATCCCGGCTGAGCACGGAAACTACACGGATCCT
PseudArthrobacter sp. Accession no: CP041198.1	ACCTCGGTGCCATGCCCATCCCGGCCGAGCACGGCAACTACACAGACCCT
***Pseudarthrobacter chlorophenolicus* A6** Accession no: CP001341.1	ACCTCGGTGCCATGCCCATCCCGGCCGAGCACGGCAACTACACAGACCCT
***Rhodococcus jostii* RHA1** Accession no: CP000431.1	ACCTCGGCGCCGTCCCGGTCCCCGAGGAATCGGGCAACTTCGACGACCTC
***Rhodococcus opacus* 1CP** Accession no: CP009111.1	ACCTCGGCGCCGTCCCGGTCCCCGAGGAATCGGGCAACTTCGACGACCTC
Rhodococcus ruber Accession no: CCSD01000085.1	ACCTCGGCGCCGTCCCGGTCCCCGAGGAATCGGGCAACTTCGACGACCTC
Rhodococcus wratislaviensis Accession no:BHYM01000005.1	ACCTCGGCGCCGTCCCGGTCCCCGAGGAATCGGGCAACTTCGACGACCTC
***Rhodococcus sp.*SC4 SC4** Accession no: LSBM01000283.1	ACCTCGGCGCCGTCCCGGTCCCCGAGGAATCGGGCAACTTCGACGACCTC
Saccharothrix espanaensis Accession no: He804045.1	ACGAGGGCGACGTCCCCACGCCGGTGGAGCACGGCAACTACACCGGCGA-
Sinomonas atrocyanea Accession no: CP014518.1	ACTTCGGCCCGGTCCCGGTGCCCGCGGAGCACGGCAACTTCACCGATCCG
Streptomyces ossamyceticus Accession no: NZ_RJKY01000001.1	ACCTCGACCCGGTGCCGCTGCCCGACGTGTCCGTCGACTTCGGCGACCCG
Streptomyces fulvoviolaceus Accession no: NZ_JOEY01000080.1	ACCTCGACCCGGTGCCGCTGCCCGACGTGTCCGTCGACTTCGGCGACCCG
Streptomyces torulosus Accession no: NZ_LIRK01000041.1	ACCTCGACCCGGTGCCGCTGCCCGACGTGTCCGTCGACTTCGGCGACCCG
Streptomyces xylophagus Accession no: NZ_JNW001000006.1	ACCTCGACCCGGTGCCGCTGCCCGACGTGTCCGTCGACTTCGGCGACCCG
	* * * ** ** * * * * *
***Amycolatopsis thermoflava* N1165** Accessionn no: NZ_KI421511.1	GCCGTCACCGGTCCACTTCGGACCTCCCAGAAGCCACTGGAGATCACGCA
***Amycolatopsis japonica* strain MG417-CF17** Accession no: CP008953.1	GCCGTCACCGGTCCACTTCGGACCTCCCAGAAGCCACTGGAGATCACGCA
***Arthrobacter alpinus* ERGS4** Accession no: CP013200.1	GAACTGACGGGCCCTACGCGGACAACCCAGAAGCCCATCAACATCACCCA
Arthrobacter sp. 31Y K253DRAFT Accession no: JAFW01000002.1	GAACTGACGGGCCCTACGCGGACAACCCAGAAGCCCATCAACATCACCCA
Arthrobacter sp. M2012083 Accession no: NZ_AKKK01000062.1	GAACTGACGGGCCCTACGCGGACAACCCAGAAGCCCATCAACATCACCCA
***Arthrobacter sp.* EpRS71** Accession no: LNUV01000006.1	GAACTGACGGGCCCTACGCGGACAACCCAGAAGCCCATCAACATCACCCA
Arthrobacter crystallopoietes Accession no: AB240436.1	GAACTGACGGGCCCTACGCGGACAACCCAGAAGCCCATCAACATCACCCA
Brevibacterium linens Accession no: CP014869.1	GAGCTGACCGGACCCGTGCGAACCACACAGAAGCCGATCTCGATCACGCA
***Brevibacterium sp.* YB235** Accession no: CP050153.1	GAGCTGACCGGACCCGTGCGAACCACACAGAAGCCGATCTCGATCACGCA
***Cryobacterium flavum* strain Hh8** Accession no: SOFD01000024.1	GCCCTCACCGGCCCGCTGCGCGAAACGCTCAAGCCGATCAGTATCACCCA
***Cryobacterium sp.* MLB32 c47** Accession no: JPRS01000047.1	GCCCTCACCGGCCCGCTGCGCGAAACGCTCAAGCCGATCAGTATCACCCA
***Cryobacterium luteum* strain Hh15** Accession no: SOFF01000030.1	GCCCTCACCGGCCCGCTGCGCGAAACGCTCAAGCCGATCAGTATCACCCA
Cryobacterium roopkundense Accession no: JPXF01000060.1	GCCCTCACCGGCCCGCTGCGCGAAACGCTCAAGCCGATCAGTATCACCCA
Kocuria indica Accession no: CP035504.1	GAGCTCACCGGACCCACGCGCACCACCCAGAAGCCGATCGAGATCACCCA
Kocuria flava Accession no: CP013254.1	GAGCTCACCGGACCCACGCGCACCACCCAGAAGCCGATCGAGATCACCCA
Microbacterium azadirachtae Accession no: JYIX 01000018.1	GTGCAGGGC---GAGGCGCGCACGACCCTCAAGCCGATCGAGATCGTCCA

Microbacterium oxydans Accession no:CP031422.1	GTGCAGGGC---GAGGCGCGCACGACCCTCAAGCCGATCGAGATCGTCCA
Microbacterium trichothecenolyticum Accession no: JYJA01000039.1	GTGCAGGGC---GAGGCGCGCACGACCCTCAAGCCGATCGAGATCGTCCA
Microbacterium foliorum Accession no: JYIU01000019.1	GTGCAGGGC---GAGGCGCGCACGACCCTCAAGCCGATCGAGATCGTCCA
Microbacterium mangrove Accession no: JTDK01000001.1	GTGCAGGGC---GAGGCGCGCACGACCCTCAAGCCGATCGAGATCGTCCA
Nocardiopsis sp. **NRRL B-16309** Accession no: LGEC01000081.1	GAGGTGAGCGGGCCGCCGCGCACCACCCAGAGGCCCATCGAGATCACTCA
Paenarthrobacter aurescens Accession no: CP000474.1	GAACTGACGGGCCCGCTCCGGGAAACGCAGAAGCCCATCAGCATCACGCA
PseudArthrobacter sp. Accession no: CP041198.1	GAACTGACCGGACCCATCCGCGAAACCCAGAAGCCCATCAGCATTACCCA
Pseudarthrobacter chlorophenolicus **A6** Accession no: CP001341.1	GAACTGACCGGACCCATCCGCGAAACCCAGAAGCCCATCAGCATTACCCA
Rhodococcus jostii **RHA1** Accession no: CP000431.1	GCCGTCACCGGCCCCCTGCGCACCACCCAGAAGCCCATCGAGATCACCCA
Rhodococcus opacus **1CP** Accession no: CP009111.1	GCCGTCACCGGCCCCCTGCGCACCACCCAGAAGCCCATCGAGATCACCCA
Rhodococcus ruber Accession no: CCSD01000085.1	GCCGTCACCGGCCCCCTGCGCACCACCCAGAAGCCCATCGAGATCACCCA
Rhodococcus wratislaviensis Accession no:BHYM01000005.1	GCCGTCACCGGCCCCCTGCGCACCACCCAGAAGCCCATCGAGATCACCCA
***Rhodococcus sp.*SC4 SC4** Accession no: LSBM01000283.1	GCCGTCACCGGCCCCCTGCGCACCACCCAGAAGCCCATCGAGATCACCCA
Saccharothrix espanaensis Accession no: He804045.1	--------G---CCGGCCCGCACCACGCTCAAGCCGATCTCCATCACCCA
Sinomonas atrocyanea Accession no: CP014518.1	GAGCTGACCGGGCCGGTCCGCGGCACCCAGAAGCCGATCGAGATCACCCA
Streptomyces ossamyceticus Accession no: NZ_RJKY01000001.1	GCGGTCACCGG-CCGC--CGCACCAGCCAGAAGCCCATCGAGATCACCCA
Streptomyces fulvoviolaceus Accession no: NZ_JOEY01000080.1	GCGGTCACCGG-CCGC--CGCACCAGCCAGAAGCCCATCGAGATCACCCA
Streptomyces torulosus Accession no: NZ_LIRK01000041.1	GCGGTCACCGG-CCGC--CGCACCAGCCAGAAGCCCATCGAGATCACCCA
Streptomyces xylophagus Accession no: NZ_JNW001000006.1	GCGGTCACCGG-CCGC--CGCACCAGCCAGAAGCCCATCGAGATCACCCA
	** * * *** * ** **
Amycolatopsis thermoflava **N1165** Accessionn no: NZ_KI421511.1	ACCGGACGGTCCCAGCTTCACCCTC---GACGGGCACCTGCTGCGGTGGG
Amycolatopsis japonica **strain MG417-CF17** Accession no: CP008953.1	ACCGGACGGTCCCAGCTTCACCCTC---GACGGGCACCTGCTGCGGTGGG
Arthrobacter alpinus **ERGS4** Accession no: CP013200.1	ACCCGAAGGTCCTAGCTTCTCCGTGACCGCCGGCAATCACATTGAATGGG
Arthrobacter sp. 31Y K253DRAFT Accession no: JAFW01000002.1	ACCCGAAGGTCCTAGCTTCTCCGTGACCGCCGGCAATCACATTGAATGGG
Arthrobacter sp. M2012083 Accession no: NZ_AKKK01000062.1	ACCCGAAGGTCCTAGCTTCTCCGTGACCGCCGGCAATCACATTGAATGGG
Arthrobacter sp. **EpRS71** Accession no: LNUV01000006.1	ACCCGAAGGTCCTAGCTTCTCCGTGACCGCCGGCAATCACATTGAATGGG
Arthrobacter crystallopoietes Accession no: AB240436.1	ACCCGAAGGTCCTAGCTTCTCCGTGACCGCCGGCAATCACATTGAATGGG
Brevibacterium linens Accession no: CP014869.1	GCCGGATGGCCCGAGCTTCACCGTCACCGAGGGCAACCACATCGAATGGG
Brevibacterium sp. **YB235** Accession no: CP050153.1	GCCGGATGGCCCGAGCTTCACCGTCACCGAGGGCAACCACATCGAATGGG
Cryobacterium flavum **strain Hh8** Accession no: SOFD01000024.1	GCCCGATGGCGTGAGCTTTACCGTCACCGGCGGCAACCACATCGAGTGGG
Cryobacterium sp. **MLB32 c47** Accession no: JPRS01000047.1	GCCCGATGGCGTGAGCTTTACCGTCACCGGCGGCAACCACATCGAGTGGG
Cryobacterium luteum **strain Hh15** Accession no: SOFF01000030.1	GCCCGATGGCGTGAGCTTTACCGTCACCGGCGGCAACCACATCGAGTGGG
Cryobacterium roopkundense Accession no: JPXF01000060.1	GCCCGATGGCGTGAGCTTTACCGTCACCGGCGGCAACCACATCGAGTGGG
Kocuria indica Accession no: CP035504.1	GCCCGAGGGGCCGTCCTTCACCGTGACCGGCGGCAACCACGTGGAGTGGG
Kocuria flava Accession no: CP013254.1	GCCCGAGGGGCCGTCCTTCACCGTGACCGGCGGCAACCACGTGGAGTGGG
Microbacterium azadirachtae Accession no: JYIX 01000018.1	GCCCGAGGGGCCGAGCTTCTCCGTG---ACCGGATCGCTCGTCGAGTGGG
Microbacterium oxydans Accession no:CP031422.1	GCCCGAGGGGCCGAGCTTCTCCGTG---ACCGGATCGCTCGTCGAGTGGG
Microbacterium trichothecenolyticum Accession no: JYJA01000039.1	GCCCGAGGGGCCGAGCTTCTCCGTG---ACCGGATCGCTCGTCGAGTGGG
Microbacterium foliorum Accession no: JYIU01000019.1	GCCCGAGGGGCCGAGCTTCTCCGTG---ACCGGATCGCTCGTCGAGTGGG
Microbacterium mangrove Accession no: JTDK01000001.1	GCCCGAGGGGCCGAGCTTCTCCGTG---ACCGGATCGCTCGTCGAGTGGG
Nocardiopsis sp. **NRRL B-16309** Accession no: LGEC01000081.1	GCCCGAGGGGCCGAGCTTCACCCTC---GACGGCAACGTGCTGAGCTGGG
Paenarthrobacter aurescens Accession no: CP000474.1	GCCGGAAGGCCCCAGCTTCACCGTCACCGGCGGCAACCATGTGGAGTGGG
PseudArthrobacter sp. Accession no: CP041198.1	GCCGGAAGGTCCCAGCTTCACTGTCACCGGCGGGAACCACGTGGAATGGG
Pseudarthrobacter chlorophenolicus **A6** Accession no: CP001341.1	GCCGGAAGGTCCCAGCTTCACTGTCACCGGCGGGAACCACGTGGAATGGG
Rhodococcus jostii **RHA1** Accession no: CP000431.1	GCCCGAGGGACCCAGCTTCGTCGTC---GACGGCAACCGCGTCGACTGGG
Rhodococcus opacus **1CP** Accession no: CP009111.1	GCCCGAGGGACCCAGCTTCGTCGTC---GACGGCAACCGCGTCGACTGGG
Rhodococcus ruber Accession no: CCSD01000085.1	GCCCGAGGGACCCAGCTTCGTCGTC---GACGGCAACCGCGTCGACTGGG
Rhodococcus wratislaviensis Accession no:BHYM01000005.1	GCCCGAGGGACCCAGCTTCGTCGTC---GACGGCAACCGCGTCGACTGGG
***Rhodococcus sp.*SC4 SC4** Accession no: LSBM01000283.1	GCCCGAGGGACCCAGCTTCGTCGTC---GACGGCAACCGCGTCGACTGGG
Saccharothrix espanaensis Accession no: He804045.1	GCCCGAGGGCGTCAGCTTCACCGTC---GAGGGCCACGAGGTGAGCTGGG
Sinomonas atrocyanea Accession no: CP014518.1	GCCCGACGGCCCGAGCTTCACGGTCACCGGCGGCAACCACGTCGAATGGG
Streptomyces ossamyceticus Accession no: NZ_RJKY01000001.1	GCCCGAGGGGCCGAGCTTCACCCTC---GACGGCAACCTCCTGGAGTGGG
Streptomyces fulvoviolaceus Accession no: NZ_JOEY01000080.1	GCCCGAGGGGCCGAGCTTCACCCTC---GACGGCAACCTCCTGGAGTGGG
Streptomyces torulosus Accession no: NZ_LIRK01000041.1	GCCCGAGGGGCCGAGCTTCACCCTC---GACGGCAACCTCCTGGAGTGGG
Streptomyces xylophagus Accession no: NZ_JNW001000006.1	GCCCGAGGGGCCGAGCTTCACCCTC---GACGGCAACCTCCTGGAGTGGG

	** ** ** *** * ** * ****
Amycolatopsis thermoflava **N1165** Accessionn no: NZ_KI421511.1	AGAACTGGTCGCTGCGGATCGGCTTCGACGCCCGCGAAGGCCTTGTCCTG
Amycolatopsis japonica **strain MG417-CF17** Accession no: CP008953.1	AGAACTGGTCGCTGCGGATCGGCTTCGACGCCCGCGAAGGCCTTGTCCTG
Arthrobacter alpinus **ERGS4** Accession no: CP013200.1	AGAACTGGAGCCTGGACATCGGCTTCGACGTCCGTGAAGGCGTTGTCCTG
Arthrobacter sp. 31Y K253DRAFT Accession no: JAFW01000002.1	AGAACTGGAGCCTGGACATCGGCTTCGACGTCCGTGAAGGCGTTGTCCTG
Arthrobacter sp. M2012083 Accession no: NZ_AKKK01000062.1	AGAACTGGAGCCTGGACATCGGCTTCGACGTCCGTGAAGGCGTTGTCCTG
Arthrobacter sp. **EpRS71** Accession no: LNUV01000006.1	AGAACTGGAGCCTGGACATCGGCTTCGACGTCCGTGAAGGCGTTGTCCTG
Arthrobacter crystallopoietes Accession no: AB240436.1	AGAACTGGAGCCTGGACATCGGCTTCGACGTCCGTGAAGGCGTTGTCCTG
Brevibacterium linens Accession no: CP014869.1	AGAAGTGGTCGTTCGATGTCGGCTTCGATATGCGTGAGGGTCTCGTCCTC
Brevibacterium sp. **YB235** Accession no: CP050153.1	AGAAGTGGTCGTTCGATGTCGGCTTCGATATGCGTGAGGGTCTCGTCCTC
Cryobacterium flavum **strain Hh8** Accession no: SOFD01000024.1	AGAAATGGAGCCTCGACGTGGGTTTCGACGTGCGCGAGGGCGTCGTGCTG
Cryobacterium sp. **MLB32 c47** Accession no: JPRS01000047.1	AGAAATGGAGCCTCGACGTGGGTTTCGACGTGCGCGAGGGCGTCGTGCTG
Cryobacterium luteum **strain Hh15** Accession no: SOFF01000030.1	AGAAATGGAGCCTCGACGTGGGTTTCGACGTGCGCGAGGGCGTCGTGCTG
Cryobacterium roopkundense Accession no: JPXF01000060.1	AGAAATGGAGCCTCGACGTGGGTTTCGACGTGCGCGAGGGCGTCGTGCTG
Kocuria indica Accession no: CP035504.1	AGAAGTGGAGCCTGGACGTGGGCTTCGACATGCGCGAGGGGCTCGTGCTG
Kocuria flava Accession no: CP013254.1	AGAAGTGGAGCCTGGACGTGGGCTTCGACATGCGCGAGGGGCTCGTGCTG
Microbacterium azadirachtae Accession no: JYIX 01000018.1	AGAACTGGTCGATGCGGGTGAGCTTCAACGCGCGCGAGGGCCTCGTGCTG
Microbacterium oxydans Accession no:CP031422.1	AGAACTGGTCGATGCGGGTGAGCTTCAACGCGCGCGAGGGCCTCGTGCTG
Microbacterium trichothecenolyticum Accession no: JYJA01000039.1	AGAACTGGTCGATGCGGGTGAGCTTCAACGCGCGCGAGGGCCTCGTGCTG
Microbacterium foliorum Accession no: JYIU01000019.1	AGAACTGGTCGATGCGGGTGAGCTTCAACGCGCGCGAGGGCCTCGTGCTG
Microbacterium mangrove Accession no: JTDK01000001.1	AGAACTGGTCGATGCGGGTGAGCTTCAACGCGCGCGAGGGCCTCGTGCTG
Nocardiopsis sp. **NRRL B-16309** Accession no: LGEC01000081.1	AGAACTGGTCGCTGCGGATCGGCTTCGACGCCCGCGAGGGGCTGGTGCTG
Paenarthrobacter aurescens Accession no: CP000474.1	AGAAGTGGAGCGTGGACGTTGGCTTCGATGTCCGCGAAGGTGTTGTCCTT
PseudArthrobacter sp. Accession no: CP041198.1	AAAAGTGGAGCCTGGACGTGGGGTTCGATTCCCGCGAAGGCGTGGTCCTG
Pseudarthrobacter chlorophenolicus **A6** Accession no: CP001341.1	AAAAGTGGAGCCTGGACGTGGGGTTCGATTCCCGCGAAGGCGTGGTCCTG
Rhodococcus jostii **RHA1** Accession no: CP000431.1	AGAAGTGGTCGTTCCGCGTCGGTTTCGACGCCCGCGAGGGTCTCGTCCTC
Rhodococcus opacus **1CP** Accession no: CP009111.1	AGAAGTGGTCGTTCCGCGTCGGTTTCGACGCCCGCGAGGGTCTCGTCCTC
Rhodococcus ruber Accession no: CCSD01000085.1	AGAAGTGGTCGTTCCGCGTCGGTTTCGACGCCCGCGAGGGTCTCGTCCTC
Rhodococcus wratislaviensis Accession no:BHYM01000005.1	AGAAGTGGTCGTTCCGCGTCGGTTTCGACGCCCGCGAGGGTCTCGTCCTC
Rhodococcus sp.SC4 **SC4** Accession no: LSBM01000283.1	AGAAGTGGTCGTTCCGCGTCGGTTTCGACGCCCGCGAGGGTCTCGTCCTC
Saccharothrix espanaensis Accession no: He804045.1	AGGGTTGGAAGCTGCGGATCGGGTTCAACGCCCGCGAGGGGCTGACCCTG
Sinomonas atrocyanea Accession no: CP014518.1	AGCGGTGGAGCCTCGACGTCGGCTTCGACGTGCGCGAGGGCCTCGTGCTG
Streptomyces ossamyceticus Accession no: NZ_RJKY01000001.1	AGAACTGGTCCCTGCGGATCGGCTTCGACGCCCGTGAGGGCCTGGTCCTC
Streptomyces fulvoviolaceus Accession no: NZ_JOEY01000080.1	AGAACTGGTCCCTGCGGATCGGCTTCGACGCCCGTGAGGGCCTGGTCCTC
Streptomyces torulosus Accession no: NZ_LIRK01000041.1	AGAACTGGTCCCTGCGGATCGGCTTCGACGCCCGTGAGGGCCTGGTCCTC
Streptomyces xylophagus Accession no: NZ_JNW001000006.1	AGAACTGGTCCCTGCGGATCGGCTTCGACGCCCGTGAGGGCCTGGTCCTC
	* *** * * * *** * ** ** ** * **
Amycolatopsis thermoflava **N1165** Accessionn no: NZ_KI421511.1	CACGAGATCTCGTTCCGCGACGGCGAC------------------CGGGA
Amycolatopsis japonica **strain MG417-CF17** Accession no: CP008953.1	CACGAGATCTCGTTCCGCGACGGCGAC------------------CGGGA
Arthrobacter alpinus **ERGS4** Accession no: CP013200.1	AACAATCTGGCCTTCAATGACC------------------GCGGCCGCAA
Arthrobacter sp. 31Y K253DRAFT Accession no: JAFW01000002.1	AACAATCTGGCCTTCAATGACC------------------GCGGCCGCAA
Arthrobacter sp. M2012083 Accession no: NZ_AKKK01000062.1	AACAATCTGGCCTTCAATGACC------------------GCGGCCGCAA
Arthrobacter sp. **EpRS71** Accession no: LNUV01000006.1	AACAATCTGGCCTTCAATGACC------------------GCGGCCGCAA
Arthrobacter crystallopoietes Accession no: AB240436.1	AACAATCTGGCCTTCAATGACC------------------GCGGCCGCAA
Brevibacterium linens Accession no: CP014869.1	CACAACATCGCCTTCGACGATG------------------GCGAGCGCCG
Brevibacterium sp. **YB235** Accession no: CP050153.1	CACAACATCGCCTTCGACGATG------------------GCGAGCGCCG
Cryobacterium flavum **strain Hh8** Accession no: SOFD01000024.1	CACAACATCGGCTTTCGCGACG------------------GCGCGAAGAC
Cryobacterium sp. **MLB32 c47** Accession no: JPRS01000047.1	CACAACATCGGCTTTCGCGACG------------------GCGCGAAGAC
Cryobacterium luteum **strain Hh15** Accession no: SOFF01000030.1	CACAACATCGGCTTTCGCGACG------------------GCGCGAAGAC
Cryobacterium roopkundense Accession no: JPXF01000060.1	CACAACATCGGCTTTCGCGACG------------------GCGCGAAGAC
Kocuria indica Accession no: CP035504.1	CACAACATCGCGTTCGAGGACC------------------AGGGCCAGAA
Kocuria flava Accession no: CP013254.1	CACAACATCGCGTTCGAGGACC------------------AGGGCCAGAA
Microbacterium azadirachtae Accession no: JYIX 01000018.1	CACGACGTGACCTTCGACGGC-----------------------------
Microbacterium oxydans Accession no:CP031422.1	CACGACGTGACCTTCGACGGC-----------------------------

Microbacterium trichothecenolyticum Accession no: JYJA01000039.1	CACGACGTGACCTTCGACGGC-----------------------------
Microbacterium foliorum Accession no: JYIU01000019.1	CACGACGTGACCTTCGACGGC-----------------------------
Microbacterium mangrove Accession no: JTDK01000001.1	CACGACGTGACCTTCGACGGC-----------------------------
***Nocardiopsis sp.* NRRL B-16309** Accession no: LGEC01000081.1	CACCAGATCGCCTTCGCCGACCGCGACC------------GGGGGCGGGA
Paenarthrobacter aurescens Accession no: CP000474.1	CATAACCTGGCGTTCCAGGACG------------------GCGGCAAGAA
PseudArthrobacter sp. Accession no: CP041198.1	CACAACATCGCCTTCCAGGACA------------------GTGACCGGAA
Pseudarthrobacter chlorophenolicus A6 Accession no: CP001341.1	CACAACATCGCCTTCCAGGACA------------------GTGACCGGAA
***Rhodococcus jostii* RHA1** Accession no: CP000431.1	CATCAGCTCGGATTCGCCGACGGTGAC------------------CGGGT
***Rhodococcus opacus* 1CP** Accession no: CP009111.1	CATCAGCTCGGATTCGCCGACGGTGAC------------------CGGGT
Rhodococcus ruber Accession no: CCSD01000085.1	CATCAGCTCGGATTCGCCGACGGTGAC------------------CGGGT
Rhodococcus wratislaviensis Accession no:BHYM01000005.1	CATCAGCTCGGATTCGCCGACGGTGAC------------------CGGGT
***Rhodococcus sp.*SC4 SC4** Accession no: LSBM01000283.1	CATCAGCTCGGATTCGCCGACGGTGAC------------------CGGGT
Saccharothrix espanaensis Accession no: He804045.1	CACCGGATCTCGTTCGGCGGC-----------------------------
Sinomonas atrocyanea Accession no: CP014518.1	CACAACATCGCCTTCGACGACGCCGCGGCGGGCAACCCGGGCGACCGGAA
Streptomyces ossamyceticus Accession no: NZ_RJKY01000001.1	CACCAGATCGCGTTCCACGACCGCGACC------------AGGGGCGCGA
Streptomyces fulvoviolaceus Accession no: NZ_JOEY01000080.1	CACCAGATCGCGTTCCACGACCGCGACC------------AGGGGCGCGA
Streptomyces torulosus Accession no: NZ_LIRK01000041.1	CACCAGATCGCGTTCCACGACCGCGACC------------AGGGGCGCGA
Streptomyces xylophagus Accession no: NZ_JNW001000006.1	CACCAGATCGCGTTCCACGACCGCGACC------------AGGGGCGCGA
	* * ** *
***Amycolatopsis thermoflava* N1165** Accessionn no: NZ_KI421511.1	CC------------GGCCGATCGTGCACCGGGCCTCGATCTCGGAAATGG
***Amycolatopsis japonica* strain MG417-CF17** Accession no: CP008953.1	CC------------GGCCGATCGTGCACCGGGCCTCGATCTCGGAAATGG
***Arthrobacter alpinus* ERGS4** Accession no: CP013200.1	AC------------GCCCCATCATTAACCGTGCATCCATTGCCGAGATGG
Arthrobacter sp. 31Y K253DRAFT Accession no: JAFW01000002.1	AC------------GCCCCATCATTAACCGTGCATCCATTGCCGAGATGG
Arthrobacter sp. M2012083 Accession no: NZ_AKKK01000062.1	AC------------GCCCCATCATTAACCGTGCATCCATTGCCGAGATGG
***Arthrobacter sp.* EpRS71** Accession no: LNUV01000006.1	AC------------GCCCCATCATTAACCGTGCATCCATTGCCGAGATGG
Arthrobacter crystallopoietes Accession no: AB240436.1	AC------------GCCCCATCATTAACCGTGCATCCATTGCCGAGATGG
Brevibacterium linens Accession no: CP014869.1	TC------------GCACGATCCTCGACCGGGCGGCCATCGCCGAGATGG
***Brevibacterium sp.* YB235** Accession no: CP050153.1	TC------------GCACGATCCTCGACCGGGCGGCCATCGCCGAGATGG
***Cryobacterium flavum* strain Hh8** Accession no: SOFD01000024.1	AC------------GCTCCATCATCCGTCGTGCGTCGATCGCGGAGATGG
***Cryobacterium sp.* MLB32 c47** Accession no: JPRS01000047.1	AC------------GCTCCATCATCCGTCGTGCGTCGATCGCGGAGATGG
***Cryobacterium luteum* strain Hh15** Accession no: SOFF01000030.1	AC------------GCTCCATCATCCGTCGTGCGTCGATCGCGGAGATGG
Cryobacterium roopkundense Accession no: JPXF01000060.1	AC------------GCTCCATCATCCGTCGTGCGTCGATCGCGGAGATGG
Kocuria indica Accession no: CP035504.1	GC------------GCCGGATCCTGGACCGCGCCGCCATCGCGGAGATGA
Kocuria flava Accession no: CP013254.1	GC------------GCCGGATCCTGGACCGCGCCGCCATCGCGGAGATGA
Microbacterium azadirachtae Accession no: JYIX 01000018.1	-C------------GCCCCGTACTCAACCGCGCGAGCGTGCCCGAGATGG
Microbacterium oxydans Accession no:CP031422.1	-C------------GCCCCGTACTCAACCGCGCGAGCGTGCCCGAGATGG
Microbacterium trichothecenolyticum Accession no: JYJA01000039.1	-C------------GCCCCGTACTCAACCGCGCGAGCGTGCCCGAGATGG
Microbacterium foliorum Accession no: JYIU01000019.1	-C------------GCCCCGTACTCAACCGCGCGAGCGTGCCCGAGATGG
Microbacterium mangrove Accession no: JTDK01000001.1	-C------------GCCCCGTACTCAACCGCGCGAGCGTGCCCGAGATGG
***Nocardiopsis sp.* NRRL B-16309** Accession no: LGEC01000081.1	GC------------GCTCGATCGTCCACCGCGCCTCCATCGCCGAGATGG
Paenarthrobacter aurescens Accession no: CP000474.1	GC------------GTCCCATCATCAACCGTGCCTCCATCGCCGAAATGG
PseudArthrobacter sp. Accession no: CP041198.1	GC------------GGCCCATCATCAACCGTGCCTCCATCGCCGAGATGG
Pseudarthrobacter chlorophenolicus A6 Accession no: CP001341.1	GC------------GGCCCATCATCAACCGTGCCTCCATCGCCGAGATGG
***Rhodococcus jostii* RHA1** Accession no: CP000431.1	CC------------GCCCGATCATCCACCGCGCGTCCATCGCCGAGATGG
***Rhodococcus opacus* 1CP** Accession no: CP009111.1	CC------------GCCCGATCATCCACCGCGCGTCCATCGCCGAGATGG
Rhodococcus ruber Accession no: CCSD01000085.1	CC------------GCCCGATCATCCACCGCGCGTCCATCGCCGAGATGG
Rhodococcus wratislaviensis Accession no:BHYM01000005.1	CC------------GCCCGATCATCCACCGCGCGTCCATCGCCGAGATGG
***Rhodococcus sp.*SC4 SC4** Accession no: LSBM01000283.1	CC------------GCCCGATCATCCACCGCGCGTCCATCGCCGAGATGG
Saccharothrix espanaensis Accession no: He804045.1	-G------------ACCCGGTGCTGCACCGGGCGTCGATGGCGGAGATGG
Sinomonas atrocyanea Accession no: CP014518.1	GCCGACCCGCCGCCGCCGCATCCTCGACCGCGCCTCGATCGCCGAGATGG
Streptomyces ossamyceticus Accession no: NZ_RJKY01000001.1	CC------------GTCCCGTCATCCACCGCGCGTCGATCGCCGAGATGG
Streptomyces fulvoviolaceus Accession no: NZ_JOEY01000080.1	CC------------GTCCCGTCATCCACCGCGCGTCGATCGCCGAGATGG
Streptomyces torulosus Accession no: NZ_LIRK01000041.1	CC------------GTCCCGTCATCCACCGCGCGTCGATCGCCGAGATGG
Streptomyces xylophagus Accession no: NZ_JNW001000006.1	CC------------GTCCCGTCATCCACCGCGCGTCGATCGCCGAGATGG
	* * ** ** * * ** ***

***Amycolatopsis thermoflava* N1165** Accessionn no: NZ_KI421511.1	TCGTGCCCTACGCCGACCCCTCCCCCGTCCGCTCGTGGCAGAACTACTTC
Amycolatopsis japonica* strain *MG417-CF17 Accession no: CP008953.1	TCGTGCCCTACGCCGACCCCTCCCCCGTCCGCTCGTGGCAGAACTACTTC
***Arthrobacter alpinus* ERGS4** Accession no: CP013200.1	TGGTCCCTTACGGAGACCCCTCACCGGTGCGGTCCTGGCAAAACTACTTC
Arthrobacter sp. 31Y K253DRAFT Accession no: JAFW01000002.1	TGGTCCCTTACGGAGACCCCTCACCGGTGCGGTCCTGGCAAAACTACTTC
Arthrobacter sp. M2012083 Accession no: NZ_AKKK01000062.1	TGGTCCCTTACGGAGACCCCTCACCGGTGCGGTCCTGGCAAAACTACTTC
***Arthrobacter sp.* EpRS71** Accession no: LNUV01000006.1	TGGTCCCTTACGGAGACCCCTCACCGGTGCGGTCCTGGCAAAACTACTTC
Arthrobacter crystallopoietes Accession no: AB240436.1	TGGTCCCTTACGGAGACCCCTCACCGGTGCGGTCCTGGCAAAACTACTTC
Brevibacterium linens Accession no: CP014869.1	TCGTTCCCTATGGTGACCCGTCACCGGTGCGGTCGTGGCAGAACTATTTC
***Brevibacterium sp.* YB235** Accession no: CP050153.1	TCGTTCCCTATGGTGACCCGTCACCGGTGCGGTCGTGGCAGAACTATTTC
***Cryobacterium flavum* strain Hh8** Accession no: SOFD01000024.1	TCGTGCCGTATGGCGATCCGGCCCCCGTGCGGTCCTGGCAGAACTACTTC
***Cryobacterium sp.* MLB32 c47** Accession no: JPRS01000047.1	TCGTGCCGTATGGCGATCCGGCCCCCGTGCGGTCCTGGCAGAACTACTTC
***Cryobacterium luteum* strain Hh15** Accession no: SOFF01000030.1	TCGTGCCGTATGGCGATCCGGCCCCCGTGCGGTCCTGGCAGAACTACTTC
Cryobacterium roopkundense Accession no: JPXF01000060.1	TCGTGCCGTATGGCGATCCGGCCCCCGTGCGGTCCTGGCAGAACTACTTC
Kocuria indica Accession no: CP035504.1	TGGTGCCCTACGGGGACCCCTCCCCGGTGCGGTCCTGGCAGAACTACTTC
Kocuria flava Accession no: CP013254.1	TGGTGCCCTACGGGGACCCCTCCCCGGTGCGGTCCTGGCAGAACTACTTC
Microbacterium azadirachtae Accession no: JYIX 01000018.1	TCGTCCCCTACGGCGACACCGCGCCGGGGCGCTTCTGGATCAGCTACTTC
Microbacterium oxydans Accession no:CP031422.1	TCGTCCCCTACGGCGACACCGCGCCGGGGCGCTTCTGGATCAGCTACTTC
Microbacterium trichothecenolyticum Accession no: JYJA01000039.1	TCGTCCCCTACGGCGACACCGCGCCGGGGCGCTTCTGGATCAGCTACTTC
Microbacterium foliorum Accession no: JYIU01000019.1	TCGTCCCCTACGGCGACACCGCGCCGGGGCGCTTCTGGATCAGCTACTTC
Microbacterium mangrove Accession no: JTDK01000001.1	TCGTCCCCTACGGCGACACCGCGCCGGGGCGCTTCTGGATCAGCTACTTC
***Nocardiopsis sp.* NRRL B-16309** Accession no: LGEC01000081.1	TCGTCCCCTACGCCGACCCCTCGCCCGTGCGGTCCTGGCAGAACTACTTC
Paenarthrobacter aurescens Accession no: CP000474.1	TTGTTCCGTACGGTGATCCCTCGCCCATTCGGTCATGGCAGAACTACTTC
PseudArthrobacter sp. Accession no: CP041198.1	TGGTGCCCTACGGCGACCCGTCACCCATCAGGTCCTGGCAGAACTACTTC
Pseudarthrobacter chlorophenolicus A6 Accession no: CP001341.1	TGGTGCCCTACGGCGACCCGTCACCCATCAGGTCCTGGCAGAACTACTTC
***Rhodococcus jostii* RHA1** Accession no: CP000431.1	TGGTGCCCTACGGCGATCCCTCGCCGGTCCGGTCGTGGCAGAACTACTTC
***Rhodococcus opacus* 1CP** Accession no: CP009111.1	TGGTGCCCTACGGCGATCCCTCGCCGGTCCGGTCGTGGCAGAACTACTTC
Rhodococcus ruber Accession no: CCSD01000085.1	TGGTGCCCTACGGCGATCCCTCGCCGGTCCGGTCGTGGCAGAACTACTTC
Rhodococcus wratislaviensis Accession no:BHYM01000005.1	TGGTGCCCTACGGCGATCCCTCGCCGGTCCGGTCGTGGCAGAACTACTTC
***Rhodococcus sp.*SC4 SC4** Accession no: LSBM01000283.1	TGGTGCCCTACGGCGATCCCTCGCCGGTCCGGTCGTGGCAGAACTACTTC
Saccharothrix espanaensis Accession no: He804045.1	TGGTGCCCTACGGCGACCCCGCGCCGTGGCGGAACTGGATCAGCTACTTC
Sinomonas atrocyanea Accession no: CP014518.1	TCGTTCCCTACGGCGACCCGTCCCCGGTGCGGTCGTGGCAGAACTACTTC
Streptomyces ossamyceticus Accession no: NZ_RJKY01000001.1	TCGTCCCCTACGCCGACCCGTCGTCGGTGCGCTCCTGGCAGAACTACTTC
Streptomyces fulvoviolaceus Accession no: NZ_JOEY01000080.1	TCGTCCCCTACGCCGACCCGTCGTCGGTGCGCTCCTGGCAGAACTACTTC
Streptomyces torulosus Accession no: NZ_LIRK01000041.1	TCGTCCCCTACGCCGACCCGTCGTCGGTGCGCTCCTGGCAGAACTACTTC
Streptomyces xylophagus Accession no: NZ_JNW001000006.1	TCGTCCCCTACGCCGACCCGTCGTCGGTGCGCTCCTGGCAGAACTACTTC
	* ** ** ** * ** * * * * *** * *** ***
***Amycolatopsis thermoflava* N1165** Accessionn no: NZ_KI421511.1	ACACCGGCGAGTACCTCATCGGCCGCTACGCCAACGCGCTCGAACTCGG
Amycolatopsis japonica* strain *MG417-CF17 Accession no: CP008953.1	GACACCGGCGAGTACCTCATCGGCCGCTACGCCAACGCGCTCGAACTCGG
***Arthrobacter alpinus* ERGS4** Accession no: CP013200.1	GATACAGGCGAATACCTGGTGGGCCAGTACGCCAATTCTCTGGAGCTGGG
Arthrobacter sp. 31Y K253DRAFT Accession no: JAFW01000002.1	GATACAGGCGAATACCTGGTGGGCCAGTACGCCAATTCTCTGGAGCTGGG
Arthrobacter sp. M2012083 Accession no: NZ_AKKK01000062.1	GATACAGGCGAATACCTGGTGGGCCAGTACGCCAATTCTCTGGAGCTGGG
***Arthrobacter sp.* EpRS71** Accession no: LNUV01000006.1	GATACAGGCGAATACCTGGTGGGCCAGTACGCCAATTCTCTGGAGCTGGG
Arthrobacter crystallopoietes Accession no: AB240436.1	GATACAGGCGAATACCTGGTGGGCCAGTACGCCAATTCTCTGGAGCTGGG
Brevibacterium linens Accession no: CP014869.1	GACACCGGCGAATACCTCGTCGGGCAATGGGCGAACTCGCTTGAGCTCGG
***Brevibacterium sp.* YB235** Accession no: CP050153.1	GACACCGGCGAATACCTCGTCGGGCAATGGGCGAACTCGCTTGAGCTCGG
***Cryobacterium flavum* strain Hh8** Accession no: SOFD01000024.1	GACACCGGCGAATACCTCGTCGGCCAGTACGCCAACTCGCTCGAACTCGG
***Cryobacterium sp.* MLB32 c47** Accession no: JPRS01000047.1	GACACCGGCGAATACCTCGTCGGCCAGTACGCCAACTCGCTCGAACTCGG
***Cryobacterium luteum* strain Hh15** Accession no: SOFF01000030.1	GACACCGGCGAATACCTCGTCGGCCAGTACGCCAACTCGCTCGAACTCGG
Cryobacterium roopkundense Accession no: JPXF01000060.1	GACACCGGCGAATACCTCGTCGGCCAGTACGCCAACTCGCTCGAACTCGG
Kocuria indica Accession no: CP035504.1	GACACCGGCGAGTACCTGGTGGGGCAGTGGGCCAACTCCCTGGAGCTCGG
Kocuria flava Accession no: CP013254.1	GACACCGGCGAGTACCTGGTGGGGCAGTGGGCCAACTCCCTGGAGCTCGG
Microbacterium azadirachtae Accession no: JYIX 01000018.1	GACGCCGGCGAGTACCTGCTCGGCAAGAACGCGAACCACCTCGAGCTCGG
Microbacterium oxydans Accession no:CP031422.1	GACGCCGGCGAGTACCTGCTCGGCAAGAACGCGAACCACCTCGAGCTCGG
Microbacterium trichothecenolyticum Accession no: JYJA01000039.1	GACGCCGGCGAGTACCTGCTCGGCAAGAACGCGAACCACCTCGAGCTCGG

Microbacterium foliorum Accession no: JYIU01000019.1	GACGCCGGCGAGTACCTGCTCGGCAAGAACGCGAACCACCTCGAGCTCGG
Microbacterium mangrove Accession no: JTDK01000001.1	GACGCCGGCGAGTACCTGCTCGGCAAGAACGCGAACCACCTCGAGCTCGG
***Nocardiopsis sp.* NRRL B-16309** Accession no: LGEC01000081.1	GACACCGGCGAGTACATGGTCGGCCGCTACGCCAACGCCCTCCGGCTCGG
Paenarthrobacter aurescens Accession no: CP000474.1	GACACCGGTGAATACCTGGTGGGTCAGTACGCGAACTCCTTGGAGCTGGG
PseudArthrobacter sp. Accession no: CP041198.1	GACACCGGCGAATACCTGGTGGGCCAGTACGCCAACTCCCTCGAACTCGG
Pseudarthrobacter chlorophenolicus A6 Accession no: CP001341.1	GACACCGGCGAATACCTGGTGGGCCAGTACGCCAACTCCCTCGAACTCGG
***Rhodococcus jostii* RHA1** Accession no: CP000431.1	GACACCGGCGAGTACATGGTGGGCCGCTACGCGAACGCCCTCGAACTCGG
***Rhodococcus opacus* 1CP** Accession no: CP009111.1	GACACCGGCGAGTACATGGTGGGCCGCTACGCGAACGCCCTCGAACTCGG
Rhodococcus ruber Accession no: CCSD01000085.1	GACACCGGCGAGTACATGGTGGGCCGCTACGCGAACGCCCTCGAACTCGG
Rhodococcus wratislaviensis Accession no:BHYM01000005.1	GACACCGGCGAGTACATGGTGGGCCGCTACGCGAACGCCCTCGAACTCGG
***Rhodococcus sp.*SC4 SC4** Accession no: LSBM01000283.1	GACACCGGCGAGTACATGGTGGGCCGCTACGCGAACGCCCTCGAACTCGG
Saccharothrix espanaensis Accession no: He804045.1	GACGCGGGCGAGTACCTGCTGGGCAAGAACGCGAACTCGCTGCGGCTGGG
Sinomonas atrocyanea Accession no: CP014518.1	GACACCGGCGAGTACCTCGTGGGCCAGTTCGCGAACTCGCTCGAGCTCGG
Streptomyces ossamyceticus Accession no: NZ_RJKY01000001.1	GACACCGGCGAGTTCCTGATCGGCCGCATGGCGAACGCCCTCGAACTGGG
Streptomyces fulvoviolaceus Accession no: NZ_JOEY01000080.1	GACACCGGCGAGTTCCTGATCGGCCGCATGGCGAACGCCCTCGAACTGGG
Streptomyces torulosus Accession no: NZ_LIRK01000041.1	GACACCGGCGAGTTCCTGATCGGCCGCATGGCGAACGCCCTCGAACTGGG
Streptomyces xylophagus Accession no: NZ_JNW001000006.1	GACACCGGCGAGTTCCTGATCGGCCGCATGGCGAACGCCCTCGAACTGGG
	** * ** ** * * * * ** ** ** * ** **
***Amycolatopsis thermoflava* N1165** Accessionn no: NZ_KI421511.1	CTGCGACTGCCTCGGTGAGATCACCTACCTGGACGCGGTGATCGCCGACG
Amycolatopsis japonica strain MG417-CF17 Accession no: CP008953.1	CTGCGACTGCCTCGGTGAGATCACCTACCTGGACGCGGTGATCGCCGACG
***Arthrobacter alpinus* ERGS4** Accession no: CP013200.1	CTGCGACTGCCTGGGTGAAATCACCTACCTGAGCCCCGTCGTCGCAGACG
Arthrobacter sp. 31Y K253DRAFT Accession no: JAFW01000002.1	CTGCGACTGCCTGGGTGAAATCACCTACCTGAGCCCCGTCGTCGCAGACG
Arthrobacter sp. M2012083 Accession no: NZ_AKKK01000062.1	CTGCGACTGCCTGGGTGAAATCACCTACCTGAGCCCCGTCGTCGCAGACG
***Arthrobacter sp.* EpRS71** Accession no: LNUV01000006.1	CTGCGACTGCCTGGGTGAAATCACCTACCTGAGCCCCGTCGTCGCAGACG
Arthrobacter crystallopoietes Accession no: AB240436.1	CTGCGACTGCCTGGGTGAAATCACCTACCTGAGCCCCGTCGTCGCAGACG
Brevibacterium linens Accession no: CP014869.1	GTGTGACTGCCTCGGCGATATCACCTATCTGTCGCCGTGGGTGGCGAACA
***Brevibacterium sp.* YB235** Accession no: CP050153.1	GTGTGACTGCCTCGGCGATATCACCTATCTGTCGCCGTGGGTGGCGAACA
***Cryobacterium flavum* strain Hh8** Accession no: SOFD01000024.1	CTGCGACTGCCTCGGCGAGATTACCTACCTGAGCCCGGTCATCACCGACG
***Cryobacterium sp.* MLB32 c47** Accession no: JPRS01000047.1	CTGCGACTGCCTCGGCGAGATTACCTACCTGAGCCCGGTCATCACCGACG
***Cryobacterium luteum* strain Hh15** Accession no: SOFF01000030.1	CTGCGACTGCCTCGGCGAGATTACCTACCTGAGCCCGGTCATCACCGACG
Cryobacterium roopkundense Accession no: JPXF01000060.1	CTGCGACTGCCTCGGCGAGATTACCTACCTGAGCCCGGTCATCACCGACG
Kocuria indica Accession no: CP035504.1	CTGCGACTGCCTGGGTGACATCACCTACCTGTCCCCGGTGGTTGTGACCG
Kocuria flava Accession no: CP013254.1	CTGCGACTGCCTGGGTGACATCACCTACCTGTCCCCGGTGGTTGTGACCG
Microbacterium azadirachtae Accession no: JYIX 01000018.1	CTGCGACTGCCTGGGCGTCATCCGCTACCTCGACGGCTACGTCGCCGACG
Microbacterium oxydans Accession no:CP031422.1	CTGCGACTGCCTGGGCGTCATCCGCTACCTCGACGGCTACGTCGCCGACG
Microbacterium trichothecenolyticum Accession no: JYJA01000039.1	CTGCGACTGCCTGGGCGTCATCCGCTACCTCGACGGCTACGTCGCCGACG
Microbacterium foliorum Accession no: JYIU01000019.1	CTGCGACTGCCTGGGCGTCATCCGCTACCTCGACGGCTACGTCGCCGACG
Microbacterium mangrove Accession no: JTDK01000001.1	CTGCGACTGCCTGGGCGTCATCCGCTACCTCGACGGCTACGTCGCCGACG
***Nocardiopsis sp.* NRRL B-16309** Accession no: LGEC01000081.1	CTGCGACTGCCTCGGCGACATCACCTACATGGACGCCGTCCTCGCCGACG
Paenarthrobacter aurescens Accession no: CP000474.1	CTGCGATTGCCTGGGTGACATCACGTACCTCAGCCCTGTCATCAGCGATG
PseudArthrobacter sp. Accession no: CP041198.1	CTGTGACTGCCTGGGCGAGATCACCTACCTCAGCCCGGTCATCTCCGACG
Pseudarthrobacter chlorophenolicus A6 Accession no: CP001341.1	CTGTGACTGCCTGGGCGAGATCACCTACCTCAGCCCGGTCATCTCCGACG
***Rhodococcus jostii* RHA1** Accession no: CP000431.1	CTGCGACTGCGTCGGCGACATCACCTACTTCGACGCCGTCATCGCCGACG
***Rhodococcus opacus* 1CP** Accession no: CP009111.1	CTGCGACTGCGTCGGCGACATCACCTACTTCGACGCCGTCATCGCCGACG
Rhodococcus ruber Accession no: CCSD01000085.1	CTGCGACTGCGTCGGCGACATCACCTACTTCGACGCCGTCATCGCCGACG
Rhodococcus wratislaviensis Accession no:BHYM01000005.1	CTGCGACTGCGTCGGCGACATCACCTACTTCGACGCCGTCATCGCCGACG
***Rhodococcus sp.*SC4 SC4** Accession no: LSBM01000283.1	CTGCGACTGCGTCGGCGACATCACCTACTTCGACGCCGTCATCGCCGACG
Saccharothrix espanaensis Accession no: He804045.1	CTGCGACTGCCTGGGCGTCATCCACTACTTCGACGCCGTGCTCGCCGACG
Sinomonas atrocyanea Accession no: CP014518.1	CTGCGACTGCCTGGGCGAGATCACCTACCTCAGCCCCGTGGTGGCCGACG
Streptomyces ossamyceticus Accession no: NZ_RJKY01000001.1	CTGTGACTGCCTCGGCGACATCACCTACCTGGACGCCGTCATCGCCGACG
Streptomyces fulvoviolaceus Accession no: NZ_JOEY01000080.1	CTGTGACTGCCTCGGCGACATCACCTACCTGGACGCCGTCATCGCCGACG
Streptomyces torulosus Accession no: NZ_LIRK01000041.1	CTGTGACTGCCTCGGCGACATCACCTACCTGGACGCCGTCATCGCCGACG
Streptomyces xylophagus Accession no: NZ_JNW001000006.1	CTGTGACTGCCTCGGCGACATCACCTACCTGGACGCCGTCATCGCCGACG
	** ** *** * ** * ** ** * *

***Amycolatopsis thermoflava* N1165** Accessionn no: NZ_KI421511.1	AGTTCGGCCACCCCCGCGTGCTGCCCAACGCGATCTGCCTGCACGAGGAG
Amycolatopsis japonica strain MG417-CF17 Accession no: CP008953.1	AGTTCGGCCACCCCCGCGTGCTGCCCAACGCGATCTGCCTGCACGAGGAG
***Arthrobacter alpinus* ERGS4** Accession no: CP013200.1	CCTTCGGCAACCCGCGCGAAATTCGCAACGGCATCTGCATGCACGAGGAG
Arthrobacter sp. 31Y K253DRAFT Accession no: JAFW01000002.1	CCTTCGGCAACCCGCGCGAAATTCGCAACGGCATCTGCATGCACGAGGAG
Arthrobacter sp. M2012083 Accession no: NZ_AKKK01000062.1	CCTTCGGCAACCCGCGCGAAATTCGCAACGGCATCTGCATGCACGAGGAG
***Arthrobacter sp.* EpRS71** Accession no: LNUV01000006.1	CCTTCGGCAACCCGCGCGAAATTCGCAACGGCATCTGCATGCACGAGGAG
Arthrobacter crystallopoietes Accession no: AB240436.1	CCTTCGGCAACCCGCGCGAAATTCGCAACGGCATCTGCATGCACGAGGAG
Brevibacterium linens Accession no: CP014869.1	ACCTCGGCGAACCTCGGCAGATCAAGAACGGCATCTGCATGCACGAGGAG
***Brevibacterium sp.* YB235** Accession no: CP050153.1	ACCTCGGCGAACCTCGGCAGATCAAGAACGGCATCTGCATGCACGAGGAG
***Cryobacterium flavum* strain Hh8** Accession no: SOFD01000024.1	GCTTCGGCAACCCGCGCGAGATTCGCAACGGTATCTGCATGCACGAAGAG
***Cryobacterium sp.* MLB32 c47** Accession no: JPRS01000047.1	GCTTCGGCAACCCGCGCGAGATTCGCAACGGTATCTGCATGCACGAAGAG
***Cryobacterium luteum* strain Hh15** Accession no: SOFF01000030.1	GCTTCGGCAACCCGCGCGAGATTCGCAACGGTATCTGCATGCACGAAGAG
Cryobacterium roopkundense Accession no: JPXF01000060.1	GCTTCGGCAACCCGCGCGAGATTCGCAACGGTATCTGCATGCACGAAGAG
Kocuria indica Accession no: CP035504.1	GGCGCGGCGAACCGCGGGAGATCACCAACGGCATCTGCATGCACGAGGAG
Kocuria flava Accession no: CP013254.1	GGCGCGGCGAACCGCGGGAGATCACCAACGGCATCTGCATGCACGAGGAG
Microbacterium azadirachtae Accession no: JYIX 01000018.1	ACCAGGGCCATCCGGTGCGGATCCCGAACGTGATCTGCATGCACGAGGAG
Microbacterium oxydans Accession no:CP031422.1	ACCAGGGCCATCCGGTGCGGATCCCGAACGTGATCTGCATGCACGAGGAG
Microbacterium trichothecenolyticum Accession no: JYJA01000039.1	ACCAGGGCCATCCGGTGCGGATCCCGAACGTGATCTGCATGCACGAGGAG
Microbacterium foliorum Accession no: JYIU01000019.1	ACCAGGGCCATCCGGTGCGGATCCCGAACGTGATCTGCATGCACGAGGAG
Microbacterium mangrove Accession no: JTDK01000001.1	ACCAGGGCCATCCGGTGCGGATCCCGAACGTGATCTGCATGCACGAGGAG
***Nocardiopsis sp.* NRRL B-16309** Accession no: LGEC01000081.1	AGCGCGGAGAGCCACAGACCCTCCCCAACGCCGTGTGCCTGCACGAGGAG
Paenarthrobacter aurescens Accession no: CP000474.1	CTTTCGGCAACCCACGCGAGATCCGCAACGGCATCTGCATGCACGAGGAA
PseudArthrobacter sp. Accession no: CP041198.1	CGTTCGGAAACCCCCGCGAGATCCGCAACGGCATCTGCATGCACGAGGAG
Pseudarthrobacter chlorophenolicus A6 Accession no: CP001341.1	CGTTCGGAAACCCCCGCGAGATCCGCAACGGCATCTGCATGCACGAGGAG
***Rhodococcus jostii* RHA1** Accession no: CP000431.1	AGTTCGGCACCCCCAAGACGCTGAAGAACGCCGTGTGCATGCACGAGGAG
***Rhodococcus opacus* 1CP** Accession no: CP009111.1	AGTTCGGCACCCCCAAGACGCTGAAGAACGCCGTGTGCATGCACGAGGAG
Rhodococcus ruber Accession no: CCSD01000085.1	AGTTCGGCACCCCCAAGACGCTGAAGAACGCCGTGTGCATGCACGAGGAG
Rhodococcus wratislaviensis Accession no:BHYM01000005.1	AGTTCGGCACCCCCAAGACGCTGAAGAACGCCGTGTGCATGCACGAGGAG
***Rhodococcus sp.SC4* SC4** Accession no: LSBM01000283.1	AGTTCGGCACCCCCAAGACGCTGAAGAACGCCGTGTGCATGCACGAGGAG
Saccharothrix espanaensis Accession no: He804045.1	ACCACGGCCACCCGGTGGCCATCCCCCAGGCGATCTGCATGCACGAGGAG
Sinomonas atrocyanea Accession no: CP014518.1	GGTTCGGCACCCCGCGCACCATCGCGAACGGCATCTGCATGCACGAGGAG
Streptomyces ossamyceticus Accession no: NZ_RJKY01000001.1	ACACGGGTGCCCCGCGCACCCTGCGCAACGCGATCTGTCTGCACGAGGAG
Streptomyces fulvoviolaceus Accession no: NZ_JOEY01000080.1	ACACGGGTGCCCCGCGCACCCTGCGCAACGCGATCTGTCTGCACGAGGAG
Streptomyces torulosus Accession no: NZ_LIRK01000041.1	ACACGGGTGCCCCGCGCACCCTGCGCAACGCGATCTGTCTGCACGAGGAG
Streptomyces xylophagus Accession no: NZ_JNW001000006.1	ACACGGGTGCCCCGCGCACCCTGCGCAACGCGATCTGTCTGCACGAGGAG
	** ** * * * * ** ******* **
***Amycolatopsis thermoflava* N1165** Accessionn no: NZ_KI421511.1	GACTACGGGATCCTCTGGAAGCACACCGACCTGTGGGCGGGCTCGGCGGA
Amycolatopsis japonica strain MG417-CF17 Accession no: CP008953.1	GACTACGGGATCCTCTGGAAGCACACCGACCTGTGGGCGGGCTCGGCGGA
***Arthrobacter alpinus* ERGS4** Accession no: CP013200.1	GACGCCTCGATCCTGGCCAAGCACTCGGATCTGTGGACCGGTATCAACTA
Arthrobacter sp. 31Y K253DRAFT Accession no: JAFW01000002.1	GACGCCTCGATCCTGGCCAAGCACTCGGATCTGTGGACCGGTATCAACTA
Arthrobacter sp. M2012083 Accession no: NZ_AKKK01000062.1	GACGCCTCGATCCTGGCCAAGCACTCGGATCTGTGGACCGGTATCAACTA
***Arthrobacter sp.* EpRS71** Accession no: LNUV01000006.1	GACGCCTCGATCCTGGCCAAGCACTCGGATCTGTGGACCGGTATCAACTA
Arthrobacter crystallopoietes Accession no: AB240436.1	GACGCCTCGATCCTGGCCAAGCACTCGGATCTGTGGACCGGTATCAACTA
Brevibacterium linens Accession no: CP014869.1	GACGCGTCGATCCTGGCCAAACATTCGGACCTGTGGTCGGGCGTCGCCTA
***Brevibacterium sp.* YB235** Accession no: CP050153.1	GACGCGTCGATCCTGGCCAAACATTCGGACCTGTGGTCGGGCGTCGCCTA
***Cryobacterium flavum* strain Hh8** Accession no: SOFD01000024.1	GATTGGAGCATCCTCGCCAAGCACAGCGACCTGTGGAGCGGCATCGAATA
***Cryobacterium sp.* MLB32 c47** Accession no: JPRS01000047.1	GATTGGAGCATCCTCGCCAAGCACAGCGACCTGTGGAGCGGCATCGAATA
***Cryobacterium luteum* strain Hh15** Accession no: SOFF01000030.1	GATTGGAGCATCCTCGCCAAGCACAGCGACCTGTGGAGCGGCATCGAATA
Cryobacterium roopkundense Accession no: JPXF01000060.1	GATTGGAGCATCCTCGCCAAGCACAGCGACCTGTGGAGCGGCATCGAATA
Kocuria indica Accession no: CP035504.1	GACTGGTCCATCCTCTCCAAGCACACGGACCTGTGGAGCGGGGTGGCGTA
Kocuria flava Accession no: CP013254.1	GACTGGTCCATCCTCTCCAAGCACACGGACCTGTGGAGCGGGGTGGCGTA
Microbacterium azadirachtae Accession no: JYIX 01000018.1	GACACCGGGATCCTCTGGAAGCACACGAACCTCG---AGGGCCGCTCCGA
Microbacterium oxydans Accession no:CP031422.1	GACACCGGGATCCTCTGGAAGCACACGAACCTCG---AGGGCCGCTCCGA
Microbacterium trichothecenolyticum Accession no: JYJA01000039.1	GACACCGGGATCCTCTGGAAGCACACGAACCTCG---AGGGCCGCTCCGA
Microbacterium foliorum Accession no: JYIU01000019.1	GACACCGGGATCCTCTGGAAGCACACGAACCTCG---AGGGCCGCTCCGA

Microbacterium mangrove Accession no: JTDK01000001.1	GACACCGGGATCCTCTGGAAGCACACGAACCTCG---AGGGCCGCTCCGA
***Nocardiopsis sp.* NRRL B-16309** Accession no: LGEC01000081.1	GACGTCGGGGTGCTGTGGAAGCACAGCGACCTGTGGGCGGGATCGTCCGA
Paenarthrobacter aurescens Accession no: CP000474.1	GACTGGGGCATCCTCTCCAAGCACTCTGACCTGTGGACCGGCATCAATTA
PseudArthrobacter sp. Accession no: CP041198.1	GACTGGAGCATCCTCTCCAAGCACTCCGACCTCTGGAGCGGAATCACCTA
Pseudarthrobacter chlorophenolicus A6 Accession no: CP001341.1	GACTGGAGCATCCTCTCCAAGCACTCCGACCTCTGGAGCGGAATCACCTA
***Rhodococcus jostii* RHA1** Accession no: CP000431.1	GACTTCGGCGTGCTGTGGAAGCACACCGACCTGTGGGCCGGCTCCGCCGA
***Rhodococcus opacus* 1CP** Accession no: CP009111.1	GACTTCGGCGTGCTGTGGAAGCACACCGACCTGTGGGCCGGCTCCGCCGA
Rhodococcus ruber Accession no: CCSD01000085.1	GACTTCGGCGTGCTGTGGAAGCACACCGACCTGTGGGCCGGCTCCGCCGA
Rhodococcus wratislaviensis Accession no:BHYM01000005.1	GACTTCGGCGTGCTGTGGAAGCACACCGACCTGTGGGCCGGCTCCGCCGA
***Rhodococcus sp.*SC4 SC4** Accession no: LSBM01000283.1	GACTTCGGCGTGCTGTGGAAGCACACCGACCTGTGGGCCGGCTCCGCCGA
Saccharothrix espanaensis Accession no: He804045.1	GACTACGGCGTGCAGTGGAAGCACACCAACATCCTCACCGGGGCGTCGGA
Sinomonas atrocyanea Accession no: CP014518.1	GACGCCTCGATCCTCTCCAAGCACTCCGACCTGTGGAGCGGGGTCAGCTA
Streptomyces ossamyceticus Accession no: NZ_RJKY01000001.1	GACCACGGCATCCTCTGGAAGCATACCGACCTGTGGACCGGCCGCGCCGA
Streptomyces fulvoviolaceus Accession no: NZ_JOEY01000080.1	GACCACGGCATCCTCTGGAAGCATACCGACCTGTGGACCGGCCGCGCCGA
Streptomyces torulosus Accession no: NZ_LIRK01000041.1	GACCACGGCATCCTCTGGAAGCATACCGACCTGTGGACCGGCCGCGCCGA
Streptomyces xylophagus Accession no: NZ_JNW001000006.1	GACCACGGCATCCTCTGGAAGCATACCGACCTGTGGACCGGCCGCGCCGA
	** * * ** ** * * ** *
***Amycolatopsis thermoflava* N1165** Accessionn no: NZ_KI421511.1	GACCCGCCGCCAGCGCCGCATGGTGATCTCGTTCTTCACCACGATCGGCA
***Amycolatopsis japonica* strain MG417-CF17** Accession no: CP008953.1	GACCCGCCGCCAGCGCCGCATGGTGATCTCGTTCTTCACCACGATCGGCA
***Arthrobacter alpinus* ERGS4** Accession no: CP013200.1	CACCCGCCGCAACCGCCGCCTGGTGGTCTCCTTCTTTACCACCATTGGCA
Arthrobacter sp. 31Y K253DRAFT Accession no: JAFW01000002.1	CACCCGCCGCAACCGCCGCCTGGTGGTCTCCTTCTTTACCACCATTGGCA
Arthrobacter sp. M2012083 Accession no: NZ_AKKK01000062.1	CACCCGCCGCAACCGCCGCCTGGTGGTCTCCTTCTTTACCACCATTGGCA
***Arthrobacter sp.* EpRS71** Accession no: LNUV01000006.1	CACCCGCCGCAACCGCCGCCTGGTGGTCTCCTTCTTTACCACCATTGGCA
Arthrobacter crystallopoietes Accession no: AB240436.1	CACCCGCCGCAACCGCCGCCTGGTGGTCTCCTTCTTTACCACCATTGGCA
Brevibacterium linens Accession no: CP014869.1	CACCCGGCGCAACCGCCGCTTCGTCGTCTCGTTCTTCACCACGGTTGGCA
***Brevibacterium sp.* YB235** Accession no: CP050153.1	CACCCGGCGCAACCGCCGCTTCGTCGTCTCGTTCTTCACCACGGTTGGCA
***Cryobacterium flavum* strain Hh8** Accession no: SOFD01000024.1	CACCCGCCGCAACCGCCGCTTCGTCATCAGCTTCTTCACCACCGTCGGCA
***Cryobacterium sp.* MLB32 c47** Accession no: JPRS01000047.1	CACCCGCCGCAACCGCCGCTTCGTCATCAGCTTCTTCACCACCGTCGGCA
***Cryobacterium luteum* strain Hh15** Accession no: SOFF01000030.1	CACCCGCCGCAACCGCCGCTTCGTCATCAGCTTCTTCACCACCGTCGGCA
Cryobacterium roopkundense Accession no: JPXF01000060.1	CACCCGCCGCAACCGCCGCTTCGTCATCAGCTTCTTCACCACCGTCGGCA
Kocuria indica Accession no: CP035504.1	CACCCGCCGCAACCGCCGCCTGGTGATCTCCTTCTTCACCACCGTGGGCA
Kocuria flava Accession no: CP013254.1	CACCCGCCGCAACCGCCGCCTGGTGATCTCCTTCTTCACCACCGTGGGCA
Microbacterium azadirachtae Accession no: JYIX 01000018.1	CGTGCGCCGCGCCCGCCGCTTCGTGGTGTCGTATTTCTCCACGATCGGCA
Microbacterium oxydans Accession no:CP031422.1	CGTGCGCCGCGCCCGCCGCTTCGTGGTGTCGTATTTCTCCACGATCGGCA
Microbacterium trichothecenolyticum Accession no: JYJA01000039.1	CGTGCGCCGCGCCCGCCGCTTCGTGGTGTCGTATTTCTCCACGATCGGCA
Microbacterium foliorum Accession no: JYIU01000019.1	CGTGCGCCGCGCCCGCCGCTTCGTGGTGTCGTATTTCTCCACGATCGGCA
Microbacterium mangrove Accession no: JTDK01000001.1	CGTGCGCCGCGCCCGCCGCTTCGTGGTGTCGTATTTCTCCACGATCGGCA
***Nocardiopsis sp.* NRRL B-16309** Accession no: LGEC01000081.1	GACCCGGCGCCGGCGGCGGATGGTGATCTCCTTCTTCACCACCGTCGGCA
Paenarthrobacter aurescens Accession no: CP000474.1	CACCCGCCGCAACCGCCGGCTGGTGATCTCCTTCTTCACTACCATCGGCA
PseudArthrobacter sp. Accession no: CP041198.1	CACCCGCCGCAACCGCCGCCTGGTGATCTCCTTCTTCACCACCATCGGTA
Pseudarthrobacter chlorophenolicus A6 Accession no: CP001341.1	CACCCGCCGCAACCGCCGCCTGGTGATCTCCTTCTTCACCACCATCGGTA
***Rhodococcus jostii* RHA1** Accession no: CP000431.1	GACTCGCCGCCAGCGCCGTCTCGTCATCTCCTTCTTCACCACCATCGGCA
***Rhodococcus opacus* 1CP** Accession no: CP009111.1	GACTCGCCGCCAGCGCCGTCTCGTCATCTCCTTCTTCACCACCATCGGCA
Rhodococcus ruber Accession no: CCSD01000085.1	GACTCGCCGCCAGCGCCGTCTCGTCATCTCCTTCTTCACCACCATCGGCA
Rhodococcus wratislaviensis Accession no:BHYM01000005.1	GACTCGCCGCCAGCGCCGTCTCGTCATCTCCTTCTTCACCACCATCGGCA
***Rhodococcus sp.*SC4 SC4** Accession no: LSBM01000283.1	GACTCGCCGCCAGCGCCGTCTCGTCATCTCCTTCTTCACCACCATCGGCA
Saccharothrix espanaensis Accession no: He804045.1	CGTGCGGCGCTCGCGCCGGCTGGTGGTGTCCTCGTTCAGCACCATCGGCA
Sinomonas atrocyanea Accession no: CP014518.1	CGTGCGCCGCAACCGCCGCCTCGTCATCAGTTTCTTCACCACGATCGGCA
Streptomyces ossamyceticus Accession no: NZ_RJKY01000001.1	GACCCGCCGCCAGCGCCGCATGGTGCTCTCCTTCTTCACCACCATCGGCA
Streptomyces fulvoviolaceus Accession no: NZ_JOEY01000080.1	GACCCGCCGCCAGCGCCGCATGGTGCTCTCCTTCTTCACCACCATCGGCA
Streptomyces torulosus Accession no: NZ_LIRK01000041.1	GACCCGCCGCCAGCGCCGCATGGTGCTCTCCTTCTTCACCACCATCGGCA
Streptomyces xylophagus Accession no: NZ_JNW001000006.1	GACCCGCCGCCAGCGCCGCATGGTGCTCTCCTTCTTCACCACCATCGGCA
	** *** ** ** * ** * * ** ** * ** *
***Amycolatopsis thermoflava* N1165** Accessionn no: NZ_KI421511.1	ACTACGACTACGGCTTCTACTGGTACCTCTACCTGGACGGCACGATCGAG

***Amycolatopsis japonica* strain MG417-CF17** Accession no: CP008953.1	ACTACGACTACGGCTTCTACTGGTACCTCTACCTGGACGGCACGATCGAG
***Arthrobacter alpinus* ERGS4** Accession no: CP013200.1	ACTACGACTACGGCTTTTACTGGTACCTCTACCTGGACGGCACCATCGAA
Arthrobacter sp. 31Y K253DRAFT Accession no: JAFW01000002.1	ACTACGACTACGGCTTTTACTGGTACCTCTACCTGGACGGCACCATCGAA
Arthrobacter sp. M2012083 Accession no: NZ_AKKK01000062.1	ACTACGACTACGGCTTTTACTGGTACCTCTACCTGGACGGCACCATCGAA
***Arthrobacter sp.* EpRS71** Accession no: LNUV01000006.1	ACTACGACTACGGCTTTTACTGGTACCTCTACCTGGACGGCACCATCGAA
Arthrobacter crystallopoietes Accession no: AB240436.1	ACTACGACTACGGCTTTTACTGGTACCTCTACCTGGACGGCACCATCGAA
Brevibacterium linens Accession no: CP014869.1	ACTACGACTACGGCTTCTACTGGTACCTCTACCTCGACGGCACTATCGAA
***Brevibacterium sp.* YB235** Accession no: CP050153.1	ACTACGACTACGGCTTCTACTGGTACCTCTACCTCGACGGCACTATCGAA
***Cryobacterium flavum* strain Hh8** Accession no: SOFD01000024.1	ACTACGACTACGGCTTCTACTGGTACCTCTACCTCGACGGCACCATCGAA
***Cryobacterium sp.* MLB32 c47** Accession no: JPRS01000047.1	ACTACGACTACGGCTTCTACTGGTACCTCTACCTCGACGGCACCATCGAA
***Cryobacterium luteum* strain Hh15** Accession no: SOFF01000030.1	ACTACGACTACGGCTTCTACTGGTACCTCTACCTCGACGGCACCATCGAA
Cryobacterium roopkundense Accession no: JPXF01000060.1	ACTACGACTACGGCTTCTACTGGTACCTCTACCTCGACGGCACCATCGAA
Kocuria indica Accession no: CP035504.1	ACTACGACTACGGCTTCTACTGGTACCTCTACCTGGACGGCACCATCGAG
Kocuria flava Accession no: CP013254.1	ACTACGACTACGGCTTCTACTGGTACCTCTACCTGGACGGCACCATCGAG
Microbacterium azadirachtae Accession no: JYIX 01000018.1	ACTACGACTACGGCTTCTACTGGAACTTCGGCCTGGACGGCACGATCGAG
Microbacterium oxydans Accession no:CP031422.1	ACTACGACTACGGCTTCTACTGGAACTTCGGCCTGGACGGCACGATCGAG
Microbacterium trichothecenolyticum Accession no: JYJA01000039.1	ACTACGACTACGGCTTCTACTGGAACTTCGGCCTGGACGGCACGATCGAG
Microbacterium foliorum Accession no: JYIU01000019.1	ACTACGACTACGGCTTCTACTGGAACTTCGGCCTGGACGGCACGATCGAG
Microbacterium mangrove Accession no: JTDK01000001.1	ACTACGACTACGGCTTCTACTGGAACTTCGGCCTGGACGGCACGATCGAG
***Nocardiopsis sp.* NRRL B-16309** Accession no: LGEC01000081.1	ACTACGACTACGGGTTCTACTGGTACCTCTACCTCGACGGCACGATCGAG
Paenarthrobacter aurescens Accession no: CP000474.1	ACTACGACTACGGCTTTTACTGGTACCTCTACTTGGATGGCACCATCGAA
PseudArthrobacter sp. Accession no: CP041198.1	ACTACGATTACGGCTTCTACTGGTACCTCTACCTGGACGGCACCATCGAG
Pseudarthrobacter chlorophenolicus A6 Accession no: CP001341.1	ACTACGATTACGGCTTCTACTGGTACCTCTACCTGGACGGCACCATCGAG
***Rhodococcus jostii* RHA1** Accession no: CP000431.1	ACTACGACTACGGCTTCTTCTGGTACCTGTACCTCGACGGCACCATCGAG
***Rhodococcus opacus* 1CP** Accession no: CP009111.1	ACTACGACTACGGCTTCTTCTGGTACCTGTACCTCGACGGCACCATCGAG
Rhodococcus ruber Accession no: CCSD01000085.1	ACTACGACTACGGCTTCTTCTGGTACCTGTACCTCGACGGCACCATCGAG
Rhodococcus wratislaviensis Accession no:BHYM01000005.1	ACTACGACTACGGCTTCTTCTGGTACCTGTACCTCGACGGCACCATCGAG
***Rhodococcus sp.*SC4 SC4** Accession no: LSBM01000283.1	ACTACGACTACGGCTTCTTCTGGTACCTGTACCTCGACGGCACCATCGAG
Saccharothrix espanaensis Accession no: He804045.1	ACTACGACTACGGCTTCTTCTGGTACTTCTACCTCGACGGCACGATCGAG
Sinomonas atrocyanea Accession no: CP014518.1	ACTACGACTACGGCTTCTACTGGTACCTCTACCTCGACGGCACGATCGAG
Streptomyces ossamyceticus Accession no: NZ_RJKY01000001.1	ACTACGACTACGGCTTCTACTGGTACCTCTACCTCGATGGCGCCATCGAG
Streptomyces fulvoviolaceus Accession no: NZ_JOEY01000080.1	ACTACGACTACGGCTTCTACTGGTACCTCTACCTCGATGGCGCCATCGAG
Streptomyces torulosus Accession no: NZ_LIRK01000041.1	ACTACGACTACGGCTTCTACTGGTACCTCTACCTCGATGGCGCCATCGAG
Streptomyces xylophagus Accession no: NZ_JNW001000006.1	ACTACGACTACGGCTTCTACTGGTACCTCTACCTCGATGGCGCCATCGAG
	******* ***** ** * **** ** * * * ** *** * *****
***Amycolatopsis thermoflava* N1165** Accessionn no: NZ_KI421511.1	TGCGAGGCCAAGGCCACCGGCGTCGTGTTCACCTCCGCC---TACCCCGG
***Amycolatopsis japonica* strain MG417-CF17** Accession no: CP008953.1	TGCGAGGCCAAGGCCACCGGCGTCGTGTTCACCTCCGCC---TACCCCGG
***Arthrobacter alpinus* ERGS4** Accession no: CP013200.1	TTCGAGGCCAAGGCCACTGGCATCGTCTTCACCAGCGCG---CATCCGGG
Arthrobacter sp. 31Y K253DRAFT Accession no: JAFW01000002.1	TTCGAGGCCAAGGCCACTGGCATCGTCTTCACCAGCGCG---CATCCGGG
Arthrobacter sp. M2012083 Accession no: NZ_AKKK01000062.1	TTCGAGGCCAAGGCCACTGGCATCGTCTTCACCAGCGCG---CATCCGGG
***Arthrobacter sp.* EpRS71** Accession no: LNUV01000006.1	TTCGAGGCCAAGGCCACTGGCATCGTCTTCACCAGCGCG---CATCCGGG
Arthrobacter crystallopoietes Accession no: AB240436.1	TTCGAGGCCAAGGCCACTGGCATCGTCTTCACCAGCGCG---CATCCGGG
Brevibacterium linens Accession no: CP014869.1	TTCGAGGCCAAGGCCACCGGCGTCGTCTTCACCTCGGCG---CTGCCGAA
***Brevibacterium sp.* YB235** Accession no: CP050153.1	TTCGAGGCCAAGGCCACCGGCGTCGTCTTCACCTCGGCG---CTGCCGAA
***Cryobacterium flavum* strain Hh8** Accession no: SOFD01000024.1	TTCGAGGCGAAGGCCACCGGCGTCGTCTTCACGAGCGCC---TACGCCGG
***Cryobacterium sp.* MLB32 c47** Accession no: JPRS01000047.1	TTCGAGGCGAAGGCCACCGGCGTCGTCTTCACGAGCGCC---TACGCCGG
***Cryobacterium luteum* strain Hh15** Accession no: SOFF01000030.1	TTCGAGGCGAAGGCCACCGGCGTCGTCTTCACGAGCGCC---TACGCCGG
Cryobacterium roopkundense Accession no: JPXF01000060.1	TTCGAGGCGAAGGCCACCGGCGTCGTCTTCACGAGCGCC---TACGCCGG
Kocuria indica Accession no: CP035504.1	TTCGAGGCCAAGGCCACCGGCGTGGTCTTCACCTCCGCC---CTGCCCAA
Kocuria flava Accession no: CP013254.1	TTCGAGGCCAAGGCCACCGGCGTGGTCTTCACCTCCGCC---CTGCCCAA
Microbacterium azadirachtae Accession no: JYIX 01000018.1	GTGATCGCGAAGGCCACGGGCATCGTTTTCGTGGGCGCCGGAGAGCCCGG
Microbacterium oxydans Accession no:CP031422.1	GTGATCGCGAAGGCCACGGGCATCGTTTTCGTGGGCGCCGGAGAGCCCGG
Microbacterium trichothecenolyticum Accession no: JYJA01000039.1	GTGATCGCGAAGGCCACGGGCATCGTTTTCGTGGGCGCCGGAGAGCCCGG
Microbacterium foliorum Accession no: JYIU01000019.1	GTGATCGCGAAGGCCACGGGCATCGTTTTCGTGGGCGCCGGAGAGCCCGG
Microbacterium mangrove Accession no: JTDK01000001.1	GTGATCGCGAAGGCCACGGGCATCGTTTTCGTGGGCGCCGGAGAGCCCGG

Nocardiopsis sp. **NRRL B-16309** Accession no: LGEC01000081.1	TTCGAGGCCAAGGCCACCGGCATCGTGTTCACCTCCGCC---CACCCCGG
Paenarthrobacter aurescens Accession no: CP000474.1	TTCGAAGCAAAGGCCACCGGCGTCGTATTCACCAGTGCA---TTCCCGGA
PseudArthrobacter sp. Accession no: CP041198.1	TTCGAGGCCAAGGCCACCGGCGTTGTGTTCACCTCGGCG---TTCCCCGA
Pseudarthrobacter chlorophenolicus A6 Accession no: CP001341.1	TTCGAGGCCAAGGCCACCGGCGTTGTGTTCACCTCGGCG---TTCCCCGA
Rhodococcus jostii **RHA1** Accession no: CP000431.1	TTCGAGGTCAAGGCCACCGGCATCGTCTTCACGTCGGGT---CACCCGGG
Rhodococcus opacus **1CP** Accession no: CP009111.1	TTCGAGGTCAAGGCCACCGGCATCGTCTTCACGTCGGGT---CACCCGGG
Rhodococcus ruber Accession no: CCSD01000085.1	TTCGAGGTCAAGGCCACCGGCATCGTCTTCACGTCGGGT---CACCCGGG
Rhodococcus wratislaviensis Accession no:BHYM01000005.1	TTCGAGGTCAAGGCCACCGGCATCGTCTTCACGTCGGGT---CACCCGGG
Rhodococcus sp.**SC4 SC4** Accession no: LSBM01000283.1	TTCGAGGTCAAGGCCACCGGCATCGTCTTCACGTCGGGT---CACCCGGG
Saccharothrix espanaensis Accession no: He804045.1	CTGGAGGCCAAGGCGACCGGCGTGGTGTTCTGCGGCG---------CGGG
Sinomonas atrocyanea Accession no: CP014518.1	TTCGAGGCCAAGGCCACCGGCGTCGTGTTCACCTCCGCC---CAGCCCTC
Streptomyces ossamyceticus Accession no: NZ_RJKY01000001.1	TTCGAGGCGAAGGCCACCGGCATCGTCTTCCCCTCCGCC---TACCCGGG
Streptomyces fulvoviolaceus Accession no: NZ_JOEY01000080.1	TTCGAGGCGAAGGCCACCGGCATCGTCTTCCCCTCCGCC---TACCCGGG
Streptomyces torulosus Accession no: NZ_LIRK01000041.1	TTCGAGGCGAAGGCCACCGGCATCGTCTTCCCCTCCGCC---TACCCGGG
Streptomyces xylophagus Accession no: NZ_JNW001000006.1	TTCGAGGCGAAGGCCACCGGCATCGTCTTCCCCTCCGCC---TACCCGGG
	* ***** ** *** * ** *** * *
Amycolatopsis thermoflava **N1165** Accessionn no: NZ_KI421511.1	CAAGGGCTACCCCTACGCCTCCGAACTCGCGCCCGGCCTCGGCGCCCCGT
Amycolatopsis japonica **strain MG417-CF17** Accession no: CP008953.1	CAAGGGCTACCCCTACGCCTCCGAACTCGCGCCCGGCCTCGGCGCCCCGT
Arthrobacter alpinus **ERGS4** Accession no: CP013200.1	CAAGGGCTACCCTTACGCATCCGAACTGGCGCCGGGACTTGGTGCCCCGT
Arthrobacter sp. 31Y K253DRAFT Accession no: JAFW01000002.1	CAAGGGCTACCCTTACGCATCCGAACTGGCGCCGGGACTTGGTGCCCCGT
Arthrobacter sp. M2012083 Accession no: NZ_AKKK01000062.1	CAAGGGCTACCCTTACGCATCCGAACTGGCGCCGGGACTTGGTGCCCCGT
Arthrobacter sp. **EpRS71** Accession no: LNUV01000006.1	CAAGGGCTACCCTTACGCATCCGAACTGGCGCCGGGACTTGGTGCCCCGT
Arthrobacter crystallopoietes Accession no: AB240436.1	CAAGGGCTACCCTTACGCATCCGAACTGGCGCCGGGACTTGGTGCCCCGT
Brevibacterium linens Accession no: CP014869.1	CGGGTCGACGGATTTCGCCTCGGAGATCGCGCCCGGTCTCGGTGCGCCGT
Brevibacterium sp. **YB235** Accession no: CP050153.1	CGGGTCGACGGATTTCGCCTCGGAGATCGCGCCCGGTCTCGGTGCGCCGT
Cryobacterium flavum **strain Hh8** Accession no: SOFD01000024.1	CAAGGGCTATCCCTACGCCTCCGAAATCGCTCCCGGTCTCGGCGCCCCCT
Cryobacterium sp. **MLB32 c47** Accession no: JPRS01000047.1	CAAGGGCTATCCCTACGCCTCCGAAATCGCTCCCGGTCTCGGCGCCCCCT
Cryobacterium luteum **strain Hh15** Accession no: SOFF01000030.1	CAAGGGCTATCCCTACGCCTCCGAAATCGCTCCCGGTCTCGGCGCCCCCT
Cryobacterium roopkundense Accession no: JPXF01000060.1	CAAGGGCTATCCCTACGCCTCCGAAATCGCTCCCGGTCTCGGCGCCCCCT
Kocuria indica Accession no: CP035504.1	CGGCTCCTCGGACTTCGCCTCGGAAATTGCCCCGGGGCTGGGTGCGCCGT
Kocuria flava Accession no: CP013254.1	CGGCTCCTCGGACTTCGCCTCGGAAATTGCCCCGGGGCTGGGTGCGCCGT
Microbacterium azadirachtae Accession no: JYIX 01000018.1	CGTCCCGCAGCGCCACGCCACCGAGCTCGCCCCCGGCGTGTTCGCACCGG
Microbacterium oxydans Accession no:CP031422.1	CGTCCCGCAGCGCCACGCCACCGAGCTCGCCCCCGGCGTGTTCGCACCGG
Microbacterium trichothecenolyticum Accession no: JYJA01000039.1	CGTCCCGCAGCGCCACGCCACCGAGCTCGCCCCCGGCGTGTTCGCACCGG
Microbacterium foliorum Accession no: JYIU01000019.1	CGTCCCGCAGCGCCACGCCACCGAGCTCGCCCCCGGCGTGTTCGCACCGG
Microbacterium mangrove Accession no: JTDK01000001.1	CGTCCCGCAGCGCCACGCCACCGAGCTCGCCCCCGGCGTGTTCGCACCGG
Nocardiopsis sp. **NRRL B-16309** Accession no: LGEC01000081.1	CGGCGACTACCCCTACGCGACCGAGATCGCCCCCGGACTGGGCGCGCCCT
Paenarthrobacter aurescens Accession no: CP000474.1	AGGCGGCTCGGACAACATCTCCCAGCTGGCCCCCGGCCTCGGCGCCCCGT
PseudArthrobacter sp. Accession no: CP041198.1	GGGCGGCTCGGACAACATCTCCCAGTTGGCCCCCGGCCTGGGTGCCCCGT
Pseudarthrobacter chlorophenolicus A6 Accession no: CP001341.1	GGGCGGCTCGGACAACATCTCCCAGTTGGCCCCCGGCCTGGGTGCCCCGT
Rhodococcus jostii **RHA1** Accession no: CP000431.1	CGGCGACTACCCGTACGCCTCGGAGATCGCCCCCGGCCTCGGCGCCCCGT
Rhodococcus opacus **1CP** Accession no: CP009111.1	CGGCGACTACCCGTACGCCTCGGAGATCGCCCCCGGCCTCGGCGCCCCGT
Rhodococcus ruber Accession no: CCSD01000085.1	CGGCGACTACCCGTACGCCTCGGAGATCGCCCCCGGCCTCGGCGCCCCGT
Rhodococcus wratislaviensis Accession no:BHYM01000005.1	CGGCGACTACCCGTACGCCTCGGAGATCGCCCCCGGCCTCGGCGCCCCGT
Rhodococcus sp.**SC4 SC4** Accession no: LSBM01000283.1	CGGCGACTACCCGTACGCCTCGGAGATCGCCCCCGGCCTCGGCGCCCCGT
Saccharothrix espanaensis Accession no: He804045.1	CGGCGAGAACCCGTACGGCTCGGAGATCGCCCCCGGCCTGATGGCCCCGG
Sinomonas atrocyanea Accession no: CP014518.1	CGCGGACTACCCGTACTCGTCCGAGATGGCGCCGGGCCTGGGCGCCCCGT
Streptomyces ossamyceticus Accession no: NZ_RJKY01000001.1	CCCCGGCTACCCGTACGCCTCCGAGGTCGCCCCGGGGCTCGGCGCCCCGT
Streptomyces fulvoviolaceus Accession no: NZ_JOEY01000080.1	CCCCGGCTACCCGTACGCCTCCGAGGTCGCCCCGGGGCTCGGCGCCCCGT
Streptomyces torulosus Accession no: NZ_LIRK01000041.1	CCCCGGCTACCCGTACGCCTCCGAGGTCGCCCCGGGGCTCGGCGCCCCGT
Streptomyces xylophagus Accession no: NZ_JNW001000006.1	CCCCGGCTACCCGTACGCCTCCGAGGTCGCCCCGGGGCTCGGCGCCCCGT
	* * * * ** ** ** * ** **
Amycolatopsis thermoflava **N1165** Accessionn no: NZ_KI421511.1	ACCACCAGCACCTGTTCAGCGCGCGGCTGGACATGGCCGTGGACGGGCCG
Amycolatopsis japonica **strain MG417-CF17** Accession no: CP008953.1	ACCACCAGCACCTGTTCAGCGCGCGGCTGGACATGGCCGTGGACGGGCCG

***Arthrobacter alpinus* ERGS4** Accession no: CP013200.1	ACCACCAGCACCTCTTTGGGGCGCGCCTGGATATGGCCATCGACGGCTTT
Arthrobacter sp. 31Y K253DRAFT Accession no: JAFW01000002.1	ACCACCAGCACCTCTTTGGGGCGCGCCTGGATATGGCCATCGACGGCTTT
Arthrobacter sp. M2012083 Accession no: NZ_AKKK01000062.1	ACCACCAGCACCTCTTTGGGGCGCGCCTGGATATGGCCATCGACGGCTTT
***Arthrobacter sp.* EpRS71** Accession no: LNUV01000006.1	ACCACCAGCACCTCTTTGGGGCGCGCCTGGATATGGCCATCGACGGCTTT
Arthrobacter crystallopoietes Accession no: AB240436.1	ACCACCAGCACCTCTTTGGGGCGCGCCTGGATATGGCCATCGACGGCTTT
Brevibacterium linens Accession no: CP014869.1	TCCACCAGCATCTCTTCGGTGCTCGACTCGACTTCGCCCTCGACGACGGC
***Brevibacterium sp.* YB235** Accession no: CP050153.1	TCCACCAGCATCTCTTCGGTGCTCGACTCGACTTCGCCCTCGACGACGGC
***Cryobacterium flavum* strain Hh8** Accession no: SOFD01000024.1	TTCACCAGCACCTGTTCAGCGCCCGCCTCGACATGGCCCTCGACGGCGAC
***Cryobacterium sp.* MLB32 c47** Accession no: JPRS01000047.1	TTCACCAGCACCTGTTCAGCGCCCGCCTCGACATGGCCCTCGACGGCGAC
***Cryobacterium luteum* strain Hh15** Accession no: SOFF01000030.1	TTCACCAGCACCTGTTCAGCGCCCGCCTCGACATGGCCCTCGACGGCGAC
Cryobacterium roopkundense Accession no: JPXF01000060.1	TTCACCAGCACCTGTTCAGCGCCCGCCTCGACATGGCCCTCGACGGCGAC
Kocuria indica Accession no: CP035504.1	TCCACCAGCACCTGTTCTCCGCGCGCCTGGACTTCGCGCTCGACGGCGGG
Kocuria flava Accession no: CP013254.1	TCCACCAGCACCTGTTCTCCGCGCGCCTGGACTTCGCGCTCGACGGCGGG
Microbacterium azadirachtae Accession no: JYIX 01000018.1	TGCACCAGCACCTGTTCTGCGCGCGACTCGACGTCGCGATCGACGGATCC
Microbacterium oxydans Accession no:CP031422.1	TGCACCAGCACCTGTTCTGCGCGCGACTCGACGTCGCGATCGACGGATCC
Microbacterium trichothecenolyticum Accession no: JYJA01000039.1	TGCACCAGCACCTGTTCTGCGCGCGACTCGACGTCGCGATCGACGGATCC
Microbacterium foliorum Accession no: JYIU01000019.1	TGCACCAGCACCTGTTCTGCGCGCGACTCGACGTCGCGATCGACGGATCC
Microbacterium mangrove Accession no: JTDK01000001.1	TGCACCAGCACCTGTTCTGCGCGCGACTCGACGTCGCGATCGACGGATCC
***Nocardiopsis sp.* NRRL B-16309** Accession no: LGEC01000081.1	TCCACCAGCACCTGTTCTGCGCCCGGCTCGACATGGCCCTGGACGGCACC
Paenarthrobacter aurescens Accession no: CP000474.1	TCCATCAGCACCTCTTCAGCGCCCGGCTGGACATGGCAGTTGACGGTTTC
PseudArthrobacter sp. Accession no: CP041198.1	TCCACCAGCACCTGTTCAGCGCCCGGCTGGACATGGCGGTGGACGGCTTC
Pseudarthrobacter chlorophenolicus A6 Accession no: CP001341.1	TCCACCAGCACCTGTTCAGCGCCCGGCTGGACATGGCGGTGGACGGCTTC
***Rhodococcus jostii* RHA1** Accession no: CP000431.1	ACCACCAGCACCTGTTCAGCGCCCGCCTCGACATGATGATCGACGGCGAC
***Rhodococcus opacus* 1CP** Accession no: CP009111.1	ACCACCAGCACCTGTTCAGCGCCCGCCTCGACATGATGATCGACGGCGAC
Rhodococcus ruber Accession no: CCSD01000085.1	ACCACCAGCACCTGTTCAGCGCCCGCCTCGACATGATGATCGACGGCGAC
Rhodococcus wratislaviensis Accession no:BHYM01000005.1	ACCACCAGCACCTGTTCAGCGCCCGCCTCGACATGATGATCGACGGCGAC
***Rhodococcus sp.*SC4 SC4** Accession no: LSBM01000283.1	ACCACCAGCACCTGTTCAGCGCCCGCCTCGACATGATGATCGACGGCGAC
Saccharothrix espanaensis Accession no: He804045.1	TGCACCAGCACCTGTTCTGCGCGCGGCTGGACACCGAGATCGCGGGCCCG
Sinomonas atrocyanea Accession no: CP014518.1	TCCACCAGCACCTCTTCAGCGCGCGGCTCGACTTCGCGCTCGACAGCGGG
Streptomyces ossamyceticus Accession no: NZ_RJKY01000001.1	ACCACCAGCACCTGTTCTGCGCCCGCCTGGACATGGCCGTCGACGGCCCC
Streptomyces fulvoviolaceus Accession no: NZ_JOEY01000080.1	ACCACCAGCACCTGTTCTGCGCCCGCCTGGACATGGCCGTCGACGGCCCC
Streptomyces torulosus Accession no: NZ_LIRK01000041.1	ACCACCAGCACCTGTTCTGCGCCCGCCTGGACATGGCCGTCGACGGCCCC
Streptomyces xylophagus Accession no: NZ_JNW001000006.1	ACCACCAGCACCTGTTCTGCGCCCGCCTGGACATGGCCGTCGACGGCCCC
	** ***** ** ** ** ** ** ** * *
***Amycolatopsis thermoflava* N1165** Accessionn no: NZ_KI421511.1	TCGAACCTGGTCGAAGAGGTCGACGTGGAGCGGGTGCCGATCGGGCCGGA
***Amycolatopsis japonica* strain MG417-CF17** Accession no: CP008953.1	TCGAACCTGGTCGAAGAGGTCGACGTGGAGCGGGTGCCGATCGGGCCGGA
***Arthrobacter alpinus* ERGS4** Accession no: CP013200.1	GCCAACCGTGTCGAGGAAGAGGACGTGGTGCGGGTGCCCATGGGTGAAGG
Arthrobacter sp. 31Y K253DRAFT Accession no: JAFW01000002.1	GCCAACCGTGTCGAGGAAGAGGACGTGGTGCGGGTGCCCATGGGTGAAGG
Arthrobacter sp. M2012083 Accession no: NZ_AKKK01000062.1	GCCAACCGTGTCGAGGAAGAGGACGTGGTGCGGGTGCCCATGGGTGAAGG
***Arthrobacter sp.* EpRS71** Accession no: LNUV01000006.1	GCCAACCGTGTCGAGGAAGAGGACGTGGTGCGGGTGCCCATGGGTGAAGG
Arthrobacter crystallopoietes Accession no: AB240436.1	GCCAACCGTGTCGAGGAAGAGGACGTGGTGCGGGTGCCCATGGGTGAAGG
Brevibacterium linens Accession no: CP014869.1	AAGTGCCGGGTCGAGGAGGAGGACGTGGTGCGTCTGCCGGTCTCGCAGGA
***Brevibacterium sp.* YB235** Accession no: CP050153.1	AAGTGCCGGGTCGAGGAGGAGGACGTGGTGCGTCTGCCGGTCTCGCAGGA
***Cryobacterium flavum* strain Hh8** Accession no: SOFD01000024.1	ACCAACCGGGTCGAAGAAGAAGACGTCGTGCGCGTTCCGATAGGCCCCGG
***Cryobacterium sp.* MLB32 c47** Accession no: JPRS01000047.1	ACCAACCGGGTCGAAGAAGAAGACGTCGTGCGCGTTCCGATAGGCCCCGG
***Cryobacterium luteum* strain Hh15** Accession no: SOFF01000030.1	ACCAACCGGGTCGAAGAAGAAGACGTCGTGCGCGTTCCGATAGGCCCCGG
Cryobacterium roopkundense Accession no: JPXF01000060.1	ACCAACCGGGTCGAAGAAGAAGACGTCGTGCGCGTTCCGATAGGCCCCGG
Kocuria indica Accession no: CP035504.1	CCCTGCCGCGTGGAGGAGGAGGACGCGGTGCGCGTGCCCATCTCCGACGA
Kocuria flava Accession no: CP013254.1	CCCTGCCGCGTGGAGGAGGAGGACGCGGTGCGCGTGCCCATCTCCGACGA
Microbacterium azadirachtae Accession no: JYIX 01000018.1	GACAACCGCCTCGTCGAGGTCGACGCGGCGCGCGTCCCGATGGGCCCGGA
Microbacterium oxydans Accession no:CP031422.1	GACAACCGCCTCGTCGAGGTCGACGCGGCGCGCGTCCCGATGGGCCCGGA
Microbacterium trichothecenolyticum Accession no: JYJA01000039.1	GACAACCGCCTCGTCGAGGTCGACGCGGCGCGCGTCCCGATGGGCCCGGA
Microbacterium foliorum Accession no: JYIU01000019.1	GACAACCGCCTCGTCGAGGTCGACGCGGCGCGCGTCCCGATGGGCCCGGA
Microbacterium mangrove Accession no: JTDK01000001.1	GACAACCGCCTCGTCGAGGTCGACGCGGCGCGCGTCCCGATGGGCCCGGA
***Nocardiopsis sp.* NRRL B-16309** Accession no: LGEC01000081.1	GCCAACCGTGTGGTGGAGGTGGACGCCGCCCGCGTCCCCATCGGCCCCGA

Paenarthrobacter aurescens Accession no: CP000474.1	GCCAACCGCGTGGAGGAAGAAGACGTGGTCCGGCAGGCAATGGGCGAAGG
PseudArthrobacter sp. Accession no: CP041198.1	ACCAACCGGGTAGAGGAAGAGGACGTCATCCGGCAGGTGATGGGGCCGGG
Pseudarthrobacter chlorophenolicus A6 Accession no: CP001341.1	ACCAACCGGGTAGAGGAAGAGGACGTCATCCGGCAGGTGATGGGGCCGGG
***Rhodococcus jostii* RHA1** Accession no: CP000431.1	CACAACTCCGTCGAGGAGGTACAGACCAAGCGGGTGAAGATGGGCCCCGG
***Rhodococcus opacus* 1CP** Accession no: CP009111.1	CACAACTCCGTCGAGGAGGTACAGACCAAGCGGGTGAAGATGGGCCCCGG
Rhodococcus ruber Accession no: CCSD01000085.1	CACAACTCCGTCGAGGAGGTACAGACCAAGCGGGTGAAGATGGGCCCCGG
Rhodococcus wratislaviensis Accession no:BHYM01000005.1	CACAACTCCGTCGAGGAGGTACAGACCAAGCGGGTGAAGATGGGCCCCGG
***Rhodococcus sp.*SC4 SC4** Accession no: LSBM01000283.1	CACAACTCCGTCGAGGAGGTACAGACCAAGCGGGTGAAGATGGGCCCCGG
Saccharothrix espanaensis Accession no: He804045.1	GCCAACACGGTGGAGGAGGTCGACTTCGTCGGCGTCCCGACCGGGCCGGA
Sinomonas atrocyanea Accession no: CP014518.1	CCGAACCGCGTCGAGGAGGAGGACGCCGTCCGCGTGCCGCTGGGGCCGGG
Streptomyces ossamyceticus Accession no: NZ_RJKY01000001.1	CGGAACCGGGTGGAGGAGATCGATGCCGTACGGGTGCCGATGGGACCGGA
Streptomyces fulvoviolaceus Accession no: NZ_JOEY01000080.1	CGGAACCGGGTGGAGGAGATCGATGCCGTACGGGTGCCGATGGGACCGGA
Streptomyces torulosus Accession no: NZ_LIRK01000041.1	CGGAACCGGGTGGAGGAGATCGATGCCGTACGGGTGCCGATGGGACCGGA
Streptomyces xylophagus Accession no: NZ_JNW001000006.1	CGGAACCGGGTGGAGGAGATCGATGCCGTACGGGTGCCGATGGGACCGGA
	* * * ** * * *
***Amycolatopsis thermoflava* N1165** Accessionn no: NZ_KI421511.1	CAACCCGCGCGGCAACGCCTTCACGCTCCGCAAGACGCCGCTGCGGAAGG
***Amycolatopsis japonica* strain MG417-CF17** Accession no: CP008953.1	CAACCCGCGCGGCAACGCCTTCACGCTCCGCAAGACGCCGCTGCGGAAGG
***Arthrobacter alpinus* ERGS4** Accession no: CP013200.1	GAATGAACGCGGCAATGCCTTCACGCGCAAGCGCACCATTTTGGGTACGG
Arthrobacter sp. 31Y K253DRAFT Accession no: JAFW01000002.1	GAATGAACGCGGCAATGCCTTCACGCGCAAGCGCACCATTTTGGGTACGG
Arthrobacter sp. M2012083 Accession no: NZ_AKKK01000062.1	GAATGAACGCGGCAATGCCTTCACGCGCAAGCGCACCATTTTGGGTACGG
***Arthrobacter sp.* EpRS71** Accession no: LNUV01000006.1	GAATGAACGCGGCAATGCCTTCACGCGCAAGCGCACCATTTTGGGTACGG
Arthrobacter crystallopoietes Accession no: AB240436.1	GAATGAACGCGGCAATGCCTTCACGCGCAAGCGCACCATTTTGGGTACGG
Brevibacterium linens Accession no: CP014869.1	CAATCCGCGCGGCAACGCCTTCAGCCGGTCCCGAACCATTCTGGCAACCG
***Brevibacterium sp.* YB235** Accession no: CP050153.1	CAATCCGCGCGGCAACGCCTTCAGCCGGTCCCGAACCATTCTGGCAACCG
***Cryobacterium flavum* strain Hh8** Accession no: SOFD01000024.1	CAACGAGCGCGGCAACGCCTTCACCCGCCAGCGCACCGTGCTCGCCACCG
***Cryobacterium sp.* MLB32 c47** Accession no: JPRS01000047.1	CAACGAGCGCGGCAACGCCTTCACCCGCCAGCGCACCGTGCTCGCCACCG
***Cryobacterium luteum* strain Hh15** Accession no: SOFF01000030.1	CAACGAGCGCGGCAACGCCTTCACCCGCCAGCGCACCGTGCTCGCCACCG
Cryobacterium roopkundense Accession no: JPXF01000060.1	CAACGAGCGCGGCAACGCCTTCACCCGCCAGCGCACCGTGCTCGCCACCG
Kocuria indica Accession no: CP035504.1	CAACTCCCGCGGCAACGCGTTCACCCGCAGGCGCACCGTGCTGGAGACGG
Kocuria flava Accession no: CP013254.1	CAACTCCCGCGGCAACGCGTTCACCCGCAGGCGCACCGTGCTGGAGACGG
Microbacterium azadirachtae Accession no: JYIX 01000018.1	GAACCCGTTCGGCAACGCGTTCAGCTGGACCGAGACCGCCCTCGGATCCG
Microbacterium oxydans Accession no:CP031422.1	GAACCCGTTCGGCAACGCGTTCAGCTGGACCGAGACCGCCCTCGGATCCG
Microbacterium trichothecenolyticum Accession no: JYJA01000039.1	GAACCCGTTCGGCAACGCGTTCAGCTGGACCGAGACCGCCCTCGGATCCG
Microbacterium foliorum Accession no: JYIU01000019.1	GAACCCGTTCGGCAACGCGTTCAGCTGGACCGAGACCGCCCTCGGATCCG
Microbacterium mangrove Accession no: JTDK01000001.1	GAACCCGTTCGGCAACGCGTTCAGCTGGACCGAGACCGCCCTCGGATCCG
***Nocardiopsis sp.* NRRL B-16309** Accession no: LGEC01000081.1	CAATCCGCGCGGCAACGCCTTCACCCAGAAGCACACCGTGCTGGCCTCCG
Paenarthrobacter aurescens Accession no: CP000474.1	CAACGAGCGTGGCAATGCGTTCTCCCGCAAGCGCACTGTCCTGGCCACAG
PseudArthrobacter sp. Accession no: CP041198.1	GAACGAGCGTGGCAACGCGTTCTCCCGCCGGCGCACGCTGCTGGCCCGGG
Pseudarthrobacter chlorophenolicus A6 Accession no: CP001341.1	GAACGAGCGTGGCAACGCGTTCTCCCGCCGGCGCACGCTGCTGGCCCGGG
***Rhodococcus jostii* RHA1** Accession no: CP000431.1	GAACCTGCACGGAAACGCGTTCACCCTCGAGCGCACGCCGCTGACGAAGG
***Rhodococcus opacus* 1CP** Accession no: CP009111.1	GAACCTGCACGGAAACGCGTTCACCCTCGAGCGCACGCCGCTGACGAAGG
Rhodococcus ruber Accession no: CCSD01000085.1	GAACCTGCACGGAAACGCGTTCACCCTCGAGCGCACGCCGCTGACGAAGG
Rhodococcus wratislaviensis Accession no:BHYM01000005.1	GAACCTGCACGGAAACGCGTTCACCCTCGAGCGCACGCCGCTGACGAAGG
***Rhodococcus sp.*SC4 SC4** Accession no: LSBM01000283.1	GAACCTGCACGGAAACGCGTTCACCCTCGAGCGCACGCCGCTGACGAAGG
Saccharothrix espanaensis Accession no: He804045.1	CAACCCGCGCGGCAACGCCTTCACCACCACGACGACCGTGCTCGACCGCG
Sinomonas atrocyanea Accession no: CP014518.1	GAACGAGCGCGGCAACGCGTTCACCCGCAGGCGCACGCTCCTGGCCCGCG
Streptomyces ossamyceticus Accession no: NZ_RJKY01000001.1	CAACCCCCGCGGCAACGCGTTCACCCTCCGGCGCACCCCGCTGCGCACCG
Streptomyces fulvoviolaceus Accession no: NZ_JOEY01000080.1	CAACCCCCGCGGCAACGCGTTCACCCTCCGGCGCACCCCGCTGCGCACCG
Streptomyces torulosus Accession no: NZ_LIRK01000041.1	CAACCCCCGCGGCAACGCGTTCACCCTCCGGCGCACCCCGCTGCGCACCG
Streptomyces xylophagus Accession no: NZ_JNW001000006.1	CAACCCCCGCGGCAACGCGTTCACCCTCCGGCGCACCCCGCTGCGCACCG
	** ** ** ** *** ** * *
***Amycolatopsis thermoflava* N1165** Accessionn no: NZ_KI421511.1	AATCCGAAGCGCAGCGGCTCGCCGACAACCGGCGCGGCCGGGTCTGGCAC
***Amycolatopsis japonica* strain MG417-CF17** Accession no: CP008953.1	AATCCGAAGCGCAGCGGCTCGCCGACAACCGGCGCGGCCGGGTCTGGCAC
***Arthrobacter alpinus* ERGS4** Accession no: CP013200.1	AATCCGAGGCTGTCCGTGAGGCGGACATGCGTGCCGGGCGGACCTGGATC

Arthrobacter sp. 31Y K253DRAFT Accession no: JAFW01000002.1	AATCCGAGGCTGTCCGTGAGGCGGACATGCGTGCCGGGCGGACCTGGATC
Arthrobacter sp. M2012083 Accession no: NZ_AKKK01000062.1	AATCCGAGGCTGTCCGTGAGGCGGACATGCGTGCCGGGCGGACCTGGATC
***Arthrobacter sp.* EpRS71** Accession no: LNUV01000006.1	AATCCGAGGCTGTCCGTGAGGCGGACATGCGTGCCGGGCGGACCTGGATC
Arthrobacter crystallopoietes Accession no: AB240436.1	AATCCGAGGCTGTCCGTGAGGCGGACATGCGTGCCGGGCGGACCTGGATC
Brevibacterium linens Accession no: CP014869.1	AGTCAGCTGCACAACGAGATGCGGATCAGTCGAAGGCGCGGACCTGGGTG
***Brevibacterium sp.* YB235** Accession no: CP050153.1	AGTCAGCTGCACAACGAGATGCGGATCAGTCGAAGGCGCGGACCTGGGTG
***Cryobacterium flavum* strain Hh8** Accession no: SOFD01000024.1	AGCTCGAGGGCGTCAGACAAGCGGATGCCGCCATCGGCCGCACCTGGCAC
***Cryobacterium sp.* MLB32 c47** Accession no: JPRS01000047.1	AGCTCGAGGGCGTCAGACAAGCGGATGCCGCCATCGGCCGCACCTGGCAC
***Cryobacterium luteum* strain Hh15** Accession no: SOFF01000030.1	AGCTCGAGGGCGTCAGACAAGCGGATGCCGCCATCGGCCGCACCTGGCAC
Cryobacterium roopkundense Accession no: JPXF01000060.1	AGCTCGAGGGCGTCAGACAAGCGGATGCCGCCATCGGCCGCACCTGGCAC
Kocuria indica Accession no: CP035504.1	AGAAGGCCGCCATGCGGGACGCGGACATGTCCCGGGCGCGCACGTGGGTG
Kocuria flava Accession no: CP013254.1	AGAAGGCCGCCATGCGGGACGCGGACATGTCCCGGGCGCGCACGTGGGTG
Microbacterium azadirachtae Accession no: JYIX 01000018.1	AGTCCGCCGCGCAGCGCGACGCCGACACCTCCGTCGCCCGGGTCTGGGAG
Microbacterium oxydans Accession no:CP031422.1	AGTCCGCCGCGCAGCGCGACGCCGACACCTCCGTCGCCCGGGTCTGGGAG
Microbacterium trichothecenolyticum Accession no: JYJA01000039.1	AGTCCGCCGCGCAGCGCGACGCCGACACCTCCGTCGCCCGGGTCTGGGAG
Microbacterium foliorum Accession no: JYIU01000019.1	AGTCCGCCGCGCAGCGCGACGCCGACACCTCCGTCGCCCGGGTCTGGGAG
Microbacterium mangrove Accession no: JTDK01000001.1	AGTCCGCCGCGCAGCGCGACGCCGACACCTCCGTCGCCCGGGTCTGGGAG
***Nocardiopsis sp.* NRRL B-16309** Accession no: LGEC01000081.1	AGAACCAGGCCCAGCGCGACGCCGACGCCTCCGTCGACCGGGTCTGGCAC
Paenarthrobacter aurescens Accession no: CP000474.1	AGTCCCAGGCCGTCCGCGAAGCCGATGCCCGCGCAGGCCGCACCTGGATC
PseudArthrobacter sp. Accession no: CP041198.1	AATCCGACGGCGTCCGGGAGGCCGATGCGCGGGCCGGCCGCACTTGGATC
Pseudarthrobacter chlorophenolicus A6 Accession no: CP001341.1	AATCCGACGGCGTCCGGGAGGCCGATGCGCGGGCCGGCCGCACTTGGATC
***Rhodococcus jostii* RHA1** Accession no: CP000431.1	AATCCGAGGCGCAGCGCCTCGCGGACAACAGCGTCGGACGCGTGTGGCAC
***Rhodococcus opacus* 1CP** Accession no: CP009111.1	AATCCGAGGCGCAGCGCCTCGCGGACAACAGCGTCGGACGCGTGTGGCAC
Rhodococcus ruber Accession no: CCSD01000085.1	AATCCGAGGCGCAGCGCCTCGCGGACAACAGCGTCGGACGCGTGTGGCAC
Rhodococcus wratislaviensis Accession no:BHYM01000005.1	AATCCGAGGCGCAGCGCCTCGCGGACAACAGCGTCGGACGCGTGTGGCAC
***Rhodococcus sp.*SC4 SC4** Accession no: LSBM01000283.1	AATCCGAGGCGCAGCGCCTCGCGGACAACAGCGTCGGACGCGTGTGGCAC
Saccharothrix espanaensis Accession no: He804045.1	AGTCGACCGCGAGTCGCCTGGCCGACCCGCTGCGCGGCCGCACGTGGGTG
Sinomonas atrocyanea Accession no: CP014518.1	AGTCCCAGGCGGCGCGGGAGAACGACATGGCGGCCGGGCGCTCGTGGGTC
Streptomyces ossamyceticus Accession no: NZ_RJKY01000001.1	AGTCGGAGGCCCAGCGGGACGCCGACCTCGCCGTGGGCCGGGTGTGGCAC
Streptomyces fulvoviolaceus Accession no: NZ_JOEY01000080.1	AGTCGGAGGCCCAGCGGGACGCCGACCTCGCCGTGGGCCGGGTGTGGCAC
Streptomyces torulosus Accession no: NZ_LIRK01000041.1	AGTCGGAGGCCCAGCGGGACGCCGACCTCGCCGTGGGCCGGGTGTGGCAC
Streptomyces xylophagus Accession no: NZ_JNW001000006.1	AGTCGGAGGCCCAGCGGGACGCCGACCTCGCCGTGGGCCGGGTGTGGCAC
	* * * ** * ** ***
***Amycolatopsis thermoflava* N1165** Accessionn no: NZ_KI421511.1	ATCACCAACCCGGAGTCCCGCAACCGCCTCGGCGACCCCGTCGCCTACGC
***Amycolatopsis japonica* strain MG417-CF17** Accession no: CP008953.1	ATCACCAACCCGGAGTCCCGCAACCGCCTCGGCGACCCCGTCGCCTACGC
***Arthrobacter alpinus* ERGS4** Accession no: CP013200.1	ATTTCCAATCCAACGTCGCTGAATCGCCTGGGCGAGCCCGTGGGCTACAA
Arthrobacter sp. 31Y K253DRAFT Accession no: JAFW01000002.1	ATTTCCAATCCAACGTCGCTGAATCGCCTGGGCGAGCCCGTGGGCTACAA
Arthrobacter sp. M2012083 Accession no: NZ_AKKK01000062.1	ATTTCCAATCCAACGTCGCTGAATCGCCTGGGCGAGCCCGTGGGCTACAA
***Arthrobacter sp.* EpRS71** Accession no: LNUV01000006.1	ATTTCCAATCCAACGTCGCTGAATCGCCTGGGCGAGCCCGTGGGCTACAA
Arthrobacter crystallopoietes Accession no: AB240436.1	ATTTCCAATCCAACGTCGCTGAATCGCCTGGGCGAGCCCGTGGGCTACAA
Brevibacterium linens Accession no: CP014869.1	GTGACAAATCCGGAGTCGACCAACCGCCTCGGCGAACCGGTCGGCTACAA
***Brevibacterium sp.* YB235** Accession no: CP050153.1	GTGACAAATCCGGAGTCGACCAACCGCCTCGGCGAACCGGTCGGCTACAA
***Cryobacterium flavum* strain Hh8** Accession no: SOFD01000024.1	GTCTCCAACCCCAATTCCCTCAATCGCCTCGGCGAACCCGTCGCGTACAA
***Cryobacterium sp.* MLB32 c47** Accession no: JPRS01000047.1	GTCTCCAACCCCAATTCCCTCAATCGCCTCGGCGAACCCGTCGCGTACAA
***Cryobacterium luteum* strain Hh15** Accession no: SOFF01000030.1	GTCTCCAACCCCAATTCCCTCAATCGCCTCGGCGAACCCGTCGCGTACAA
Cryobacterium roopkundense Accession no: JPXF01000060.1	GTCTCCAACCCCAATTCCCTCAATCGCCTCGGCGAACCCGTCGCGTACAA
Kocuria indica Accession no: CP035504.1	GTCTCCAGCACCGAGCACACCAACCGGCTGGGCCACCCCGTGGGCTACAA
Kocuria flava Accession no: CP013254.1	GTCTCCAGCACCGAGCACACCAACCGGCTGGGCCACCCCGTGGGCTACAA
Microbacterium azadirachtae Accession no: JYIX 01000018.1	GTGCAGAGCGCCTCGCACACGAATGCCGTGGGCCGCGCGACCGCGTATCA
Microbacterium oxydans Accession no:CP031422.1	GTGCAGAGCGCCTCGCACACGAATGCCGTGGGCCGCGCGACCGCGTATCA
Microbacterium trichothecenolyticum Accession no: JYJA01000039.1	GTGCAGAGCGCCTCGCACACGAATGCCGTGGGCCGCGCGACCGCGTATCA
Microbacterium foliorum Accession no: JYIU01000019.1	GTGCAGAGCGCCTCGCACACGAATGCCGTGGGCCGCGCGACCGCGTATCA
Microbacterium mangrove Accession no: JTDK01000001.1	GTGCAGAGCGCCTCGCACACGAATGCCGTGGGCCGCGCGACCGCGTATCA
***Nocardiopsis sp.* NRRL B-16309** Accession no: LGEC01000081.1	ATCACCAACCCCGGCTCCCCCAACCGGCTCGGCCGCCACGTCGGCTACGC
Paenarthrobacter aurescens Accession no: CP000474.1	ATCTCCAACCCCGAGTCCAAAAACCGCCTGGGTGAGCCGGTCGGCTACAA

PseudArthrobacter sp. Accession no: CP041198.1	ATCTCCAACCCGGAATCCCGCAACCGGCTGGGCGAACCGGTGGGCTACAA
Pseudarthrobacter chlorophenolicus A6 Accession no: CP001341.1	ATCTCCAACCCGGAATCCCGCAACCGGCTGGGCGAACCGGTGGGCTACAA
***Rhodococcus jostii* RHA1** Accession no: CP000431.1	ATCAGCAACCCGAACAAGCTGAACCGTCTGGGCAAGCCGGTGGCGTACGC
***Rhodococcus opacus* 1CP** Accession no: CP009111.1	ATCAGCAACCCGAACAAGCTGAACCGTCTGGGCAAGCCGGTGGCGTACGC
Rhodococcus ruber Accession no: CCSD01000085.1	ATCAGCAACCCGAACAAGCTGAACCGTCTGGGCAAGCCGGTGGCGTACGC
Rhodococcus wratislaviensis Accession no:BHYM01000005.1	ATCAGCAACCCGAACAAGCTGAACCGTCTGGGCAAGCCGGTGGCGTACGC
Rhodococcus sp.SC4 SC4 Accession no: LSBM01000283.1	ATCAGCAACCCGAACAAGCTGAACCGTCTGGGCAAGCCGGTGGCGTACGC
Saccharothrix espanaensis Accession no: He804045.1	GTCAGCAGTTCCGACGAGGTGAACCGGGTGGGCAAGCCGCGCGCCTACCA
Sinomonas atrocyanea Accession no: CP014518.1	GTCTCCAACCCCGGCTCGCTCAACCGCCTCGGCGAGCCCGTGGCCTACAA
Streptomyces ossamyceticus Accession no: NZ_RJKY01000001.1	ATCTCCAACCCCGAGTCGCTCAACTCCCTCGGCGCGCCCGTCGGGTACAC
Streptomyces fulvoviolaceus Accession no: NZ_JOEY01000080.1	ATCTCCAACCCCGAGTCGCTCAACTCCCTCGGCGCGCCCGTCGGGTACAC
Streptomyces torulosus Accession no: NZ_LIRK01000041.1	ATCTCCAACCCCGAGTCGCTCAACTCCCTCGGCGCGCCCGTCGGGTACAC
Streptomyces xylophagus Accession no: NZ_JNW001000006.1	ATCTCCAACCCCGAGTCGCTCAACTCCCTCGGCGCGCCCGTCGGGTACAC
	* * * ** * ** * **
***Amycolatopsis thermoflava* N1165** Accessionn no: NZ_KI421511.1	ACTCGTCCCGGAAGGCAACCCGGAACTGCTCGCCGACGACGCGTCGTCCA
***Amycolatopsis japonica* strain MG417-CF17** Accession no: CP008953.1	ACTCGTCCCGGAAGGCAACCCGGAACTGCTCGCCGACGACGCGTCGTCCA
***Arthrobacter alpinus* ERGS4** Accession no: CP013200.1	GCTGCACCCTCAGGGCCAACCGATGCTGTTGGCGGACCCCGAATCATCGG
Arthrobacter sp. 31Y K253DRAFT Accession no: JAFW01000002.1	GCTGCACCCTCAGGGCCAACCGATGCTGTTGGCGGACCCCGAATCATCGG
Arthrobacter sp. M2012083 Accession no: NZ_AKKK01000062.1	GCTGCACCCTCAGGGCCAACCGATGCTGTTGGCGGACCCCGAATCATCGG
***Arthrobacter sp.* EpRS71** Accession no: LNUV01000006.1	GCTGCACCCTCAGGGCCAACCGATGCTGTTGGCGGACCCCGAATCATCGG
Arthrobacter crystallopoietes Accession no: AB240436.1	GCTGCACCCTCAGGGCCAACCGATGCTGTTGGCGGACCCCGAATCATCGG
Brevibacterium linens Accession no: CP014869.1	GCTGCACCCGATGGGCCTGCCCACACTGCTCGCCGCCGAGGATACGTCGA
***Brevibacterium sp.* YB235** Accession no: CP050153.1	GCTGCACCCGATGGGCCTGCCCACACTGCTCGCCGCCGAGGATACGTCGA
***Cryobacterium flavum* strain Hh8** Accession no: SOFD01000024.1	GCTGCACCCGCAGGGGCTGCCCGTGCTGCTGGCCGACCCGGAGTCGTCGG
***Cryobacterium sp.* MLB32 c47** Accession no: JPRS01000047.1	GCTGCACCCGCAGGGGCTGCCCGTGCTGCTGGCCGACCCGGAGTCGTCGG
***Cryobacterium luteum* strain Hh15** Accession no: SOFF01000030.1	GCTGCACCCGCAGGGGCTGCCCGTGCTGCTGGCCGACCCGGAGTCGTCGG
Cryobacterium roopkundense Accession no: JPXF01000060.1	GCTGCACCCGCAGGGGCTGCCCGTGCTGCTGGCCGACCCGGAGTCGTCGG
Kocuria indica Accession no: CP035504.1	GCTGCACCCGGAGGGGCTGCCCACCATGCTGGCCTCGGAGGACTCCTCGA
Kocuria flava Accession no: CP013254.1	GCTGCACCCGGAGGGGCTGCCCACCATGCTGGCCTCGGAGGACTCCTCGA
Microbacterium azadirachtae Accession no: JYIX 01000018.1	GCTCGTGCCGCAGCCGACCGCGCTGCTCATGGCGGATCCGGCGTCCTCCG
Microbacterium oxydans Accession no:CP031422.1	GCTCGTGCCGCAGCCGACCGCGCTGCTCATGGCGGATCCGGCGTCCTCCG
Microbacterium trichothecenolyticum Accession no: JYJA01000039.1	GCTCGTGCCGCAGCCGACCGCGCTGCTCATGGCGGATCCGGCGTCCTCCG
Microbacterium foliorum Accession no: JYIU01000019.1	GCTCGTGCCGCAGCCGACCGCGCTGCTCATGGCGGATCCGGCGTCCTCCG
Microbacterium mangrove Accession no: JTDK01000001.1	GCTCGTGCCGCAGCCGACCGCGCTGCTCATGGCGGATCCGGCGTCCTCCG
***Nocardiopsis sp.* NRRL B-16309** Accession no: LGEC01000081.1	CCTCCAGCCCGAGGGCAAGCCGGTCCTGCTCGCCGATCCCGCCTCGTCGG
Paenarthrobacter aurescens Accession no: CP000474.1	ACTCCACGCCGAGAACCAGCCCACGCTGCTGGCTGATCCCGGTTCCTCCA
PseudArthrobacter sp. Accession no: CP041198.1	GCTCCACTCCCACAACCAGCCCACGCTGCTCGCCGACCCGGAATCGTCCA
Pseudarthrobacter chlorophenolicus A6 Accession no: CP001341.1	GCTCCACTCCCACAACCAGCCCACGCTGCTCGCCGACCCGGAATCGTCCA
***Rhodococcus jostii* RHA1** Accession no: CP000431.1	ACTCCACCCGGAGGGTCAGCCGATCCTGCTGGCCGACGACGACTCCTCGA
***Rhodococcus opacus* 1CP** Accession no: CP009111.1	ACTCCACCCGGAGGGTCAGCCGATCCTGCTGGCCGACGACGACTCCTCGA
Rhodococcus ruber Accession no: CCSD01000085.1	ACTCCACCCGGAGGGTCAGCCGATCCTGCTGGCCGACGACGACTCCTCGA
Rhodococcus wratislaviensis Accession no:BHYM01000005.1	ACTCCACCCGGAGGGTCAGCCGATCCTGCTGGCCGACGACGACTCCTCGA
Rhodococcus sp.SC4 SC4 Accession no: LSBM01000283.1	ACTCCACCCGGAGGGTCAGCCGATCCTGCTGGCCGACGACGACTCCTCGA
Saccharothrix espanaensis Accession no: He804045.1	GCTCGTGCCCAAGCCCGGTCCGACGCTGCTCGCGCAGCCGGACTCGACGA
Sinomonas atrocyanea Accession no: CP014518.1	GATCCACCCCCAGGGCCAGCCGACGCTCCTGGCCGACCCGGCGTCGTCGG
Streptomyces ossamyceticus Accession no: NZ_RJKY01000001.1	CCTCCACCCGGAGGGCAGGCCGACCCTGCTCGCCGACCCGGGCTCGTCCA
Streptomyces fulvoviolaceus Accession no: NZ_JOEY01000080.1	CCTCCACCCGGAGGGCAGGCCGACCCTGCTCGCCGACCCGGGCTCGTCCA
Streptomyces torulosus Accession no: NZ_LIRK01000041.1	CCTCCACCCGGAGGGCAGGCCGACCCTGCTCGCCGACCCGGGCTCGTCCA
Streptomyces xylophagus Accession no: NZ_JNW001000006.1	CCTCCACCCGGAGGGCAGGCCGACCCTGCTCGCCGACCCGGGCTCGTCCA
	* * * * * ** * * *
***Amycolatopsis thermoflava* N1165** Accessionn no: NZ_KI421511.1	TCCATGCCCGCGCCACGTTCGCCACCAAGCACCTCTGGGTCACCCGCTAC
***Amycolatopsis japonica* strain MG417-CF17** Accession no: CP008953.1	TCCATGCCCGCGCCACGTTCGCCACCAAGCACCTCTGGGTCACCCGCTAC
***Arthrobacter alpinus* ERGS4** Accession no: CP013200.1	TGGCCCAGCGCGCTACATTTGCCACGAAGGACCTGTGGGTCACGCAATTT
Arthrobacter sp. 31Y K253DRAFT Accession no: JAFW01000002.1	TGGCCCAGCGCGCTACATTTGCCACGAAGGACCTGTGGGTCACGCAATTT

Arthrobacter sp. M2012083 Accession no: NZ_AKKK01000062.1	TGGCCCAGCGCGCTACATTTGCCACGAAGGACCTGTGGGTCACGCAATTT
***Arthrobacter sp.* EpRS71** Accession no: LNUV01000006.1	TGGCCCAGCGCGCTACATTTGCCACGAAGGACCTGTGGGTCACGCAATTT
Arthrobacter crystallopoietes Accession no: AB240436.1	TGGCCCAGCGCGCTACATTTGCCACGAAGGACCTGTGGGTCACGCAATTT
Brevibacterium linens Accession no: CP014869.1	TCCATCGCCGCGCCACGTTCGCGTCGAAGTCGCTGTGGGTCTCGCAGTAT
***Brevibacterium sp.* YB235** Accession no: CP050153.1	TCCATCGCCGCGCCACGTTCGCGTCGAAGTCGCTGTGGGTCTCGCAGTAT
***Cryobacterium flavum* strain Hh8** Accession no: SOFD01000024.1	TGGCCCAGCGCGCGGCGTTCGCCACCAAGGGCCTCTGGGTCACCCGCTTC
***Cryobacterium sp.* MLB32 c47** Accession no: JPRS01000047.1	TGGCCCAGCGCGCGGCGTTCGCCACCAAGGGCCTCTGGGTCACCCGCTTC
***Cryobacterium luteum* strain Hh15** Accession no: SOFF01000030.1	TGGCCCAGCGCGCGGCGTTCGCCACCAAGGGCCTCTGGGTCACCCGCTTC
Cryobacterium roopkundense Accession no: JPXF01000060.1	TGGCCCAGCGCGCGGCGTTCGCCACCAAGGGCCTCTGGGTCACCCGCTTC
Kocuria indica Accession no: CP035504.1	TCCACCGCCGCGCCGCGTTCGCCACCAAGGCCCTGTGGGTCACCCCGTAC
Kocuria flava Accession no: CP013254.1	TCCACCGCCGCGCCGCGTTCGCCACCAAGGCCCTGTGGGTCACCCCGTAC
Microbacterium azadirachtae Accession no: JYIX 01000018.1	TCGCGGCGCGCGCGGCGTTCGCGACCAAGCACCTCTGGGCCACCGCGCAC
Microbacterium oxydans Accession no:CP031422.1	TCGCGGCGCGCGCGGCGTTCGCGACCAAGCACCTCTGGGCCACCGCGCAC
Microbacterium trichothecenolyticum Accession no: JYJA01000039.1	TCGCGGCGCGCGCGGCGTTCGCGACCAAGCACCTCTGGGCCACCGCGCAC
Microbacterium foliorum Accession no: JYIU01000019.1	TCGCGGCGCGCGCGGCGTTCGCGACCAAGCACCTCTGGGCCACCGCGCAC
Microbacterium mangrove Accession no: JTDK01000001.1	TCGCGGCGCGCGCGGCGTTCGCGACCAAGCACCTCTGGGCCACCGCGCAC
***Nocardiopsis sp.* NRRL B-16309** Accession no: LGEC01000081.1	TGGCCGCGCGCGCCGCCTTCGCCACCCGCCACCTGTGGGTGACCGCCTAC
Paenarthrobacter aurescens Accession no: CP000474.1	TTGCGAAGCGTGCGGCTTTCGCCACCAAGGACGTATGGGTCACCCGCTAT
PseudArthrobacter sp. Accession no: CP041198.1	TCGCCCGCCGTGCCGCGTTCGCCACCAAGGACCTGTGGGTTACCCGCTAC
Pseudarthrobacter chlorophenolicus A6 Accession no: CP001341.1	TCGCCCGCCGTGCCGCGTTCGCCACCAAGGACCTGTGGGTTACCCGCTAC
***Rhodococcus jostii* RHA1** Accession no: CP000431.1	TCGCGGCGCGGGCGACGTTCGCCACCAAGCACCTGTGGGTCACCCAGTTC
***Rhodococcus opacus* 1CP** Accession no: CP009111.1	TCGCGGCGCGGGCGACGTTCGCCACCAAGCACCTGTGGGTCACCCAGTTC
Rhodococcus ruber Accession no: CCSD01000085.1	TCGCGGCGCGGGCGACGTTCGCCACCAAGCACCTGTGGGTCACCCAGTTC
Rhodococcus wratislaviensis Accession no:BHYM01000005.1	TCGCGGCGCGGGCGACGTTCGCCACCAAGCACCTGTGGGTCACCCAGTTC
***Rhodococcus sp.*SC4 SC4** Accession no: LSBM01000283.1	TCGCGGCGCGGGCGACGTTCGCCACCAAGCACCTGTGGGTCACCCAGTTC
Saccharothrix espanaensis Accession no: He804045.1	TCGCGGCCCGCGCCGGGTTCGCGTCCCGACACCTGTGGGTGACCCGCTTC
Sinomonas atrocyanea Accession no: CP014518.1	TCGCCGGCCGCGCCGCGTTCGCGACCAAGGCCCTCTGGGTGACCCGCTAC
Streptomyces ossamyceticus Accession no: NZ_RJKY01000001.1	TCGCCGCCCGCGCCGCCTTCGCCACCCGGCATCTGTGGGTCACCGCCTAC
Streptomyces fulvoviolaceus Accession no: NZ_JOEY01000080.1	TCGCCGCCCGCGCCGCCTTCGCCACCCGGCATCTGTGGGTCACCGCCTAC
Streptomyces torulosus Accession no: NZ_LIRK01000041.1	TCGCCGCCCGCGCCGCCTTCGCCACCCGGCATCTGTGGGTCACCGCCTAC
Streptomyces xylophagus Accession no: NZ_JNW001000006.1	TCGCCGCCCGCGCCGCCTTCGCCACCCGGCATCTGTGGGTCACCGCCTAC
	* ** ** ** ** * * **** *
***Amycolatopsis thermoflava* N1165** Accessionn no: NZ_KI421511.1	GACCCCGCGCAGCGCTACGCGGCAGGCGACTTCGTCAACCAGCACCCCGG
***Amycolatopsis japonica* strain MG417-CF17** Accession no: CP008953.1	GACCCCGCGCAGCGCTACGCGGCAGGCGACTTCGTCAACCAGCACCCCGG
***Arthrobacter alpinus* ERGS4** Accession no: CP013200.1	TCCGAGGATGAGCGCTACCCCACGGGTGACTTTGTCAACCAGCACGGCGG
Arthrobacter sp. 31Y K253DRAFT Accession no: JAFW01000002.1	TCCGAGGATGAGCGCTACCCCACGGGTGACTTTGTCAACCAGCACGGCGG
Arthrobacter sp. M2012083 Accession no: NZ_AKKK01000062.1	TCCGAGGATGAGCGCTACCCCACGGGTGACTTTGTCAACCAGCACGGCGG
***Arthrobacter sp.* EpRS71** Accession no: LNUV01000006.1	TCCGAGGATGAGCGCTACCCCACGGGTGACTTTGTCAACCAGCACGGCGG
Arthrobacter crystallopoietes Accession no: AB240436.1	TCCGAGGATGAGCGCTACCCCACGGGTGACTTTGTCAACCAGCACGGCGG
Brevibacterium linens Accession no: CP014869.1	CACGAGGACGAACGCTATCCGACCGGGGACTTCCCGAATCAGCATCCCGG
***Brevibacterium sp.* YB235** Accession no: CP050153.1	CACGAGGACGAACGCTATCCGACCGGGGACTTCCCGAATCAGCATCCCGG
***Cryobacterium flavum* strain Hh8** Accession no: SOFD01000024.1	GCCGAAGACGAACGCTATCCCACCGGTGACTTCGTCAACCAGCACGCCGG
***Cryobacterium sp.* MLB32 c47** Accession no: JPRS01000047.1	GCCGAAGACGAACGCTATCCCACCGGTGACTTCGTCAACCAGCACGCCGG
***Cryobacterium luteum* strain Hh15** Accession no: SOFF01000030.1	GCCGAAGACGAACGCTATCCCACCGGTGACTTCGTCAACCAGCACGCCGG
Cryobacterium roopkundense Accession no: JPXF01000060.1	GCCGAAGACGAACGCTATCCCACCGGTGACTTCGTCAACCAGCACGCCGG
Kocuria indica Accession no: CP035504.1	GAGGAGTCCGAGCGGTACCCCACCGGCGACTTCCCCAACCAGCACGCCGG
Kocuria flava Accession no: CP013254.1	GAGGAGTCCGAGCGGTACCCCACCGGCGACTTCCCCAACCAGCACGCCGG
Microbacterium azadirachtae Accession no: JYIX 01000018.1	CGCGAGGGCGAGCTGTGGCCCGCCGGCCGCTATCCGAACGCGCACCAGGG
Microbacterium oxydans Accession no:CP031422.1	CGCGAGGGCGAGCTGTGGCCCGCCGGCCGCTATCCGAACGCGCACCAGGG
Microbacterium trichothecenolyticum Accession no: JYJA01000039.1	CGCGAGGGCGAGCTGTGGCCCGCCGGCCGCTATCCGAACGCGCACCAGGG
Microbacterium foliorum Accession no: JYIU01000019.1	CGCGAGGGCGAGCTGTGGCCCGCCGGCCGCTATCCGAACGCGCACCAGGG
Microbacterium mangrove Accession no: JTDK01000001.1	CGCGAGGGCGAGCTGTGGCCCGCCGGCCGCTATCCGAACGCGCACCAGGG
***Nocardiopsis sp.* NRRL B-16309** Accession no: LGEC01000081.1	GATCCCGCCGAGCGCTACCCGGCGGGCGACTTCGTCAACCAGCACCCGGG
Paenarthrobacter aurescens Accession no: CP000474.1	GCCGACGAGGAACGCTACCCCACGGGCGACTTCGTGAACCAGCACTCCGG
PseudArthrobacter sp. Accession no: CP041198.1	GCCGAGGAGGAGCGCTACCCCACGGGCGACTTCGTGAACCAGCACTCCGG

Pseudarthrobacter chlorophenolicus A6 Accession no: CP001341.1	GCCGAGGAGGAGCGCTACCCCACGGGCGACTTCGTGAACCAGCACTCCGG
Rhodococcus jostii **RHA1** Accession no: CP000431.1	GACGAGAAGGAGCGTTACGCGGCAGGGGATTTCGTGAACCAGCACGCGGG
Rhodococcus opacus **1CP** Accession no: CP009111.1	GACGAGAAGGAGCGTTACGCGGCAGGGGATTTCGTGAACCAGCACGCGGG
Rhodococcus ruber Accession no: CCSD01000085.1	GACGAGAAGGAGCGTTACGCGGCAGGGGATTTCGTGAACCAGCACGCGGG
Rhodococcus wratislaviensis Accession no:BHYM01000005.1	GACGAGAAGGAGCGTTACGCGGCAGGGGATTTCGTGAACCAGCACGCGGG
Rhodococcus sp.SC4 SC4 Accession no: LSBM01000283.1	GACGAGAAGGAGCGTTACGCGGCAGGGGATTTCGTGAACCAGCACGCGGG
Saccharothrix espanaensis Accession no: He804045.1	CACGAGGACGAGCGCTACCCGGCGGGCGAGTACCCCGACCAGCACCCCGG
Sinomonas atrocyanea Accession no: CP014518.1	GACGAGGCGGAGCGGTACCCCACCGGCGACTTCGTGAACCAGCACCCCGG
Streptomyces ossamyceticus Accession no: NZ_RJKY01000001.1	GATCCCGCCGAGCGCTACCCCGCCGGCGACTTCGTCAACCAGCATCCCGG
Streptomyces fulvoviolaceus Accession no: NZ_JOEY01000080.1	GATCCCGCCGAGCGCTACCCCGCCGGCGACTTCGTCAACCAGCATCCCGG
Streptomyces torulosus Accession no: NZ_LIRK01000041.1	GATCCCGCCGAGCGCTACCCCGCCGGCGACTTCGTCAACCAGCATCCCGG
Streptomyces xylophagus Accession no: NZ_JNW001000006.1	GATCCCGCCGAGCGCTACCCCGCCGGCGACTTCGTCAACCAGCATCCCGG
	* * * * * ** * * *** **
Amycolatopsis thermoflava **N1165** Accessionn no: NZ_KI421511.1	TGGCGCCGGGCTGCCCGCCTACGTGGCCGGTGAC---CGCGAC------C
Amycolatopsis japonica **strain MG417-CF17** Accession no: CP008953.1	TGGCGCCGGGCTGCCCGCCTACGTGGCCGGTGAC---CGCGAC------C
Arthrobacter alpinus **ERGS4** Accession no: CP013200.1	CGGCGCGGGCCTGCCGGCTTACATTGCCGGCAAC---CGAGAG------T
Arthrobacter sp. 31Y K253DRAFT Accession no: JAFW01000002.1	CGGCGCGGGCCTGCCGGCTTACATTGCCGGCAAC---CGAGAG------T
Arthrobacter sp. M2012083 Accession no: NZ_AKKK01000062.1	CGGCGCGGGCCTGCCGGCTTACATTGCCGGCAAC---CGAGAG------T
Arthrobacter sp. **EpRS71** Accession no: LNUV01000006.1	CGGCGCGGGCCTGCCGGCTTACATTGCCGGCAAC---CGAGAG------T
Arthrobacter crystallopoietes Accession no: AB240436.1	CGGCGCGGGCCTGCCGGCTTACATTGCCGGCAAC---CGAGAG------T
Brevibacterium linens Accession no: CP014869.1	CCACGCGGGACTTCCGGCGTGGACGGCGGCCGAT---CGCAGC------G
Brevibacterium sp. **YB235** Accession no: CP050153.1	CCACGCGGGACTTCCGGCGTGGACGGCGGCCGAT---CGCAGC------G
Cryobacterium flavum **strain Hh8** Accession no: SOFD01000024.1	CGGCGCCGGGCTGCCCGCCTACATTGCGGCCGAC---CGAGCG------A
Cryobacterium sp. **MLB32 c47** Accession no: JPRS01000047.1	CGGCGCCGGGCTGCCCGCCTACATTGCGGCCGAC---CGAGCG------A
Cryobacterium luteum **strain Hh15** Accession no: SOFF01000030.1	CGGCGCCGGGCTGCCCGCCTACATTGCGGCCGAC---CGAGCG------A
Cryobacterium roopkundense Accession no: JPXF01000060.1	CGGCGCCGGGCTGCCCGCCTACATTGCGGCCGAC---CGAGCG------A
Kocuria indica Accession no: CP035504.1	TAACGGCGGCCTGCCGGAGTGGACCGCCCAGGAC---CGCTCG------G
Kocuria flava Accession no: CP013254.1	TAACGGCGGCCTGCCGGAGTGGACCGCCCAGGAC---CGCTCG------G
Microbacterium azadirachtae Accession no: JYIX 01000018.1	CGGGTCGGGCCTGCCCGAGTACTCCGCGAGCGACGCCCCCTTGGACGGCC
Microbacterium oxydans Accession no:CP031422.1	CGGGTCGGGCCTGCCCGAGTACTCCGCGAGCGACGCCCCCTTGGACGGCC
Microbacterium trichothecenolyticum Accession no: JYJA01000039.1	CGGGTCGGGCCTGCCCGAGTACTCCGCGAGCGACGCCCCCTTGGACGGCC
Microbacterium foliorum Accession no: JYIU01000019.1	CGGGTCGGGCCTGCCCGAGTACTCCGCGAGCGACGCCCCCTTGGACGGCC
Microbacterium mangrove Accession no: JTDK01000001.1	CGGGTCGGGCCTGCCCGAGTACTCCGCGAGCGACGCCCCCTTGGACGGCC
Nocardiopsis sp. **NRRL B-16309** Accession no: LGEC01000081.1	CGGCGCCGGCCTGCCCGCCTACGCCGCCGCCGAC---CGCGAC------C
Paenarthrobacter aurescens Accession no: CP000474.1	CGGCGCCGGTTTGCCGGCCTATGTGGCCCAGGAC---CGCGAC------A
PseudArthrobacter sp. Accession no: CP041198.1	CGGCGCCGGGCTCCCCGCCTACGTGGCACAGGAC---CGGGAC------A
Pseudarthrobacter chlorophenolicus A6 Accession no: CP001341.1	CGGCGCCGGGCTCCCCGCCTACGTGGCACAGGAC---CGGGAC------A
Rhodococcus jostii **RHA1** Accession no: CP000431.1	TGGCGCCGGACTGCCCAGCTTCGTGGCCGGCGAT---CGGGAT------C
Rhodococcus opacus **1CP** Accession no: CP009111.1	TGGCGCCGGACTGCCCAGCTTCGTGGCCGGCGAT---CGGGAT------C
Rhodococcus ruber Accession no: CCSD01000085.1	TGGCGCCGGACTGCCCAGCTTCGTGGCCGGCGAT---CGGGAT------C
Rhodococcus wratislaviensis Accession no:BHYM01000005.1	TGGCGCCGGACTGCCCAGCTTCGTGGCCGGCGAT---CGGGAT------C
Rhodococcus sp.SC4 SC4 Accession no: LSBM01000283.1	TGGCGCCGGACTGCCCAGCTTCGTGGCCGGCGAT---CGGGAT------C
Saccharothrix espanaensis Accession no: He804045.1	TGGCGCGGGGCTGCCGGCCTGGGCCGCGCAGGAC---CGCCCG------C
Sinomonas atrocyanea Accession no: CP014518.1	GGGAGCCGGCCTGCCGGCCTACCAGACCCAGGAC---CGCGAC------C
Streptomyces ossamyceticus Accession no: NZ_RJKY01000001.1	CGGCGCGGGTCTGCCCGCCTACACGGCCGCGGAC---CGTTCG------C
Streptomyces fulvoviolaceus Accession no: NZ_JOEY01000080.1	CGGCGCGGGTCTGCCCGCCTACACGGCCGCGGAC---CGTTCG------C
Streptomyces torulosus Accession no: NZ_LIRK01000041.1	CGGCGCGGGTCTGCCCGCCTACACGGCCGCGGAC---CGTTCG------C
Streptomyces xylophagus Accession no: NZ_JNW001000006.1	CGGCGCGGGTCTGCCCGCCTACACGGCCGCGGAC---CGTTCG------C
	** * ** * * * *
Amycolatopsis thermoflava **N1165** Accessionn no: NZ_KI421511.1	TCGACGGGCAGGACCTCGTCGTGTGGCACACCTTCGGCCTCACGCACTTC
Amycolatopsis japonica **strain MG417-CF17** Accession no: CP008953.1	TCGACGGGCAGGACCTCGTCGTGTGGCACACCTTCGGCCTCACGCACTTC
Arthrobacter alpinus **ERGS4** Accession no: CP013200.1	TGGACGGTGAGGACCTGGTGGTGTGGCACACCTTTGGCCTGACTCACTTC
Arthrobacter sp. 31Y K253DRAFT Accession no: JAFW01000002.1	TGGACGGTGAGGACCTGGTGGTGTGGCACACCTTTGGCCTGACTCACTTC
Arthrobacter sp. M2012083 Accession no: NZ_AKKK01000062.1	TGGACGGTGAGGACCTGGTGGTGTGGCACACCTTTGGCCTGACTCACTTC

Arthrobacter sp. **EpRS71** Accession no: LNUV01000006.1	TGGACGGTGAGGACCTGGTGGTGTGGCACACCTTTGGCCTGACTCACTTC
Arthrobacter crystallopoietes Accession no: AB240436.1	TGGACGGTGAGGACCTGGTGGTGTGGCACACCTTTGGCCTGACTCACTTC
Brevibacterium linens Accession no: CP014869.1	TCGACGGTGAGGAGATCGTGGTCTGGCACAGCTTCGGTCTCACGCACTTC
Brevibacterium sp. **YB235** Accession no: CP050153.1	TCGACGGTGAGGAGATCGTGGTCTGGCACAGCTTCGGTCTCACGCACTTC
Cryobacterium flavum **strain Hh8** Accession no: SOFD01000024.1	TCGACGGCGAGGACATCGTGCTCTGGCATACCTTCGGCCTCACCCACTTT
Cryobacterium sp. **MLB32 c47** Accession no: JPRS01000047.1	TCGACGGCGAGGACATCGTGCTCTGGCATACCTTCGGCCTCACCCACTTT
Cryobacterium luteum **strain Hh15** Accession no: SOFF01000030.1	TCGACGGCGAGGACATCGTGCTCTGGCATACCTTCGGCCTCACCCACTTT
Cryobacterium roopkundense Accession no: JPXF01000060.1	TCGACGGCGAGGACATCGTGCTCTGGCATACCTTCGGCCTCACCCACTTT
Kocuria indica Accession no: CP035504.1	TGGACGGCACGGACATCGTGGTGTGGCACACCTTCGGGCTCACCCACTTC
Kocuria flava Accession no: CP013254.1	TGGACGGCACGGACATCGTGGTGTGGCACACCTTCGGGCTCACCCACTTC
Microbacterium azadirachtae Accession no: JYIX 01000018.1	CTGATGGCGCCGACCTCGTGCTCTGGCACACGTTCGGACTCACGCACTTC
Microbacterium oxydans Accession no:CP031422.1	CTGATGGCGCCGACCTCGTGCTCTGGCACACGTTCGGACTCACGCACTTC
Microbacterium trichothecenolyticum Accession no: JYJA01000039.1	CTGATGGCGCCGACCTCGTGCTCTGGCACACGTTCGGACTCACGCACTTC
Microbacterium foliorum Accession no: JYIU01000019.1	CTGATGGCGCCGACCTCGTGCTCTGGCACACGTTCGGACTCACGCACTTC
Microbacterium mangrove Accession no: JTDK01000001.1	CTGATGGCGCCGACCTCGTGCTCTGGCACACGTTCGGACTCACGCACTTC
Nocardiopsis sp. **NRRL B-16309** Accession no: LGEC01000081.1	TGGACGGCCGGGACCTGGTCGTGTGGCACACCTTCGGCCTGACGCACGCG
Paenarthrobacter aurescens Accession no: CP000474.1	TCGACGGCCAGGACATCGTCCTCTGGCACACCTTCGGCCTCACTCACTTC
PseudArthrobacter sp. Accession no: CP041198.1	TCGACGGCCAGGACATCGTGGTGTGGCACACCTTTGGCCTGACCCACTTC
Pseudarthrobacter chlorophenolicus A6 Accession no: CP001341.1	TCGACGGCCAGGACATCGTGGTGTGGCACACCTTTGGCCTGACCCACTTC
Rhodococcus jostii **RHA1** Accession no: CP000431.1	TGGAGAACGAAGACGTCGTCGTCTGGCACACGTTCGGCCTCACGCACTTC
Rhodococcus opacus **1CP** Accession no: CP009111.1	TGGAGAACGAAGACGTCGTCGTCTGGCACACGTTCGGCCTCACGCACTTC
Rhodococcus ruber Accession no: CCSD01000085.1	TGGAGAACGAAGACGTCGTCGTCTGGCACACGTTCGGCCTCACGCACTTC
Rhodococcus wratislaviensis Accession no:BHYM01000005.1	TGGAGAACGAAGACGTCGTCGTCTGGCACACGTTCGGCCTCACGCACTTC
***Rhodococcus sp.*SC4 SC4** Accession no: LSBM01000283.1	TGGAGAACGAAGACGTCGTCGTCTGGCACACGTTCGGCCTCACGCACTTC
Saccharothrix espanaensis Accession no: He804045.1	TGGTGGACGAGGACGTCGTGCTCTGGCACGTTTTCGGCCCCACCCACCTG
Sinomonas atrocyanea Accession no: CP014518.1	TCGACGGGACCGACCTCGTCGTGTGGCACACGTTCGGCCTGACCCACTTC
Streptomyces ossamyceticus Accession no: NZ_RJKY01000001.1	TGGACGGCCGGTCGCTCGTCGTGTGGCACACCTTCGGCCTCACGCACGCA
Streptomyces fulvoviolaceus Accession no: NZ_JOEY01000080.1	TGGACGGCCGGTCGCTCGTCGTGTGGCACACCTTCGGCCTCACGCACGCA
Streptomyces torulosus Accession no: NZ_LIRK01000041.1	TGGACGGCCGGTCGCTCGTCGTGTGGCACACCTTCGGCCTCACGCACGCA
Streptomyces xylophagus Accession no: NZ_JNW001000006.1	TGGACGGCCGGTCGCTCGTCGTGTGGCACACCTTCGGCCTCACGCACGCA
	* * ** * ***** ** ** * ** ***
Amycolatopsis thermoflava **N1165** Accessionn no: NZ_KI421511.1	CCCCGCCCGGAGGACTGGCCGATCATGCCGGTCGACTACACCGGCTTCAA
Amycolatopsis japonica **strain MG417-CF17** Accession no: CP008953.1	CCCCGCCCGGAGGACTGGCCGATCATGCCGGTCGACTACACCGGCTTCAA
Arthrobacter alpinus **ERGS4** Accession no: CP013200.1	CCCCGCTTGGAAGACTGGCCCATCATGCCCGTCGACACCGTGGGCTTCAA
Arthrobacter sp. 31Y K253DRAFT Accession no: JAFW01000002.1	CCCCGCTTGGAAGACTGGCCCATCATGCCCGTCGACACCGTGGGCTTCAA
Arthrobacter sp. M2012083 Accession no: NZ_AKKK01000062.1	CCCCGCTTGGAAGACTGGCCCATCATGCCCGTCGACACCGTGGGCTTCAA
Arthrobacter sp. **EpRS71** Accession no: LNUV01000006.1	CCCCGCTTGGAAGACTGGCCCATCATGCCCGTCGACACCGTGGGCTTCAA
Arthrobacter crystallopoietes Accession no: AB240436.1	CCCCGCTTGGAAGACTGGCCCATCATGCCCGTCGACACCGTGGGCTTCAA
Brevibacterium linens Accession no: CP014869.1	CCGCGCGTCGAGGACTGGCCGATCATGCCCGTCGACACCGTCGGCTTCAA
Brevibacterium sp. **YB235** Accession no: CP050153.1	CCGCGCGTCGAGGACTGGCCGATCATGCCCGTCGACACCGTCGGCTTCAA
Cryobacterium flavum **strain Hh8** Accession no: SOFD01000024.1	CCGCGGGTCGAGGACTGGCCGATCATGCCGGTCGACACCGTGGGCTTCAA
Cryobacterium sp. **MLB32 c47** Accession no: JPRS01000047.1	CCGCGGGTCGAGGACTGGCCGATCATGCCGGTCGACACCGTGGGCTTCAA
Cryobacterium luteum **strain Hh15** Accession no: SOFF01000030.1	CCGCGGGTCGAGGACTGGCCGATCATGCCGGTCGACACCGTGGGCTTCAA
Cryobacterium roopkundense Accession no: JPXF01000060.1	CCGCGGGTCGAGGACTGGCCGATCATGCCGGTCGACACCGTGGGCTTCAA
Kocuria indica Accession no: CP035504.1	CCGCGCGTGGAGGACTGGCCGATCATGCCCGTGGACACCGTGGGCTTCAA
Kocuria flava Accession no: CP013254.1	CCGCGCGTGGAGGACTGGCCGATCATGCCCGTGGACACCGTGGGCTTCAA
Microbacterium azadirachtae Accession no: JYIX 01000018.1	CCGCGCCCGGAGGACTGGCCCATCATGCCCGTCGACACCGCGGGCTTCGC
Microbacterium oxydans Accession no:CP031422.1	CCGCGCCCGGAGGACTGGCCCATCATGCCCGTCGACACCGCGGGCTTCGC
Microbacterium trichothecenolyticum Accession no: JYJA01000039.1	CCGCGCCCGGAGGACTGGCCCATCATGCCCGTCGACACCGCGGGCTTCGC
Microbacterium foliorum Accession no: JYIU01000019.1	CCGCGCCCGGAGGACTGGCCCATCATGCCCGTCGACACCGCGGGCTTCGC
Microbacterium mangrove Accession no: JTDK01000001.1	CCGCGCCCGGAGGACTGGCCCATCATGCCCGTCGACACCGCGGGCTTCGC
Nocardiopsis sp. **NRRL B-16309** Accession no: LGEC01000081.1	CCCCGCCCGGAGGACTGGCCGATCATGCCGGTCGACCACACCGGCTTCAC
Paenarthrobacter aurescens Accession no: CP000474.1	CCGCGGGTGGAGGACTGGCCCATCATGCCCGTGGACACCGTCGGCTTCAA
PseudArthrobacter sp. Accession no: CP041198.1	CCGCGGGTGGAGGACTGGCCCATCATGCCCGTGGACACGGTGGGCTTCAA
Pseudarthrobacter chlorophenolicus A6 Accession no: CP001341.1	CCGCGGGTGGAGGACTGGCCCATCATGCCCGTGGACACGGTGGGCTTCAA

***Rhodococcus jostii* RHA1** Accession no: CP000431.1	CCGCGACCCGAGGACTGGCCGATCATGCCCGTCGACTACACCGGATTCAC
***Rhodococcus opacus* 1CP** Accession no: CP009111.1	CCGCGACCCGAGGACTGGCCGATCATGCCCGTCGACTACACCGGATTCAC
Rhodococcus ruber Accession no: CCSD01000085.1	CCGCGACCCGAGGACTGGCCGATCATGCCCGTCGACTACACCGGATTCAC
Rhodococcus wratislaviensis Accession no:BHYM01000005.1	CCGCGACCCGAGGACTGGCCGATCATGCCCGTCGACTACACCGGATTCAC
***Rhodococcus sp.SC4* SC4** Accession no: LSBM01000283.1	CCGCGACCCGAGGACTGGCCGATCATGCCCGTCGACTACACCGGATTCAC
Saccharothrix espanaensis Accession no: He804045.1	CCCCGCCCGGAGGACTGGCCGGTCATGCCGGTCGATTACAGCGGATTCAT
Sinomonas atrocyanea Accession no: CP014518.1	CCGCGCCCGGAGGACTGGCCCATCATGCCGGTGGACCACGTGGGCTTCAA
Streptomyces ossamyceticus Accession no: NZ_RJKY01000001.1	CCCCGGCCGGAGGACTGGCCGATCATGCCCGTCGACCACACGGGCTTCAA
Streptomyces fulvoviolaceus Accession no: NZ_JOEY01000080.1	CCCCGGCCGGAGGACTGGCCGATCATGCCCGTCGACCACACGGGCTTCAA
Streptomyces torulosus Accession no: NZ_LIRK01000041.1	CCCCGGCCGGAGGACTGGCCGATCATGCCCGTCGACCACACGGGCTTCAA
Streptomyces xylophagus Accession no: NZ_JNW001000006.1	CCCCGGCCGGAGGACTGGCCGATCATGCCCGTCGACCACACGGGCTTCAA
	** ** ** ********* ******* ** ** ** ***
***Amycolatopsis thermoflava* N1165** Accessionn no: NZ_KI421511.1	GCTCAAACCGGTCGGGTTCTTCGACCGCAACCCCGCCCTCGACGTGCCGC
***Amycolatopsis japonica* strain MG417-CF17** Accession no: CP008953.1	GCTCAAACCGGTCGGGTTCTTCGACCGCAACCCCGCCCTCGACGTGCCGC
***Arthrobacter alpinus* ERGS4** Accession no: CP013200.1	GCTCCGCCCCGAGGGGTTCTTTGACCGCAGCCCCGTGCTGGACGTGCCAG
***Arthrobacter sp.* 31Y K253DRAFT** Accession no: JAFW01000002.1	GCTCCGCCCCGAGGGGTTCTTTGACCGCAGCCCCGTGCTGGACGTGCCAG
***Arthrobacter sp.* M2012083** Accession no: NZ_AKKK01000062.1	GCTCCGCCCCGAGGGGTTCTTTGACCGCAGCCCCGTGCTGGACGTGCCAG
***Arthrobacter sp.* EpRS71** Accession no: LNUV01000006.1	GCTCCGCCCCGAGGGGTTCTTTGACCGCAGCCCCGTGCTGGACGTGCCAG
Arthrobacter crystallopoietes Accession no: AB240436.1	GCTCCGCCCCGAGGGGTTCTTTGACCGCAGCCCCGTGCTGGACGTGCCAG
Brevibacterium linens Accession no: CP014869.1	GCTGCGCCCCGAAGGATTCTTCGACCGCTCCCCTGTCCTCGACGTCCCCT
***Brevibacterium sp.* YB235** Accession no: CP050153.1	GCTGCGCCCCGAAGGATTCTTCGACCGCTCCCCTGTCCTCGACGTCCCCT
***Cryobacterium flavum* strain Hh8** Accession no: SOFD01000024.1	GCTGCGCCCCGACGGCTTCTTCGACCGCAGCCCAGTGCTGGACGTCCCCG
***Cryobacterium sp.* MLB32 c47** Accession no: JPRS01000047.1	GCTGCGCCCCGACGGCTTCTTCGACCGCAGCCCAGTGCTGGACGTCCCCG
***Cryobacterium luteum* strain Hh15** Accession no: SOFF01000030.1	GCTGCGCCCCGACGGCTTCTTCGACCGCAGCCCAGTGCTGGACGTCCCCG
Cryobacterium roopkundense Accession no: JPXF01000060.1	GCTGCGCCCCGACGGCTTCTTCGACCGCAGCCCAGTGCTGGACGTCCCCG
Kocuria indica Accession no: CP035504.1	GCTGCGCCCGGAGGGCTTCTTCGACCGCTCCCCGGTGCTGGACGTCCCCG
Kocuria flava Accession no: CP013254.1	GCTGCGCCCGGAGGGCTTCTTCGACCGCTCCCCGGTGCTGGACGTCCCCG
Microbacterium azadirachtae Accession no: JYIX 01000018.1	GTTCCGCCCCTACGGCTTCCTCGACCAGAACCCGGGCATGGACGTGCCGG
Microbacterium oxydans Accession no:CP031422.1	GTTCCGCCCCTACGGCTTCCTCGACCAGAACCCGGGCATGGACGTGCCGG
Microbacterium trichothecenolyticum Accession no: JYJA01000039.1	GTTCCGCCCCTACGGCTTCCTCGACCAGAACCCGGGCATGGACGTGCCGG
Microbacterium foliorum Accession no: JYIU01000019.1	GTTCCGCCCCTACGGCTTCCTCGACCAGAACCCGGGCATGGACGTGCCGG
Microbacterium mangrove Accession no: JTDK01000001.1	GTTCCGCCCCTACGGCTTCCTCGACCAGAACCCGGGCATGGACGTGCCGG
***Nocardiopsis sp.* NRRL B-16309** Accession no: LGEC01000081.1	GCTGCGCCCGGTCGGCTTCTTCGACCGCAACCCCACCCTGGACGTGCCCG
Paenarthrobacter aurescens Accession no: CP000474.1	ACTCCGTCCGGAAGGCTTCTTTGACCGCAGCCCGGTCCTGGACGTGCCCG
PseudArthrobacter sp. Accession no: CP041198.1	GCTCCGCCCAGAGGGCTTCTTCGACCGCAGCCCCGTCCTCGACGTCCCCG
Pseudarthrobacter chlorophenolicus A6 Accession no: CP001341.1	GCTCCGCCCAGAGGGCTTCTTCGACCGCAGCCCCGTCCTCGACGTCCCCG
***Rhodococcus jostii* RHA1** Accession no: CP000431.1	GTTGAAGCCCAACGGCTTCTTCGATCGCAACCCCGCTCTCGACGTTCCCC
***Rhodococcus opacus* 1CP** Accession no: CP009111.1	GTTGAAGCCCAACGGCTTCTTCGATCGCAACCCCGCTCTCGACGTTCCCC
Rhodococcus ruber Accession no: CCSD01000085.1	GTTGAAGCCCAACGGCTTCTTCGATCGCAACCCCGCTCTCGACGTTCCCC
Rhodococcus wratislaviensis Accession no:BHYM01000005.1	GTTGAAGCCCAACGGCTTCTTCGATCGCAACCCCGCTCTCGACGTTCCCC
***Rhodococcus sp.SC4* SC4** Accession no: LSBM01000283.1	GTTGAAGCCCAACGGCTTCTTCGATCGCAACCCCGCTCTCGACGTTCCCC
Saccharothrix espanaensis Accession no: He804045.1	GTTCAAGCCGCTGGGATTCCTGGACCGCAATCCCACGCTCGACCTGCCGG
Sinomonas atrocyanea Accession no: CP014518.1	GATCCGCCCCGAGGGCTTCTTCGACCGCAGCCCCGTCCTCGACGTCCCGG
Streptomyces ossamyceticus Accession no: NZ_RJKY01000001.1	GCTCACCCCGACCGGCTTCTTCGACCGCAACCCGACGTTGGACGTCCCGC
Streptomyces fulvoviolaceus Accession no: NZ_JOEY01000080.1	GCTCACCCCGACCGGCTTCTTCGACCGCAACCCGACGTTGGACGTCCCGC
Streptomyces torulosus Accession no: NZ_LIRK01000041.1	GCTCACCCCGACCGGCTTCTTCGACCGCAACCCGACGTTGGACGTCCCGC
Streptomyces xylophagus Accession no: NZ_JNW001000006.1	GCTCACCCCGACCGGCTTCTTCGACCGCAACCCGACGTTGGACGTCCCGC
	* ** ** *** * ** * ** * *** * **
